# Synthesis and in silico studies of quinoline appended acridine via conventional and green methods: photophysical analysis of novel fluorophore for picric acid detection using a ‘turn-off' fluorescence approach

**DOI:** 10.1186/s13065-025-01452-y

**Published:** 2025-04-09

**Authors:** Rebecca Susan Philip, V. Vijayakumar

**Affiliations:** Department of Chemistry, School of Advanced Sciences, Vellore Institute of Technology, Vellore, India 632014

**Keywords:** Quinoline, Deep eutectic solvent, TD-DFT, Bovine serum albumin, Fluorescence sensor, Picric acid, Fluorescence quenching

## Abstract

A series of novel 4-(9-phenyl-1,2,3,4-tetrahydroacridin-2-yl)cyclohexan-1-ones and their dimers were synthesized using an efficient one-pot method with Deep Eutectic Solvents (DESs), alongside microwave-assisted and conventional techniques. Using less toxic and inexpensive DESs enhance sustainability in producing desired products. Green metrics calculations indicate a high level of greenness in the synthesis process. FT-IR, NMR, and HRMS characterized the compounds. In-silico tests involving Bovine Serum Albumin (BSA) assessed the binding affinity of the compounds toward various drugs. Furthermore, DFT studies explored theoretical spectral calculations, energy differences, and electron cloud density. Notably, among the derivatives, the fluorophore 4-(7-amino-9-phenyl-1,2,3,4-tetrahydroacridin-2-yl)cyclohexan-1-one (**3e**) can specifically detect 2,4,6-trinitrophenol (Picric acid, PA), a fatal nitro explosive. Photophysical studies confirmed **3e**'s ability as a “turn-off” fluorescence chemosensor for PA with a detection limit of 1.766 × 10^–9^ M and a binding stoichiometric ratio of 1:1 between the probe and analyte. Structural confirmation of the probe was achieved through single-crystal XRD.

**Supplementary Information:**

The online version contains supplementary material available at 10.1186/s13065-025-01452-y.

## Introduction

**Fig. 1:**
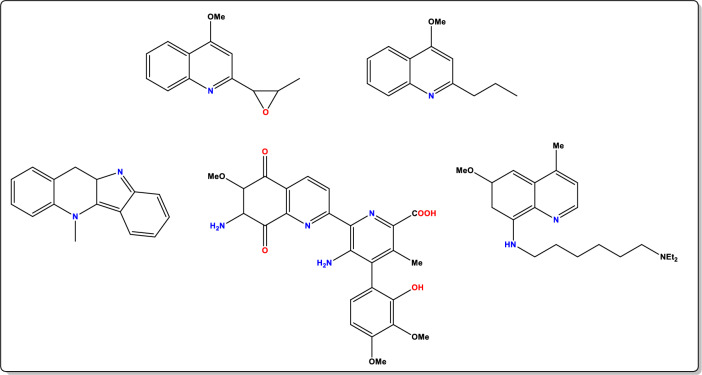
Quinoline derivatives from plant species

Neoteric solvents play a crucial role in synthesizing organic compounds in an eco-friendly manner. These solvents can be ionic liquids or modified versions of ionic liquids called Deep Eutectic Solvents (DESs) [1, 2]. DESs are formed by lowering the melting points of a hydrogen bond acceptor (HBA), such as a quaternary ammonium salt, and a hydrogen bond donor (HBD), like a carboxylic acid, by the charge delocalization that results from its hydrogen bond interaction. DESs were first coined by Abbot and his colleagues in 2003 [3] and have many properties like ionic liquids (ILs), such as low vapor pressure, a wide liquid range, non-flammability, ease of preparation, high tunability, and affordability. In addition, DESs are non-toxic, biodegradable, and reusable as well [4]. It is thus used as an excellent solvent in synthesizing different heterocyclic compounds. Quinoline is a class of heterocyclic organic compounds that have gained recognition in past years for their use in a variety of biological fields and synthetic organic chemistry. Its primary sources are coal processing, wood preservation, and petroleum. Initially, quinoline was first extracted from the coal tar by Friedland Ferdinand Runge in 1834. Additionally, diverse plant species can provide a variety of quinoline derivatives (Fig. 1), particularly from alkaloids [5]. These compounds have various applications, especially as anti-drug moieties due to the presence of nitrogen and oxygen, which make them biologically active [6]. Numerous synthesis methods have been reported for its synthesis, including conventional techniques, ultrasonic methods, and microwave (MW) irradiation approaches. These methods can be performed with or without the use of catalysts, but they face difficulties due to volatile solvents, high temperatures, expensive metal catalysts, and multi-step reactions, thus DESs are suited for green synthesis. Recent findings on the high-yield synthesis of different heterocycles employing DESs led us to further our interest in green methods. [7, 8]. Quinolines also demonstrate its importance in the newly developing field of chemosensors which are moieties where its, fluorescence, color, or conformational behaviour can be changed in the presence of specific analytes. These sensors are crucial for environmental monitoring and offer advantages like easy synthesis, low cost, and real-time detection via fluorescence ‘on–off’ switching [9].

**Scheme 1:**
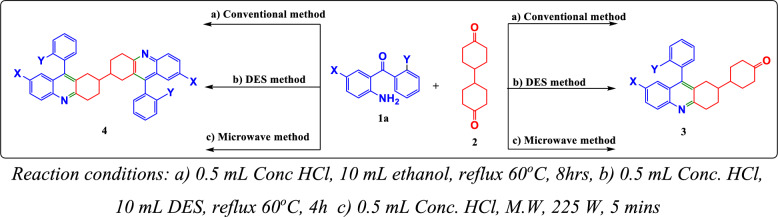
Synthesis of 4-(9-aryl-1,2,3,4-tetrahydroacridin-2-yl)cyclohexan-1-ones and 9,9′-diphenyl-1,1′,2,2′,3,3′,4,4′-octahydro-2,2′-biacridine via three methods

In continuing our fascination concerning the quinoline moiety, herewith we describe a simpler way of preparing quinolines by the Frienlander reaction [10, 11] which comprises a one-pot, two-component reaction of 2-amino-5-chlorobenzophenone and bicyclohexanone utilizing conventional, deep eutectic solvent, and microwave procedures (Scheme 1) with excellent yields [12–14]. MW-assisted organic synthesis addresses the chemical reactions in a shorter reaction time, solventless to synthesize the compounds in purity with less heating than the heat produced by conventional thermal reactions. MW radiations absorb and transmit heat waves differently, i.e., by attaining efficient internal heating via direct heat onto the bulk reaction mixture. Due to these advantages, it has become a significant alternative to synthetic chemicals and provides a low environmental impact [15].

We need effective methods to detect cations, anions, and nitro explosives that can cause significant harm to safeguard our environment. Chemosensors, among other types of sensors, can identify these substances quickly and affordably. Among the nitro explosives, picric acid (PA, or 2,4,6-trinitrophenol) is particularly concerning because of its high solubility in water. PA is commonly used in the leather, dye, and pesticide industries, resulting in dangerous levels of this pollutant being discharged into water bodies, which poses serious risks to both the environment and living organisms.

Consequently, researchers face challenges in developing rapid and straightforward methods for specifically identifying these pollutants. Fluorescent chemosensors are preferred over traditional detection methods due to their quick response times and significant fluorescence changes. The synthesized quinoline fluorophore, 4-(7-amino-9-phenyl-1,2,3,4-tetrahydroacridin-2-yl) cyclohexan-1-one, serves as an excellent fluorescent chemosensor that can be effectively “turned off” in the presence of PA. The fundamental principle behind this detection method is that the analyte PA quenches the fluorescence of the synthesized chemosensors. Photophysical studies have confirmed this effect by determining the detection limit for PA, which, according to the World Health Organization (WHO), should be less than 0.001 mg/L in groundwater [16]. Therefore, it is essential to develop effective chemosensors that can quickly provide data on the presence of these pollutants.

The synthesized compounds are screened through Density Functional Theory (DFT), which can predict various molecular characteristics such as molecular weight, absorption spectra, electron density around the molecules, and other chemical parameters. [17].

Quinoline moieties are well-known for their strong binding affinities with various serum albumins, which are the most abundant proteins in plasma. These proteins play a crucial role as the primary transporters of molecules in the circulatory systems of all vertebrates, including Human Serum Albumin (HSA) and animal serum albumin such as Bovine Serum Albumin (BSA). Both BSA and HSA exhibit structural similarities, consisting of 583 amino acid residues linked in a single chain. Their three-dimensional (3D) structure is characterized by three homologous domains (I, II, and III) arranged in a heart-shaped helical configuration, sharing approximately 76% identity [18, 19].

BSA is extensively studied for its interactions with various ligands, including drugs. Molecular docking studies reveal the binding domains and identify the most favorable sites for drug interaction within the protein structure. These studies validate the binding affinity of synthesized compounds with the well-researched protein BSA, using Autodock Vina 4.2 software [20] thus helps in assessing the potential biological applications of the compounds and laying the groundwork for further studies.

## Experimental

### Materials

All chemicals used in this study were obtained from Sigma Aldrich and Avra Chemicals in their original condition. Thin-layer chromatography (TLC) was employed to monitor all reactions. For column chromatography, silica gel (60–100 mesh, Avra Chemicals) was utilized with increasing percentages of ethyl acetate in hexane. The visualization of spots on TLC plates was performed through UV illumination and exposure to iodine vapor. Uncorrected melting points (mp) were determined using open-ended capillary tubes in an Elchem microprocessor-based DT apparatus. FT-IR spectra were recorded as KBr pellets using a Nicolet 6700 spectrometer. Microwave-assisted syntheses were also conducted in NuWav-PRO Microwave-Ultraviolet-Ultrasonic Synthesis/Extraction System. ^1^H-NMR at 400 MHz and ^13^C-NMR at 100 MHz were recorded for solutions in CDCl_3_ on a Bruker 400 spectrometer, using TMS as an internal reference. Chemical shift values are reported in parts per million (δ, ppm). High-resolution mass spectra were recorded using the Bruker MaXis HR-MS (ESI-Q-TOF–MS) instrument. Acetonitrile of HPLC grade was utilized for analytical studies. Absorption measurements were performed using a Jasco V-670 spectrometer at room temperature. Fluorescence emission spectra were recorded on a Hitachi F-7000 fluorescence spectrometer, also at room temperature, with a slit width of 5 nm for both excitation and emission. Single crystal X-ray diffraction (XRD) was conducted using a Bruker D8 Venture SC-XRD with Mo-radiation at 100 K.

### Synthesis

General procedure for the synthesis of 4-(9-aryl-1,2,3,4-tetrahydroacridin-2-yl)cyclohexan-1-ones (**3a**-**3d**) and its dimers 9,9′-diaryl-1,1′,2,2′,3,3′,4,4′-octahydro-2,2'-biacridine (**4a**-**4d**).

#### Conventional method

A mixture of 2-aminobenzophenone **1a** (0.5 g, 0.0025 mol) and 4,4′-bicyclohexanedione **2** (0.49 g, 0.0025 mol) in the presence of 0.5 mL of con. HCl and 10 mL of ethanol as solvent were refluxed for 8 h to obtain a white precipitate poured into ice-cold water, basified with 40% NaOH, filtered, dried, and purified using column chromatography (Scheme 1). Completion of the reaction was confirmed using TLC (40% ethyl acetate-hexane) and further characterized using techniques such as NMR, FTIR, and HRMS. As previously mentioned, the dimer forms of compounds **3a** to **3d** to obtain products **4a** to **4d** were prepared by reacting two equivalents of 2-aminobenzophenone **1a** (2.03 g, 0.010 mol) and one equivalent of 4,4′-bicyclohexanedione (1 g, 0.0051 mol) (Scheme 1).

#### Deep eutectic solvents method

2-amino benzophenone (0.5 g, 0.0025 mol) was treated with 4,4′-bicyclohexanedione (0.49 g, 0.0025 mol) in the presence of DESs (Supporting information, S25) as a solvent and con. HCl as a catalyst for 4 h in an oil bath at 60 °C. The clear yellow mixture affords a creamy white solid upon adding ice-cold water. The obtained precipitate is filtered, dried, and purified using column chromatography. Completion of the reaction was confirmed using TLC (40% ethyl acetate-hexane). The filtrate which contains DES has been retained by evaporating the water further and can be reused. As explained before the same procedure has been followed to prepare the dimer formations of derivatives **3a-3d** to obtain the products **4a-4d** by reacting 2 equivalents of 2-amino benzophenone **1a** (2.03 g, 0.010 mol) and 1 equivalent of 4,4′-bicyclohexanedione (1 g, 0.0051 mol) (Scheme 1).

##### General procedure for the synthesis of DESs

The required DESs are synthesized by stirring a mixture of a hydrogen bonding donor (HBD) and a hydrogen bond acceptor (HBA) at 60 °C in an oil bath until a homogeneous liquid is formed. The structures of choline chloride (HBD) and various HBAs, along with their equivalent molar ratios, are provided in the supplementary data [21–23]. Among these combinations, the choline chloride-ethylene glycol mixture yields the best results.

#### Microwave assisted method

Microwave assisted synthesis of 2-amino benzophenone (0.5 g, 0.0025 mol) with 4,4′-bicyclohexanedione (0.49 g, 0.0025 mol) in the presence of con. HCl as catalyst without the addition of any solvent at 250 W produced creamy white solution in 5 min of the reaction, further on the addition of ice-cold water gives a creamy white solid. The obtained solid is filtered, dried, and purified using column chromatography (Scheme 1). Completion of the reaction was confirmed using TLC (40% ethyl acetate-hexane). As already explained, a similar procedure has been followed to prepare the dimer formations of derivatives **3a-3d** to obtain the products **4a-4d**.

### General procedure of reduction of nitro derivative 4-(7-nitro-9-phenyl-1,2,3,4-tetrahydroacridin-2-yl)cyclohexan-1-one (**3d**) to 4-(7-amino-9-phenyl-1,2,3,4-tetrahydroacridin-2-yl)cyclohexan-1-one (**3e**)

**Scheme 2:**
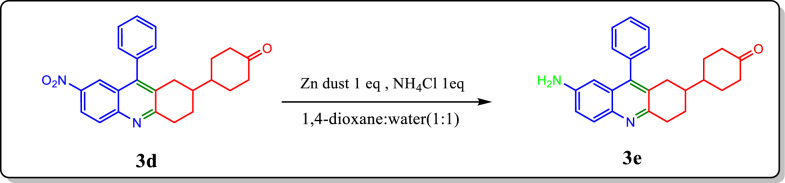
Synthesis of 4-(7-amino-9-phenyl-1,2,3,4-tetrahydroacridin-2-yl)cyclohexan-1-one (**3e**)

**Fig. 2:**
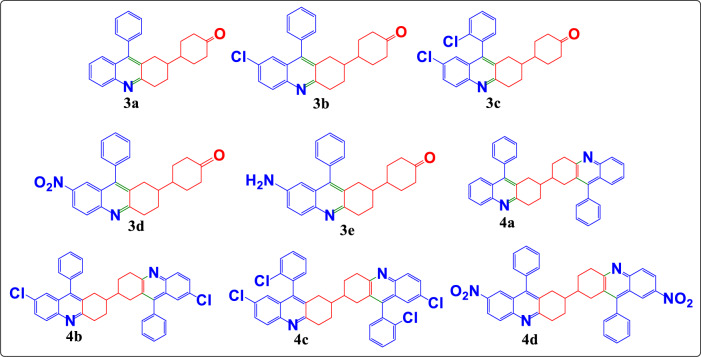
Quinoline-based acridine derivatives **3a-3e** and **4a-4d**

4-(7-nitro-9-phenyl-1,2,3,4-tetrahydroacridin-2-yl)cyclohexan-1-one (**3d**) was taken in a round bottom flask, to which Zn dust (1 eq), and ammonium chloride (1 eq) were added in the presence of mixture of 1,4-dioxane and water (1:1). The reaction mixture was stirred at room temperature for 4 h. The completion of reaction was confirmed by TLC (40% ethyl acetate-hexane). Further, the solution was filtered and into the filtrate, ethyl acetate was added. The organic layer was separated and rotor evaporated, followed purification by column chromatography in 40% ethyl acetate-hexane to get the desired product in a good yield (Scheme 2 ). All the newly synthesised compounds **3a-3e** and **4a-4d** are given as Fig. 2.

### Spectral characterisation

Data of characterization using FTIR, ^1^H-NMR, ^13^C-NMR, and HRMS (ESI) are as follows; the copies of all these spectra are included as Figs. S1-S46 in the supplementary data along with the crystal data of **3e**.

#### Spectral characterization of 3a (*4-(9-phenyl-1,2,3,4-tetrahydroacridin-2-yl)cyclohexan-1-one*)

White solid, yield 56%, m.p: 135 °C; FT-IR (υ/cm^−1^): 2947–2860 (C-H stretching), 1709 (C = O), 1483 (C-N stretching) Fig. S1.^1^H NMR (400 MHz, CDCl_3_):* δ* ppm 8.14 (*d, J* = 8.40 Hz, 1H), 7.64 (*t, J* = 6.04 Hz, 1H), 7.57–7.48 (*m*, 3H), 7.38–7.30 (*m*, 2H), 7.24–7.12 (*m*, 2H), 3.47–3.33 (*m*, 1H), 3.26–3.09 (*m*, 1H), 2.77–2.67 (*m*, 1H), 2.46–2.22 (*m*, 4H), 2.16–2.07 (*m*, 2H), 2.03–1.90 (*m*, 1H) Fig. S2; ^13^C NMR (100 MHz, CDCl_3_): *δ* ppm 210.77, 157.20, 135.43, 128.23, 127.85, 127.82, 127.78, 127.15, 126.76, 126.34, 125.68, 125.01, 124.92, 57.38, 39.82, 39.22, 37.37, 37.24, 30.79, 30.47, 28.83, 28.38, 24.85 Fig. S4; HRMS- ESI (m/z) calcd for C_25_ H_25_ NO [M + H]^+^  = 356.2014, found = 356.2014 Fig. S5.

#### Spectral characterization of 3b *(4-(7-chloro-9-phenyl-1,2,3,4-tetrahydroacridin-2-yl)cyclohexan-1-one)*

White solid, yield 63.19%, m.p:210 °C; FT-IR (υ/cm^−1^): 2947–2860 (C-H stretching),1706 (C = O),1427 (C-N stretching), 824 (aromatic ring peak), 697 (C–Cl stretching) Fig. S6; ^1^H NMR (400 MHz, CDCl_3_): *δ* ppm 7.92 (*d, J* = 8.80 Hz, 1H), 7.52 (*d, J* = 10.80 Hz, 4H), 7.23–7.14 (*m*, 3H), 3.30–3.28 (*m*, 3H), 2.72 (*d, J* = 16.80 Hz, 1H), 2.56 (*d, J* = 15.60 Hz, 1H), 2.34 (*t, J* = 8.40 Hz, 2H), 2.15 (*s*, 1H), 1.98 (*s*, 1H), 1.67 (*s*, 5H), 1.25 (*s*, 1H), 0.85 (*s*, 1H) Fig. S7; ^13^C NMR (100 MHz, CDCl_3_): *δ* ppm 210.77, 157.20, 135.43, 128.23, 127.85, 127.82, 127.78, 127.15, 126.76, 126.34, 125.68, 125.01, 124.92, 57.38, 39.82, 39.22, 37.37, 37.24, 30.79, 30.47, 28.83, 28.38, 24.85 Fig. S9; HRMS-ESI (m/z) calcd for C_25_H_24_ClNO [M + H]^+^  = 390.1610, found = 390.1625 Fig. S10.

#### Spectral characterization of 3c (*4-(7-chloro-9-(2-chlorophenyl)-1,2,3,4-tetrahydroacridin-2-yl)cyclohexan-1-one)*

White solid, yield 59.5%, m.p:118 °C; FT-IR (υ/cm^−1^): 2938–2854 (C-H stretching),1708 (C = O),1468 (C-N stretching),824 (aromatic ring peak), 755 (C–Cl stretching) Fig. S11; ^1^H NMR (400 MHz, CDCl_3_): *δ* ppm 7.95 (*dd, J* = 8.88, 14.42 Hz, 1H), 7.62–7.43 (*m*, 4H), 7.18–7.07 (*m*, 2H), 3.34–3.10 (*m*, 2H), 2.58 (*d, J* = 16.96 Hz, 1H), 2.48–2.27 (*m*, 5H), 2.14–2.04 (*m*, 2H), 1.98–1.93 (*m*, 1H), 1.75–1.67 (*m*, 3H), 1.51–1.44 (*m*, 1H), 1.30–1.24 (*m*, 1H) Fig. S12; ^13^C NMR (100 MHz, CDCl_3_): *δ* ppm 211.61, 163.05, 148.78, 148.37, 145.01, 135.14, 130.14, 129.95, 129.24, 128.87, 128.75, 125.70, 123.00, 122.22, 40.80, 40.29, 38.33, 34.28, 32.03, 29.86, 29.32, 25.82 Fig. S14; HRMS-ESI (m/z) calcd for C_25_ H_23_ Cl_2_NO [M + H]^+^  = 424.1235, found = 424.1235 Fig. S15.

#### Spectral characterization of 3d *(4-(7-nitro-9-phenyl-1,2,3,4-tetrahydroacridin-2-yl)cyclohexan-1-one)*

Yellowish orange solid, yield 60.5%, m.pt:220 °C; FT-IR (υ/cm^−1^): 2932,2876 (C-H stretching), 1970 (C = O), 1524, 1333 (NO_2_ stretching), 1484 (C-N stretching), 855 (aromatic ring peak) Fig. S16; ^1^H NMR (400 MHz, CDCl_3_): *δ* ppm 8.36 (*dd, J* = 2.48, 9.20 Hz, 1H), 8.27 (*d, J* = 2.36 Hz, 4H), 8.11 (*d, J* = 9.16 Hz, 1H), 7.6l0-7.57 (*m*, 3H), 7.23 (*t, J* = 1.48 Hz, 2H), 3.41–3.34 (*m*, 1H), 3.24–3.16 (*m*, 1H), 2.75 (*d, J* = 2.44 Hz, 1H), 2.49–2.27 (*m*, 5H), 2.19–1.90 (*m*, 3H), 1.73–1.67 (*m*, 3H), 1.53–1.41 (*m*, 2H) Fig. S17; ^13^C NMR (100 MHz, CDCl_3_): *δ* ppm 211.61, 163.05, 148.78, 148.37, 145.01, 135.14, 130.14, 129.95, 129.24, 128.87, 128.75, 125.70, 123.00, 122.22, 40.80, 40.29, 38.33, 34.28, 32.03, 29.86, 29.32, 25.82 Fig. S19; HRMS-ESI (m/z) calcd for C_25_H_24_N_2_O_3_ [M + H]^+^  = 401.1866, found = 401.1865 Fig. S20.

#### Spectral characterization of 3e *(4-(7-amino-9-phenyl-1,2,3,4-tetrahydroacridin-2-yl)cyclohexan-1-one)*

Brown solid, yield 93% m.pt: 230 °C; FT-IR (υ/cm^−1^): 3302 (-NH_2_ peak), 2929,2859 (C-H stretching), 1709 (C = O), 1495 (C-N stretching), 825 (aromatic ring peak) Fig. 14; 400 MHz, CDCl_3_: δ 7.92 (d, *J* = 8.80 Hz, 1H), 7.50–7.40 (m, 3H), 7.19 (t, *J* = 6.40 Hz, 2H), 7.08 (d, *J* = 8.60 Hz, 1H), 6.36 (s, 1H), 3.84 (s, 1H), 3.32 (d, *J* = 17.20 Hz, 1H), 3.16–3.14 (m, 1H), 2.61 (d, *J* = 16.40 Hz, 1H), 2.38–2.34 (m, 7H), 1.91–1.88 (m, 6H) Fig. S21; ^13^C NMR (100 MHz, CDCl_3_): *δ* ppm 211.78, 153.82, 146.16, 144.45, 139.87, 136.94, 128.89, 128.81, 128.19, 127.89, 121.52, 105.85, 40.82, 40.22, 38.40, 32.49, 31.81, 29.81, 29.68, 29.41, 25.95 Fig. S23; HRMS-ESI (m/z) calcd for C_25_H_26_N_2_O [M + H]^+^  = 371.2079, found = 371.2123 Fig. S26.

#### Spectral characterization of 4a *(9,9'-diphenyl-1,1',2,2',3,3',4,4'-octahydro-2,2'-biacridine*)

White solid, yield 38.7%, m.p: 230 °C; FT-IR (υ/cm^−1^): 2950–2867 (C-H stretching), 1492 (C-N stretching), 763 (aromatic ring peak) Fig. S27, ^1^H NMR (400 MHz, CDCl_3_): *δ* ppm 8.89 (*dd, J* = 8.40, 12.80 Hz, 2H), 7.88 (*t, J* = 7.20 Hz, 2H), 7.69–7.58 (*m*, 8H), 7.50 (*dd, J* = 8.00, 20.20 Hz, 2H), 7.22 (*q, J* = 4.80 Hz, 3H), 4.11 (*d, J* = 18.00 Hz, 1H), 3.96 (*d, J* = 19.20 Hz, 1H), 3.46 (*dd, J* = 9.20, 15.80 Hz, 2H), 2.85 (*d, J* = 16.80 Hz, 1H), 2.45 (*dd, J* = 8.00, 16.60 Hz, 2H), 2.04 (*d, J* = 12.00 Hz, 1H), 1.70 (*t, J* = 36.40 Hz, 6H) Fig. S28; ^13^C NMR (100 MHz, CDCl_3_): *δ* ppm 135.66, 129.03, 128.96, 128.67, 128.58, 128.46, 128.12, 126.81, 126.16, 37.99, 31.74, 31.40 Fig. S30, HRMS: ESI (m/z) calcd for C_38_ H_32_ N_2_ [M + H]^+^  = 517.2630, found = 517.2644 Fig. S31.

#### Spectral characterization of 4b (*7,7′-dichloro-9,9′-diphenyl-1,1′,2,2′,3,3′,4,4′-octahydro-2,2′-biacridine*)

White solid, yield 33.25%, m.p:240 °C; FT-IR (υ/cm^−1^): 3060, 2923, 2861(C-H stretching), 1677 (C = O), 1563, 1472 (C-N stretching), 829 (aromatic ring peak), 692 (C–Cl stretching) Fig. S32; ^1^H NMR (400 MHz, CDCl_3_): *δ* ppm 7.92 (*d, J* = 8.80 Hz, 2H), 7.58–7.49 (*m*, 9H), 7.23–7.09 (*m*, 5H), 3.30 3.21 (m, 1H), 3.14–3.01 (*m*, 1H), 2.71 (*d, J* = 14.80 Hz, 1H), 2.56 (*d, J* = 17.60 Hz, 1H), 2.34 (*dd, J* = 6.80, 13.60 Hz, 2H), 2.17 (*t, J* = 4.40 Hz, 1H), 1.99 (*s*, 4H) Fig. S33, ^13^C NMR (100 MHz, CDCl_3_): *δ* ppm 159.08, 146.06 144.72, 136.02, 131.26; 130.03, 129.42 128.92, 128.88, 128.85, 128.7, 128.55, 128.17, 127.35, 124.57, 38.32, 33.83, 32.06, 25.52 Fig. S35, HRMS: ESI (m/z) calcd for C_38_H_30_Cl_2_N_2_ [M + H]^+^  = 585.1867, found = 585.1864 Fig. S36.

#### Spectral characterization of 4c (*7,7′-dichloro-9,9′-bis(2-chlorophenyl)-1,1′,2,2′,3,3′,4,4′-octahydro-2,2′-biacridine*)

White solid, yield 29.7%, m.p: 200 °C; FT-IR (υ/cm^−1^): 2929,2866 (C-H stretching), 1580, 1483 (C-N stretching), 834 (aromatic ring peak), 755 (C–Cl stretching) Fig. S37; ^1^H NMR (400 MHz, CDCl_3_): *δ* ppm 7.95 (*d, J* = 9.20 Hz, 2H), 7.59–7.57 (*m*, 8H), 7.11–7.09 (m*,* 4H), 3.34–3.33 (*m*, 2H), 3.18–3.17 (*m*, 2H), 2.51–2.47 (*m*, 4H), 2.17 (*d, J* = 4.80 Hz, 2H), 1.73 (*d, J* = 6.00 Hz, 2H), 1.25 (*s*, 2H) Fig. S38; ^13^C NMR (100 MHz, CDCl_3_): *δ* ppm 159.09, 144.70, 143.27, 134.94, 133.20, 132.88, 131.65, 130.43, 130.18, 129.87, 129.19, 127.42, 127.34, 123.83, 38.31, 37.69, 33.74, 25.29 Fig. S40, HRMS: ESI (m/z) calcd for C_38_H_28_Cl_4_N_2_ [M-H]^+^  = 653.1075, found = 653.1085 Fig. S41.

#### Spectral characterization of 4d (*7,7′-dinitro-9,9′-diphenyl-1,1′,2,2′,3,3′,4,4′-octahydro-2,2′-biacridine*)

Yellowish orange solid, yield 24.9%, m.pt:198 °C; FT-IR (υ/cm^−1^): 3185, 2929 (C-H stretching), 1580, 1523 (NO_2_ stretching), 1489 (C-N stretching), 834 (aromatic ring peak) Fig. S42; ^1^H NMR (400 MHz, CDCl_3_): δ ppm 8.36 (dd, *J* = 2.40, 9.40 Hz, 2H), 8.25 (dd, *J* = 2.40, 11.60 Hz, 2H), 8.09 (d, *J* = 9.20 Hz, 1H), 7.66–7.60 (m, 6H), 7.23–7.21 (m, 1H), 3.40–3.26 (m, 2H), 3.21–3.08 (m, 2H), 2.43–2.42 (m, 2H), 2.22 (t, *J* = 4.80 Hz, 1H), 2.04 (s, 1H), 1.69 (t, *J* = 7.20 Hz, 4H), 1.26 (t, *J* = 7.20 Hz, 1H) Fig. S43; ^13^C NMR (100 MHz, CDCl_3_): δ ppm 162.96, 148.34, 145.02, 135.01, 130.14, 129.17, 128.88, 128.81, 128.76, 125.67, 122.97, 122.14, 38.29, 38.20, 25.34 Fig. S45, HRMS: ESI (m/z) calcd for C_38_H_30_N_4_O_4_ [M + H]^+^  = 607.2344, found = 607.2345 Fig. S46.

## Results and discussion

### Chemistry

**Fig. 3:**
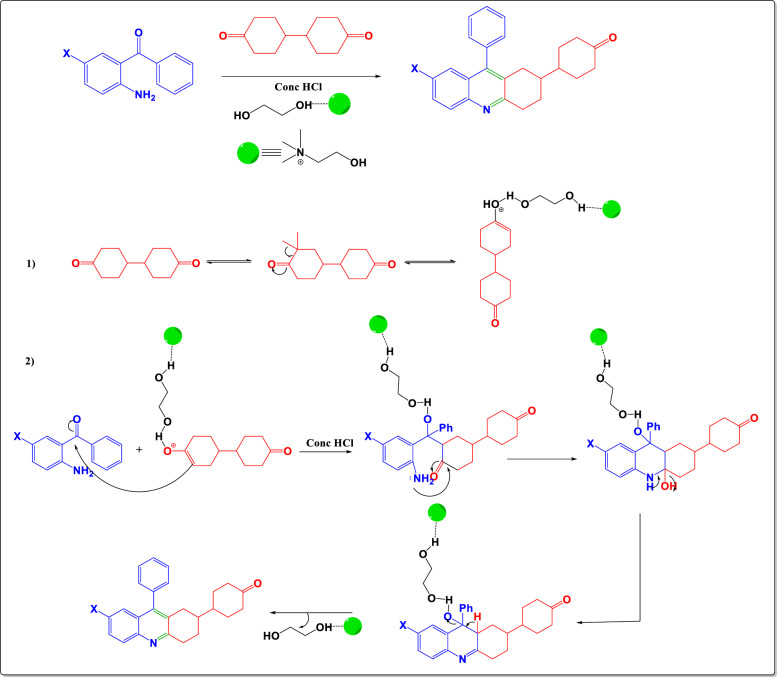
Proposed mechanism of the synthesis

The target molecules, 4-(9-aryl-1,2,3,4-tetrahydroacridin-2-yl)cyclohexan-1-ones (**3a-3d**), were synthesized using a conventional approach. This method involved refluxing a mixture of the corresponding 2-amino benzophenone (**1**) and 4,4′-bicyclohexanedione (**2**) in the presence of concentrated hydrochloric acid in ethanol for 8 h. This process yielded a white solid, which was then poured into ice-cold water. The mixture was basified with 40% sodium hydroxide, filtered, dried, and purified using column chromatography (see Scheme 1). The completeness of the reaction was confirmed by thin-layer chromatography (TLC) using a solvent mixture of 40% ethyl acetate and hexane, and the products were characterized using techniques such as FTIR, NMR, and HRMS. The reaction conditions were optimized by varying the solvents, including acetonitrile (75 °C, yield: 33.7%), ethanol (60 °C, yield: 56%), methanol (55 °C, yield: 53%), DMF (145 °C, yield: 11%), and DMSO (170 °C, yield: 22%), with ethanol providing the highest yield. Similarly, the 9,9′-diaryl-1,1′,2,2′,3,3′,4,4′-octahydro-2,2′-bisacridines (**4a-4d**), which are dimer forms of 3a-3d, were synthesized using the same procedure. In this case, 2 equivalents of the corresponding 2-aminobenzophenones (**1**) were used (2.03 g, 0.010 mol) along with 1 equivalent of 4,4′-bicyclohexanedione (**2**) (1 g, 0.0051 mol) as outlined in Scheme 1. The target molecules (**3a**-**3d** and **4a**-**4d**) were also synthesized under microwave irradiation in solvent-free conditions, resulting in successful reactions. To optimize the microwave conditions, the power level was varied, and it was found that 30% (225 W) of an 800 W microwave yielded the best results. Recently, Deep Eutectic Solvents (DES) have gained attention as emerging green solvents due to their lower vapour pressure, biodegradability, and reusability. Therefore, the synthesis was also attempted using DES. Various combinations of DES were tested, incorporating choline chloride (ChCl) HBA and malonic acid, oxalic acid, urea, and ethylene glycol as HBD, as summarized in the supporting information (S25, Table S1). Among these, the combination of ChCl and ethylene glycol as DES yielded the best results. A comparison of the results obtained using various DESs is provided in Table 1, and the proposed mechanism of the reaction via DES is depicted in Fig. 3. The microwave-assisted method was superior for synthesizing compounds **3a-3d**, while the DES method was more effective for synthesizing their dimeric forms **4a-4d** (see Table 2).

**Table 1:** Comparative yield percentages using various DESs

S. No	Products	T(^o^C)	Time (h)	Yield (%) using various DESs			
				ChCl-Malonic acid (1:1)	ChCl-Oxalic acid (1:1)	ChCl—Urea (1:2)	ChCl- Ethylene glycol (1:2)
1	**3a**	60	4	27.7	16.6	22.19	53.8
2	**3b**	60	4	23.8	11.9	14.2	49.5
3	**3c**	60	4	9.4	7.5	6.2	37.7
4	**3d**	60	4	33.2	15.13	9.6	50.9
5	**4a**	60	4	9.8	3.0	2.5	37.6
6	**4b**	60	4	20.6	18.6	13.3	40.0
7	**4c**	60	4	12.4	9.5	9.2	23.1
8	**4d**	60	4	8.1	6.8	6.0	26.2

**Table 2:** Comparative yield percentage of the synthesised derivatives via the three methods

S. No	Products	T (^o^C)	^a^Conventional method		^b^DEG as solvent		^c^Microwave method	
			Yield	Time (hrs)	Yield	Time (hrs)	Yield	Time (mins)
1	**3a**	60	56.0	8	53.8	4	61.0	5
2	**3b**	60	63.2	8	49.5	4	65.5	5
3	**3c**	60	59.5	8	37.7	4	50.3	5
4	**3d**	60	60.5	8	50.9	4	30.0	5
5	**4a**	60	38.7	8	37.6	4	25.0	5
6	**4b**	60	33.2	8	40.0	4	30.0	5
7	**4c**	60	29.7	8	23.1	4	12.0	5
8	**4d**	60	24.9	8	26.2	4	19.2	5

Solvent ^a^ethanol, ^b^DEG- ChCl-Ethylene Glycol, ^c^solventless-225W

All newly synthesized compounds (**3a**-**3d** and **4a**-**4d**) were thoroughly characterized using FT-IR, ^1^H NMR, ^13^C NMR, and HR-MS (ESI) spectral data, which are included in the experimental section and the supplementary file. The synthesized compounds were also subjected to Density Functional Theory (DFT) and Time dependent-DFT (TD-DFT) studies conducted to investigate their geometry, electrostatic potential mapping (ESP), and electronic transition states. Additionally, in silico studies with BSA were conducted to evaluate the binding energy and binding capability of the compounds. This information will aid in identifying potential applications of the synthesized compounds in drug discovery in the future.

### Recyclability of DES

**Fig. 4:**
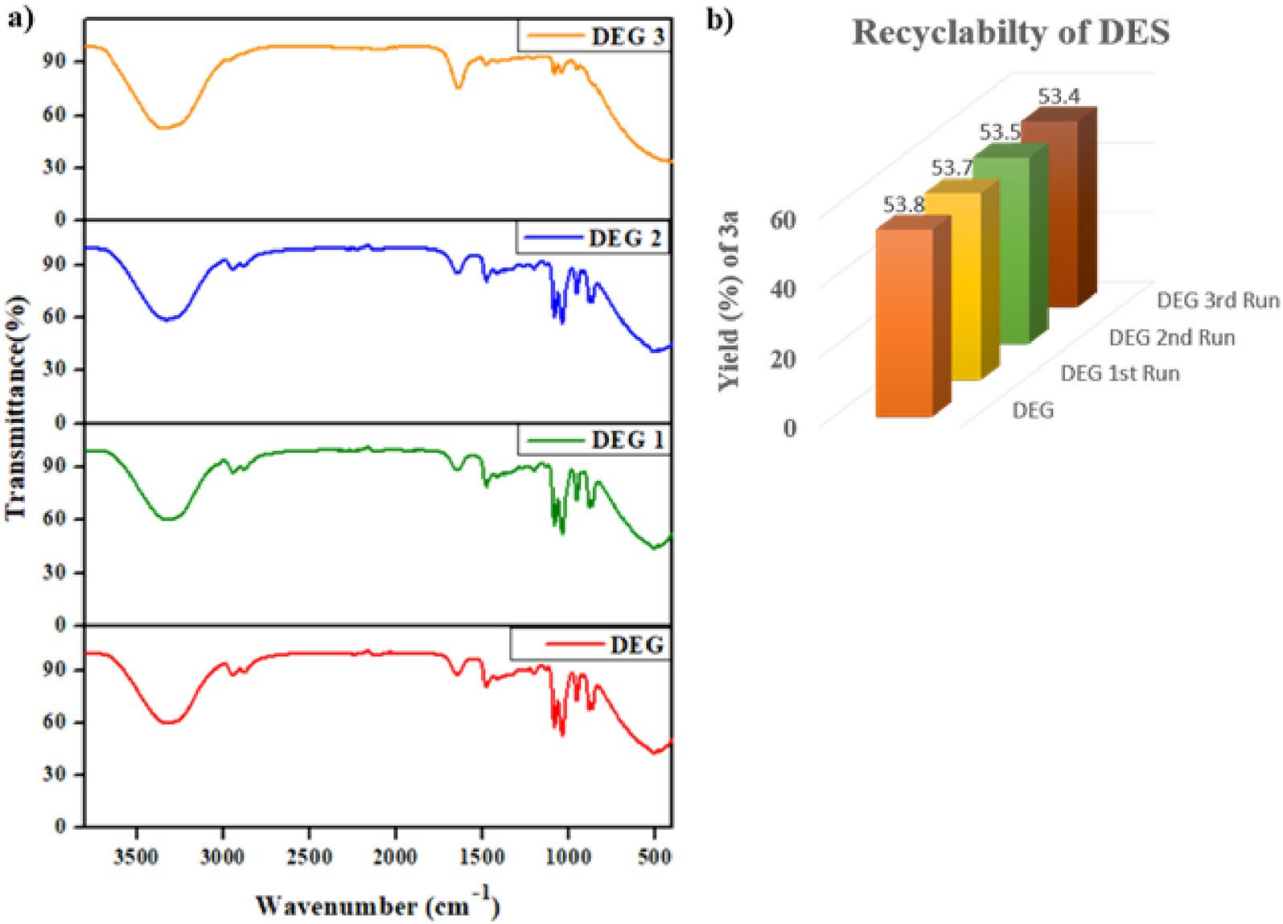
**a** FTIR spectra of DES (ChCl-Ethylene Glycol) after each reaction. **b** Bar graph representing recyclability of DES with yield percentage of **3a**

The recyclability of solvents is an important factor in organic reactions as it reduces solvent wastage. DES has the advantage of being recyclable [24]. After the reaction, the product is filtered and washed with cold water. The filtrate, containing DES, is then evaporated to retrieve the DES for the next run. Figure 4a shows that the FT-IR spectra for the DES, can be reused up to four times. However, after four uses, the recycled DES does not support the reaction because the reactants are not homogeneous.

### Calculation of green metrics

Green metrics calculation helps to know the efficiency of the newly synthesized compounds towards green chemistry. It aids in reducing the usage of toxic chemicals thus generating minimal hazardous waste. Therefore, it is significant in evaluating the greenness in chemical reactions and bringing down the impact of hazardousness on the environment and surroundings [25, 26]**.** Following are the methods used to calculate green metrics, and the values are tabulated in Table 3. The value of CE and E-factor shows a good profile in this calculation.

**Table 3:** Green Metrics calculation of the synthesized compounds **3a**-**3d** and **4a**-**4d**

Entry	Compound	Molecular formula	Yield (%)	Calculated green chemistry matrices				
				AE	PMI	CE	RME	E-factor
1	**3a**	C_25_ H_25_ NO	53.8	90.9	1.297	100	77.1	0.297
2	**3b**	C_25_H_24_ClNO	49.5	90.4	1.295	100	77.2	0.295
3	**3c**	C_25_ H_23_ Cl_2_NO	37.7	92.1	1.289	100	77.5	0.289
4	**3d**	C_25_ H_23_ Cl_2_NO	50.9	92.0	1.291	100	77.4	0.291
5	**4a**	C_38_ H_32_ N_2_	37.6	87.8	1.292	100	77.3	0.292
6	**4b**	C_38_H_30_Cl_2_N_2_	40.0	87.4	1.289	100	77.5	0.289
7	**4c**	C_38_H_28_Cl_4_N_2_	23.1	89.8	1.278	100	78.2	0.278
8	**4d**	C_38_H_30_N_4_O_4_	26.2	89.3	1.282	100	78.0	0.282

*AE* Atomic economy, *PMI* Process Mass Intensity, *CE* Carbon efficiency, *RME* Reaction mass efficiency

### DFT and TD-DFT studies

**Fig. 5:**
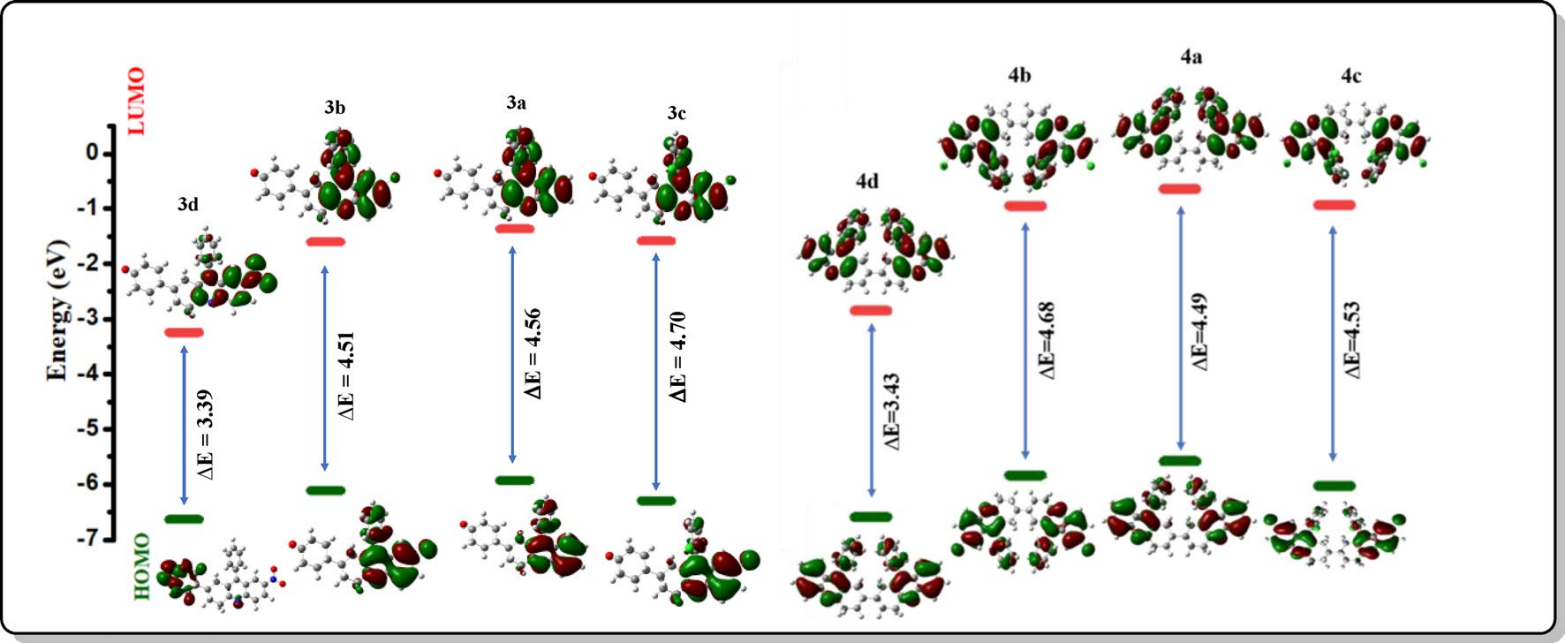
FMO diagrams of derivatives **3a-4d**

The structural optimization was optimized based on the density functional theory (DFT) and was carried out on the Gaussian 09 program with B3LYP functional and the 6-311G basis set of the Gaussian 09W program. The absorption spectra and electrostatic potential of the synthesized compounds were obtained by time-dependent density-functional theory (TD-DFT) computational simulations [27]. The frontier molecular orbital (FMO) diagrams of the synthesized compounds were calculated and shown in Fig. 5, from which the energy difference between the Highest Occupied Molecular Orbitals (HOMO) and Lowest Unoccupied Molecular Orbitals (LUMO) can be calculated and is tabulated in Table.S2 given in supporting information along with the dipole moment and molecular weight of each compound. FMO diagram shows that **3d** and **4d** acridines have the lowest energy band gap thereby proving their high absorbance in the UV–visible spectrum.

UV–vis absorption spectroscopy of the synthesized compounds was simulated and tested in chloroform using time-depending density functional theory (TD-DFT), B3LYP generalization, 6-311G basis group, and results are shown in supporting information (S27). The results obtained via theoretical calculation show the maximum absorption for **3d** and **4d** at 435 and 590 nm respectively. Thus, from the theoretical calculation, it can be proved that compounds **3d** and **4d** with the lowest obtained energy show the highest absorption wavelength. The selected transitions that exhibit the percentage of major contributions of intra-charge transfer (ICT) obtained were tabulated in supporting information (Table S3).

**Fig. 6:**
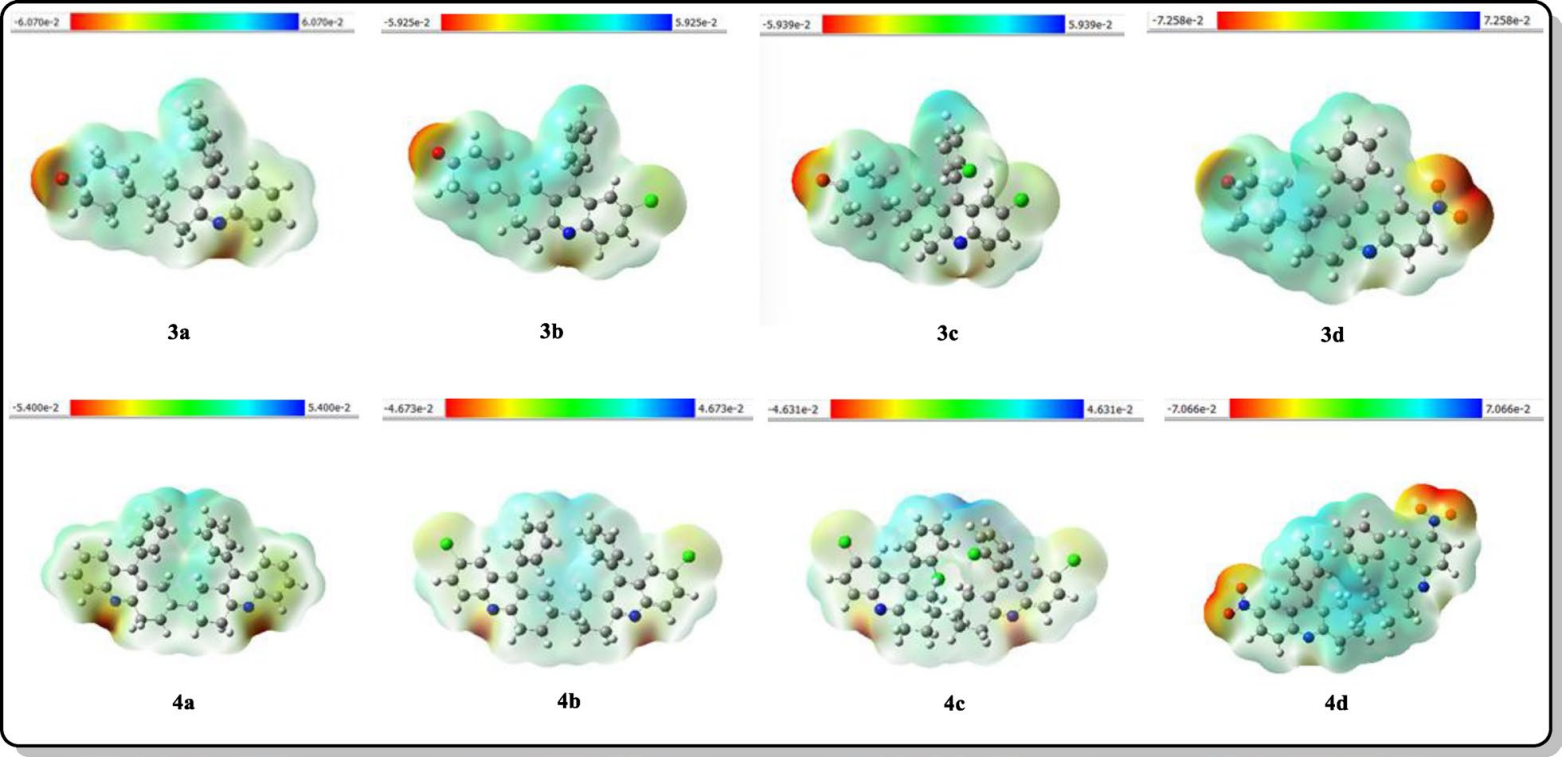
The total electron density surface of the synthesized compounds mapped with their electrostatic potential

Electrostatic potential (ESP) analysis is well known as it analyses the electron cloud density in real space [27]. Figure 6 shows the total density surface mapped with an electrostatic potential of synthesized compounds. The Reduced Density Gradient (RDG) method uses a color scale to represent the interactions between molecules. Red indicates strong attractions, such as hydrogen bonds and halogen bonds. Green represents van der Waals interactions, while blue signifies strong mutual repulsion, which can occur in cases of steric hindrance within rings and cages. As the scale moves from red to blue, the electron deficiency decreases [28] thus, ESP helps in knowing the absorption center's active position for further interactions of the synthesized compounds.

### Molecular docking

The binding ability of a drug compound was evaluated by studying its interactions with a model protein (BSA, PDB ID Code: 4F5S) using molecular docking [29, 30]. Autodock Vina was used for this purpose, and the Biovia Discovery Studio visualizer created 3D and 2D diagrams. The significant amino acids involved in the interactions were represented using circles. The designed molecules are shown in red. Various types of interactions were described using different colours and dotted lines. The orange colour dotted lines indicate the π-anion and cation interactions; light pink, dark pink and violet colour dotted lines represent π-alkyl, π-πT-shaped alkyl and π-sigma interactions respectively. Light blue dotted lines indicate hydrogen bonding with electronegative elements, such as nitrogen (N) and oxygen (O) atoms. Van der Waals interactions are represented by light green-colored amino acids, while red-colored amino acids indicate unfavorable interactions. The blue halo surrounding the interacting residues illustrates the solvent-accessible surface. The docking images can be found in the supporting information (Figs. S47–S55).

**Fig. 7:**
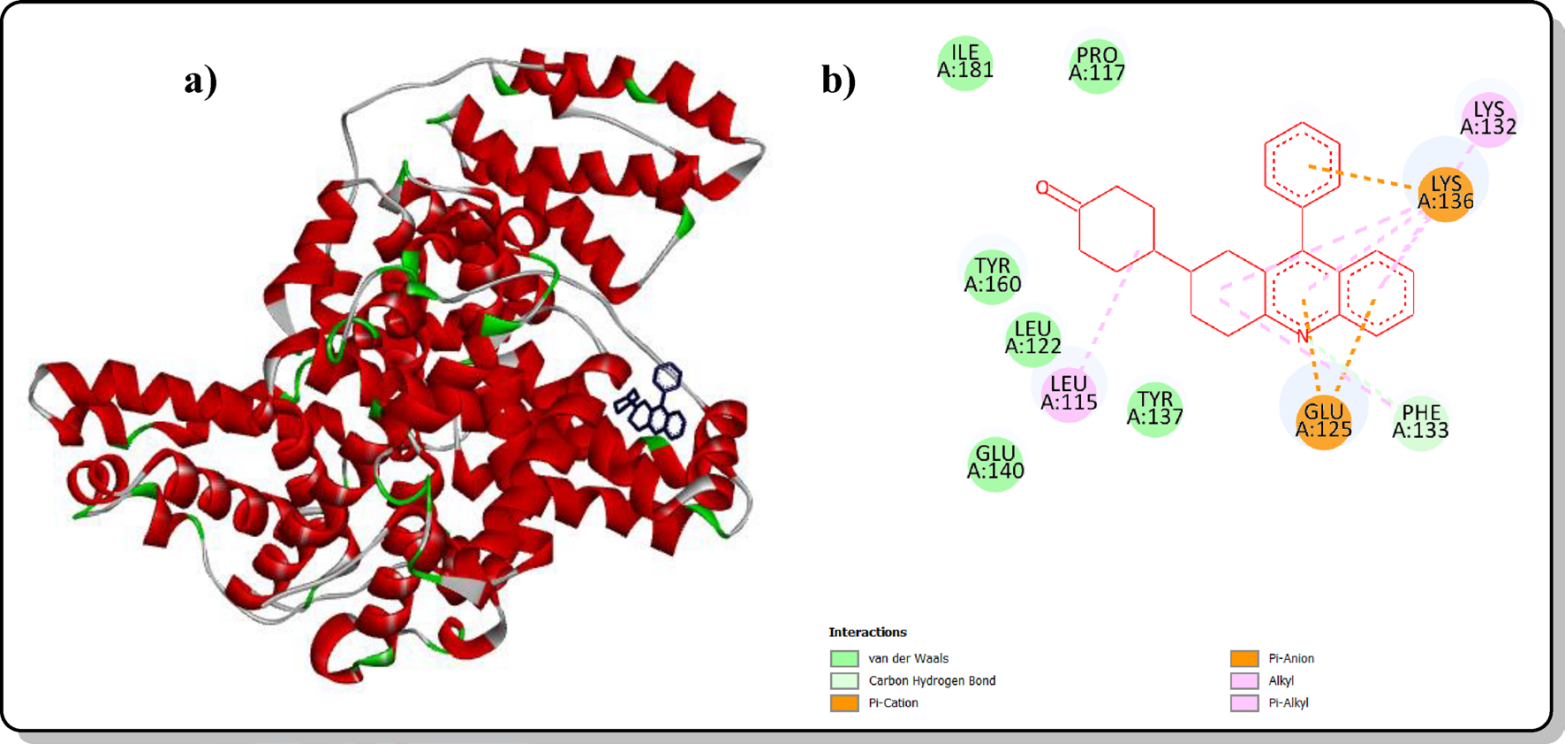
3D (**a**) and 2D (**b**) interactions of reference 4a with receptor (PDB ID CODE: 4F5S)

Compound **4a** was found to have a binding energy value of − 10.28 K cal and an inhibitory value of 28.96 (Table.S4), indicating its ease of formation of a complex with BSA. The compound interacts with the protein pocket through amino acids such as ARG, GLU, PRO, and ILE, which mainly bind to the binding sites IB and IIIA [16]. Therefore, the results suggest that compound **4a** has a high drug-binding ability with BSA and could be a promising candidate for various other antidrug purposes thus helping to carry out biological applications in the future. Figure 7 shows the 3D (Fig. 7a) and 2D (Fig. 7b) images of compound 4a interacting with BSA (PDB: 4F5S).

### Synthesis of 4-(7-amino-9-phenyl-1,2,3,4-tetrahydroacridin-2-yl)cyclohexan-1-one (3e) and its characterisation

Furthermore, the nitro group of compound **3d** was reduced to yield its corresponding amino derivative, 4-(7-amino-9-phenyl-1,2,3,4-tetrahydroacridin-2-yl)cyclohexan-1-one (**3e**). This compound is anticipated to serve as an excellent turn-off fluorescent sensor for the efficient detection of the lethal nitro explosive, picric acid. The reduction of compound **4d** was also conducted, revealing minimal detection capabilities for picric acid, as only one of its two nitro groups was reduced, which decreased its effectiveness as a chemosensor. Hence there occurs a push–pull mechanism within the reduced moiety which causes it to less contribute towards electron-deficient picric acid. This indicates a need for further modifications to enhance its detection capabilities.

**Fig. 8:**
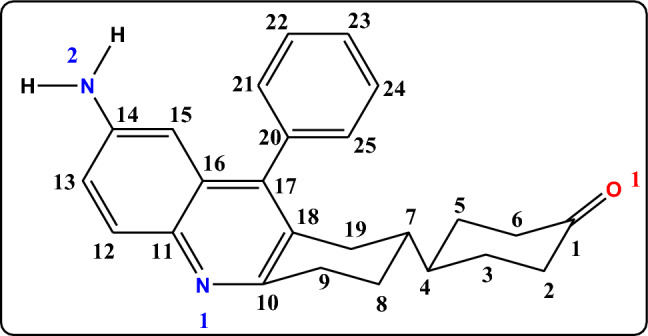
Structure of (R)-4-(7-amino-9-phenyl-1,2,3,4-tetrahydroacridin-2-yl)cyclohexan-1-one (**3e**) represented in chair form

The structure of compound **3e** has been thoroughly characterized using IR, ^1^H NMR, ^13^C NMR, and mass spectral data, along with single-crystal X-ray diffraction studies and its structure has been arrived (Fig. 8). The single-crystal X-ray diffraction studies revealed that the two cyclohexyl rings are available in the chair forms and both the rings are connected through the equatorial bond. The examination of the ^1^H NMR spectrum shows the two different sets of signals in aliphatic and aromatic zones distinctly, and the H, H-COSY (supplementary information Fig. S24) further reveals that there are no cross peaks observed between the aliphatic and aromatic signals. The ^1^H NMR spectrum exhibited the following signals in the aromatic region δ 7.92 (d, *J* = 8.60 Hz, 1H), 7.50–7.40 (m, 3H), 7.19 (t, *J* = 6.40 Hz, 2H), 7.08 (d, 8.6 Hz, 1H), 6.36 (s, 1H) ppm. The examination of these signals shows that the signal at δ 6.36 ppm is assigned to C-15 in the aromatic ring. The H,H-COSY spectrum reveals that the signal at δ 6.36 ppm coupled with the signal at δ 7.08 ppm. The signal at δ 7.08 ppm was found to couple with δ 7.92 and δ 6.36 ppm. Based on these observations the signal at δ 7.08 ppm is assigned to the proton at C-13 and the other signal at δ 7.92 ppm is assigned to C-12. The signal at δ 7.19 ppm integrating for two protons appeared as a triplet in ^1^H NMR found to have two coupling partners in H, H-COSY (but merged and appeared as multiplet in ^1^H NMR and integrating for three protons) assigned as *meta* protons at C-22 and C-24. The *ortho* and *para* protons merged and appeared as multiplet in the range of δ 7.50 –7.40 ppm. The signals at δ 2.07 ppm and δ 1.27 ppm integrating for one proton each are due to the protons present at C-4 and C-7 respectively. The protons of C-3, C-2, C-6, and C-5 are appeared as multiplets and resonating from δ 3.3 ppm to δ 2.24 ppm. The signals at δ 1.9 to δ 1.43 ppm appeared as multiplets due to the protons at C-8, C-9 and C-19. The ^13^C NMR and HSQC (supplementary information, Fig. S25) spectra of **3e** have been examined and the following observations made. The carbonyl carbon C-1 appears at δ 210 ppm. The signals at δ 40.82 and δ 40.23 are assigned to carbon C-7 and C-4 respectively using the HSQC spectrum. The signal at δ 153.82 ppm is assigned to C-10 and other non-bearing proton carbons at C-18, C-17, C-16, C-11, and C-20 resonates at δ 127.97, 136.94, 128.19, 144.45 and 139.87 ppm respectively. The carbon attached to the amine group resonates at δ 146.16 ppm and is assigned to C-14. The signals δ 127.89, 121.52, and 105.85 are assigned to C-12, C-13, and C-15 carbons. C-25 and C-21 carbons appear in same environment thus resonating at δ 128.81 ppm. The carbons C-24, C-22, and C-23 also appear in the same environment; the peaks appear at δ 128.89 ppm. The signals at δ 38.4, 32.49, and 31.81 ppm are assigned to C-3, C-2 and C-6 respectively. The signal at δ 29.81ppm is assigned to C-5 carbon bearing two protons. The signals at δ 29.68, 29.41, and 25.95 ppm are assigned as C-9, C-19, and C-8 carbons bearing two protons each.

### Single crystal X-ray diffraction studies

**Fig. 9:**
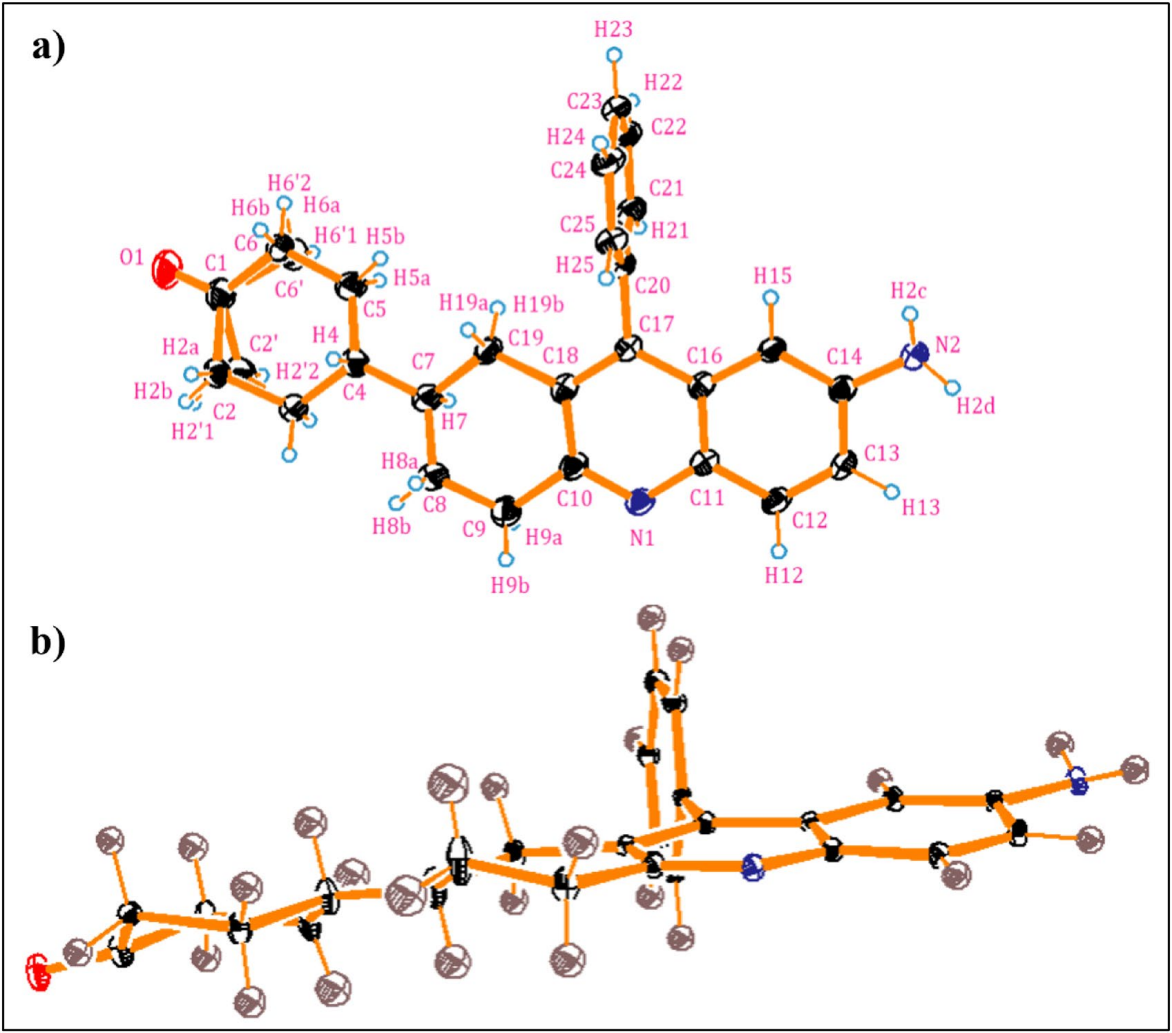
**a** ORTEP diagram of **3e** drawn at an ellipsoidal probability 30%; **b** ORTEP diagram with a chair form view of aliphatic rings

The single crystal X-ray diffraction (SC-XRD) studies on compound **3e** were performed on a brown needle-like crystal that was grown from a toluene-DMSO solvent mixture in a 1:1 ratio by slow evaporation. Data collection was conducted at a low temperature of 100 K using a Bruker D8 Venture SC-XRD with Mo-radiation. The data was processed using APEX3 software, and the structure was solved and refined using SHELXTL. The crystal structure has been deposited in the Cambridge Crystallographic Data Centre (CCDC) with the accession number 2404164. Supplementary information includes crystal data, structure refinement for **3e** (Table S5), as well as selected bond lengths, bond angles, and torsional angles (Table S6). The compound **3e** crystallized in an orthorhombic arrangement with a non-centrosymmetric space group designated as P_21_. The crystal dimensions were measured at 0.599 × 0.170 × 0.143 mm. The ORTEP diagram of **3e** (Fig. 9a) indicates that the C(1)-O(1) bond length is 1.19 Å, signifying a double bond. In contrast, the bond lengths for C(1)-C(6) and C(1)-C(6′) are measured at 1.51 Å, indicating they are single bonds. The C(4)-H(4) and C(7)-H(7) bond lengths were found to be 1.0 Å, which is typical for a C-H single bond. The bicyclohexane ring adopts a chair conformation, suggesting that the two rings are connected via an equatorial-equatorial bond (i.e., carbons C(4) and C(7)). Notably, carbon C(7) is chiral, indicating that its configuration is R (Fig. 9b). The C(10)-N(1) bond length measures 1.32 Å, indicating a double bond between carbon and nitrogen. In particular, carbon C(1) is sp^2^ hybridized, as evidenced by the bond angles between O(1)-C(1)-C(6′) and O(1)-C(1)-C(6) being 120.7° and 121.7°, respectively, suggesting a planar geometry. The bond angles for C(3)-C(2)-H(2a) and C(3)-C(2)-H(2b) are 109.1°, indicating that carbon is sp^3^ hybridized. Similarly, C(14)-N(2)-H(2c) and C(14)-N(2)-H(2d) have bond angles of 114° and 115°, implying sp^3^ hybridization for carbon, with a slight deviation from the ideal bond angle due to the presence of an active lone pair on the N(2) atom. The phenyl ring positioned above the quinoline ring is out of the plane; the torsional angles, C(18)-C(17)-C(20)-C(25) and C(16)-C(17)-C(20)-C(21), are 95.9° and 93.4°, respectively, confirming that they are perpendicular to each other. Furthermore, there are intramolecular interactions observed between hydrogen H(13) of the phenyl ring and hydrogen H(2d) of amine N(2) with nitrogen N(1).

### Solvatochromism studies

**Fig. 10:**
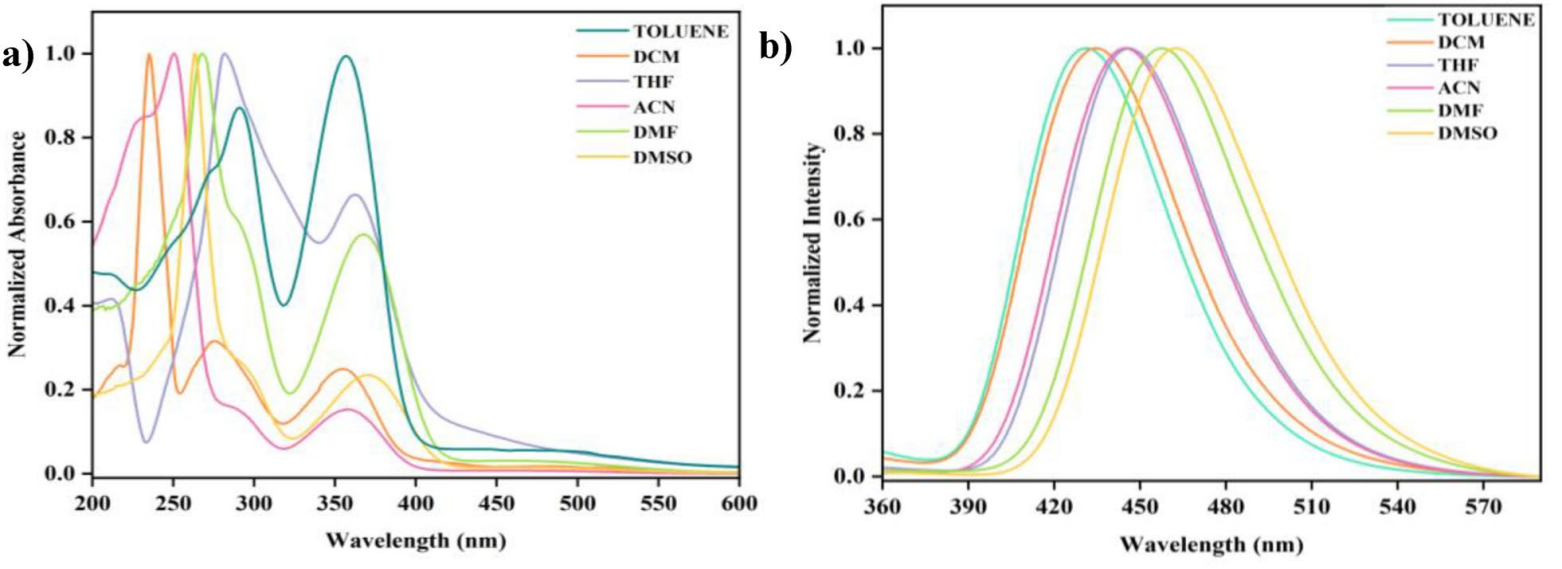
**a** Normalized absorption of **3e** in various solvents, **b** emission spectra of **3e** in various solvents

The synthesized fluorophore **3e** exhibits a bathochromic shift due to intra-ligand-charge transfer associated with the π-conjugation system present in the compound; it was further confirmed through solvatochromism studies (Fig. 10). The effect of various non-polar to polar solvents at 1 × 10^−5^ M concentration on compound **3e** was recorded using UV–Vis and Fluorescence spectroscopy (Fig. 10a and 10b). Toluene, DCM, THF, ACN, DMF, and DMSO were the solvents used to find the shift that is happening on **3e** in the increasing order of polarity. Table 4 shows the absorption λ_abs_ nm and emission λ_emi_ maxima of **3e** on various solvents along with their molar extinction coefficient and Stoke’s shift. The absorption spectra of **3e** give a lower absorption wavelength in the range of 263 to 291 nm due to π–π* electronic transition [intramolecular charge transfer, (ICT)] whereas it also gives an absorption peak at a higher wavelength from 356 to 370 nm because of n-π* transition. The corresponding emission band was observed in the region between 430 and 457 nm. As the polarity of the solvent is gradually increased from toluene to DMSO, a bathochromic shift (red shift) of 14 nm for the absorption spectra and 32 nm for the emission spectra can be observed in compound 3e. Also, **3e** exhibited absorption and emission bands with large stoke shifts from 11,108 cm^−1^ to 16,377 cm^−1^ due to ICT associated with the π conjugation system present in the compound [31]. Also, the solvatochromism studies on the remaining compounds have been done which showed less shift in the wavelength (Table S7). The spectra are shown in the supplementary data (Fig. S56 and S57).

**Table 4:** Photophysical properties of the compound **3e**

Entry	Solvent	Absorption, λ_abs_ (nm)	Emission, λ_emi_ (nm)	Molar extinction coefficient × 10^4^ (Ɛ)	Stoke’s Δ × 10^4^ v (cm^−1^)
1	Toluene	291,354	430	0.7409	1.1108
2	DCM	268,367	434	1.4883	1.4279
3	THF	281,363	445	3.9495	1.3115
4	ACN	250,358	445	1.5107	1.7528
5	DMF	267,367	457	1.4883	1.5571
6	DMSO	263,370	462	1.3464	1.6377

### UV–Vis and fluorescence studies

#### Selectivity studies and interference studies

**Fig. 11:**
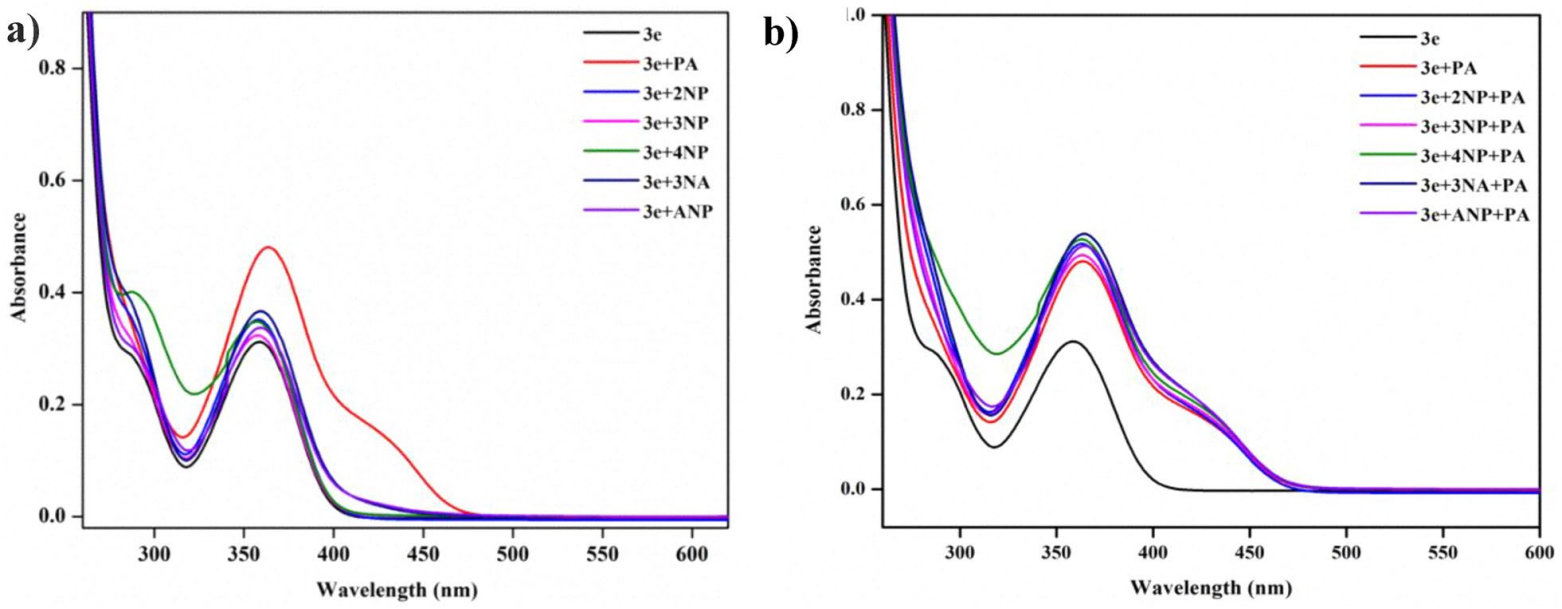
**a** UV–Vis absorption selectivity studies of **3e** (20 µL) with picric acid and other nitro compounds, **b** absorption spectrum of **3e** (20 µL) with picric acid and other interfering species

**Fig. 12:**
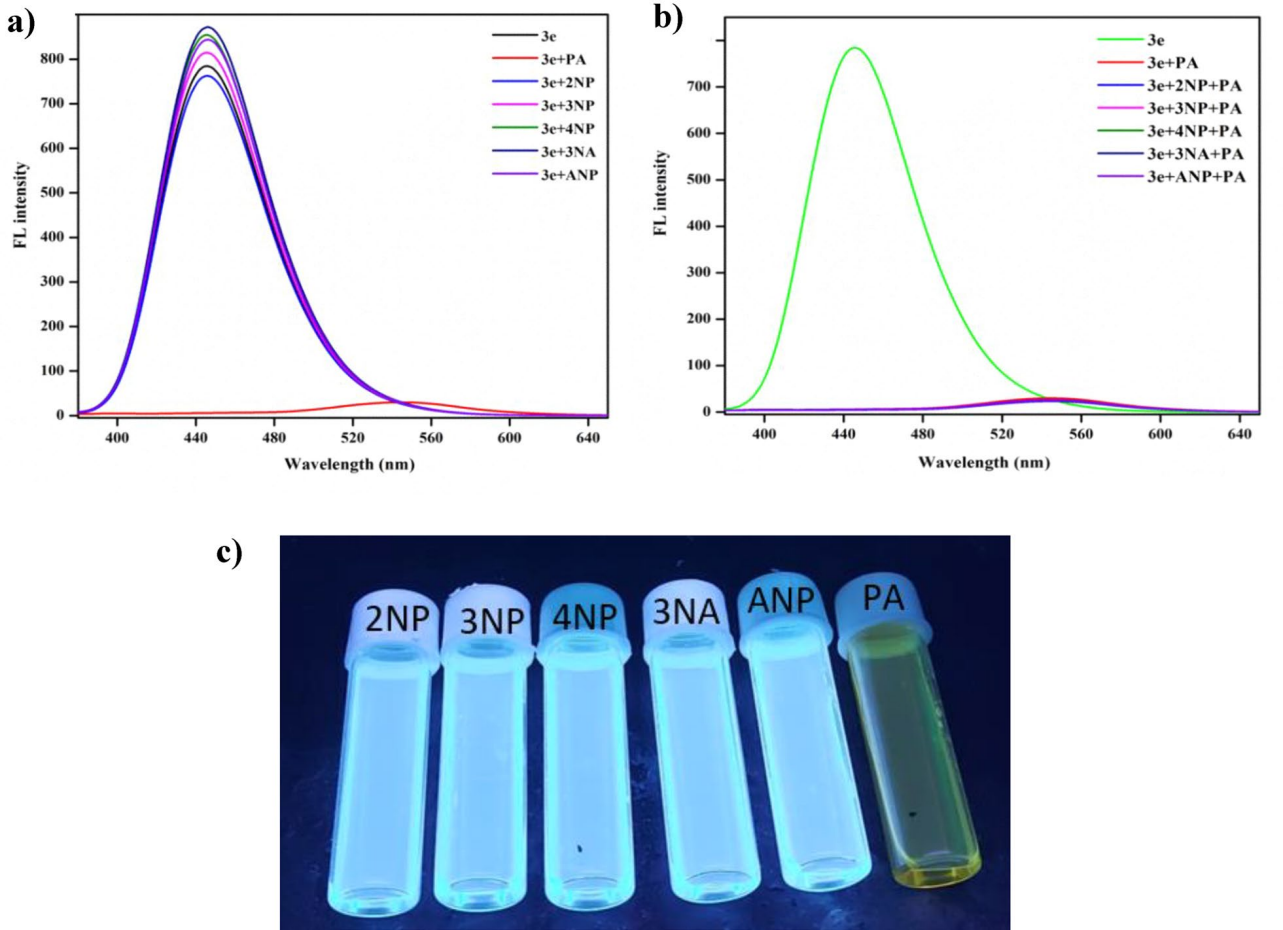
**a** Emission selectivity studies of **3e** (20 µL) with PA and other nitro compounds. **b** Emission spectrum of **3e** (20 µL) with PA and other interfering species. **c** Selectivity studies under UV light (365 nm)

The selectivity of PA was studied over the other nitro aromatics such as 2-nitrophenol (2NP), 3-nitrophenol (3NP), 4-nitrophenol (4NP), 3-nitro aniline (3NA), 2-amino-4-nitrophenol (ANP) by examining the absorbance and emission of **3e** in acetonitrile. The stock solution preparation is explained in detail in the supplementary information (S.3). Also, the comparison table of the present work along with the earlier reports is given in supplementary information Table S8. 20 µL of 1 × 10^–3^ M solution of the selected nitrophenols were added to the probe, observing an absorbance change for PA alone from 0.3 to 0.5, whereas the other analytes' absorbance remained the same. The sensing efficiency can be determined based on how highly the probe can select the target analyte compared to other interfering ions. **3e** subjected to absorbance and fluorescence studies with different nitro analytes in a quartz cuvette (1 × 1 cm) containing 2 mL acetonitrile of 1 × 10^–5^ M of the prepared probe. From the absorption spectra and emission spectra of various analytes towards the probe (Figs. 11a and 12a), it was clear that **3e** can sense PA accurately with good selectivity. The fluorometric response of various nitro compounds also examined the selectivity of sensor 3e. For probe 3e, excitation at 354 nm resulted in an emission band of the fluorescence spectra observed at 445 nm. Notably, when PA was added to the probe, the fluorescence intensity decreased significantly due to quenching. In contrast, the fluorometric response of other nitro compounds with the probe showed no significant changes. This quenching behaviour supports the conclusion that the probe can serve as a receptor for the selective detection of PA and function as a fluorometric chemosensor. Further investigations were conducted to assess the selectivity of probe 3e in the presence of other interfering nitro compounds. The results shown in Figs. 11b and 12b indicate that there were no significant changes in fluorescence intensity, demonstrating that the other nitro compounds did not impact the detection of PA.

#### Sensitivity and Job′s plot studies

**Fig. 13:**
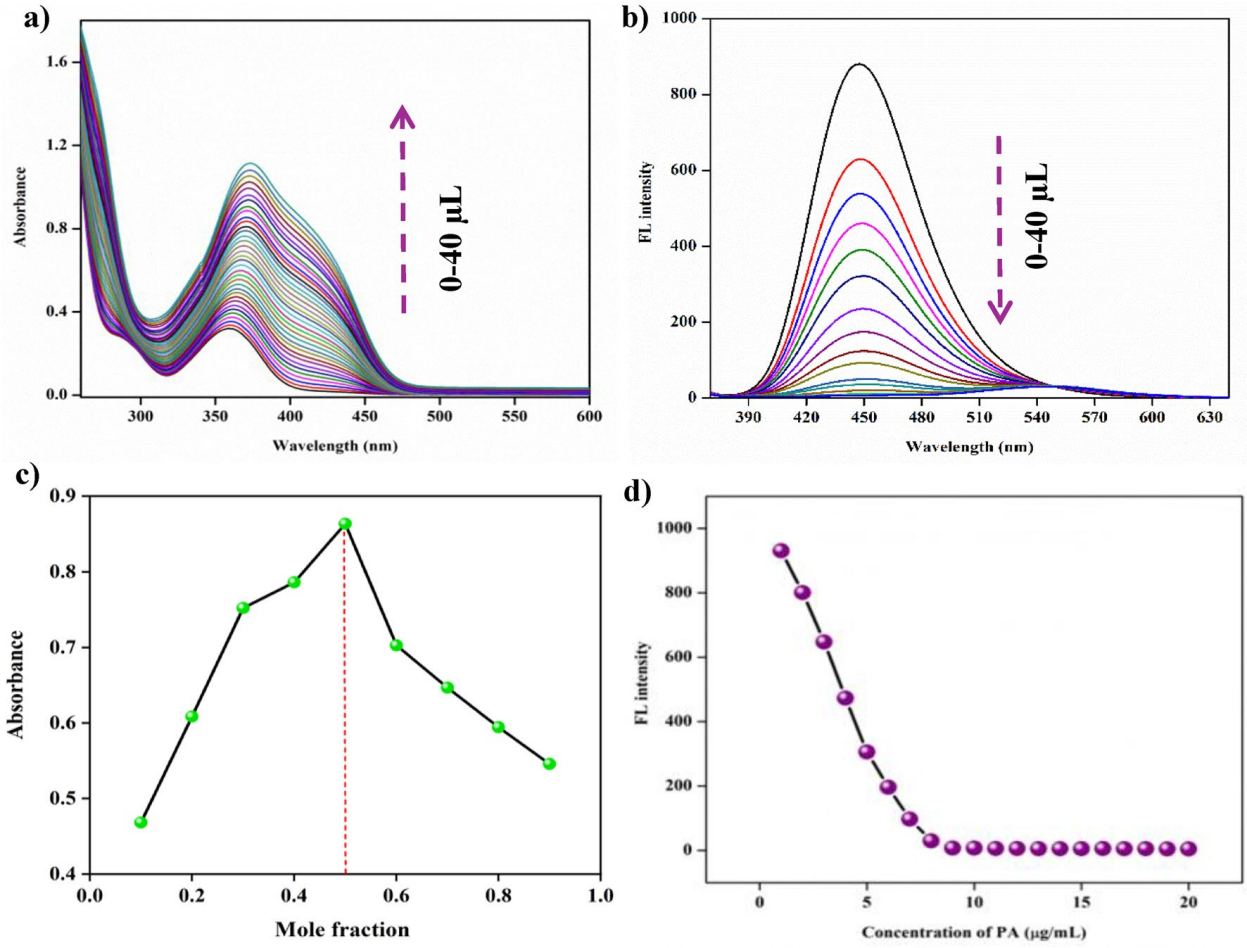
**a** UV–visible absorption spectrum for titration studies of **3e** with gradual addition of picric acid. **b** Emission spectrum for titration studies of **3e** with the gradual addition of picric acid. **c** Job’s plot of **3e** with picric acid. **d** The relationship between the fluorescence intensity variation and the picric acid concentration

20 µL aliquots of a 1 × 10^–3^ M solution of probe 3e were dissolved in 2 mL of acetonitrile solvent. The absorption titration was recorded with the incremental addition of 2 µL of PA. The significant changes observed in the electronic spectra of 3e can be attributed to hydrogen bonding interactions between the hydroxyl group (-OH) of picric acid and the amino group (-NH_2_) of probe 3e. (Fig. 13).

The detailed study of the interaction between them was done by doing the fluorometric titration on addition of 1 µL of picric acid to 20 µL of the probe dissolved in 1 × 10^–3^ M concentration acetonitrile solvent. A decrease in the emission intensity was observed with which quenching of fluorescence can be proven. Electron donating groups like -NH_2_, and-OH can increase the fluorescence of a compound due to the presence of a lone pair which makes the compound more electron-dense and involved in conjugation, thus absorbing more quantity of light and causing intense fluorescence when compared to electron-withdrawing groups. A drastic change in the emission spectra of 3e has been observed due to the interaction between the hydrogen bond of the hydroxyl group (-OH) of picric acid and the amino group (-NH_2_) of the probe **3e**. This is confirmed by studying Job's plot that shows the plotting between UV absorption versus mole fraction and the maximum curve implies the stochiometric binding ratio between the analyte and the probe [32]. Maximum absorption was obtained at 0.5 mol fraction by connecting the points obtained from the Jobs plot and was found that the binding stoichiometry is at 1:1.

#### The limit of detection and Stern–Volmer quenching constant measurement

**Fig. 14:**
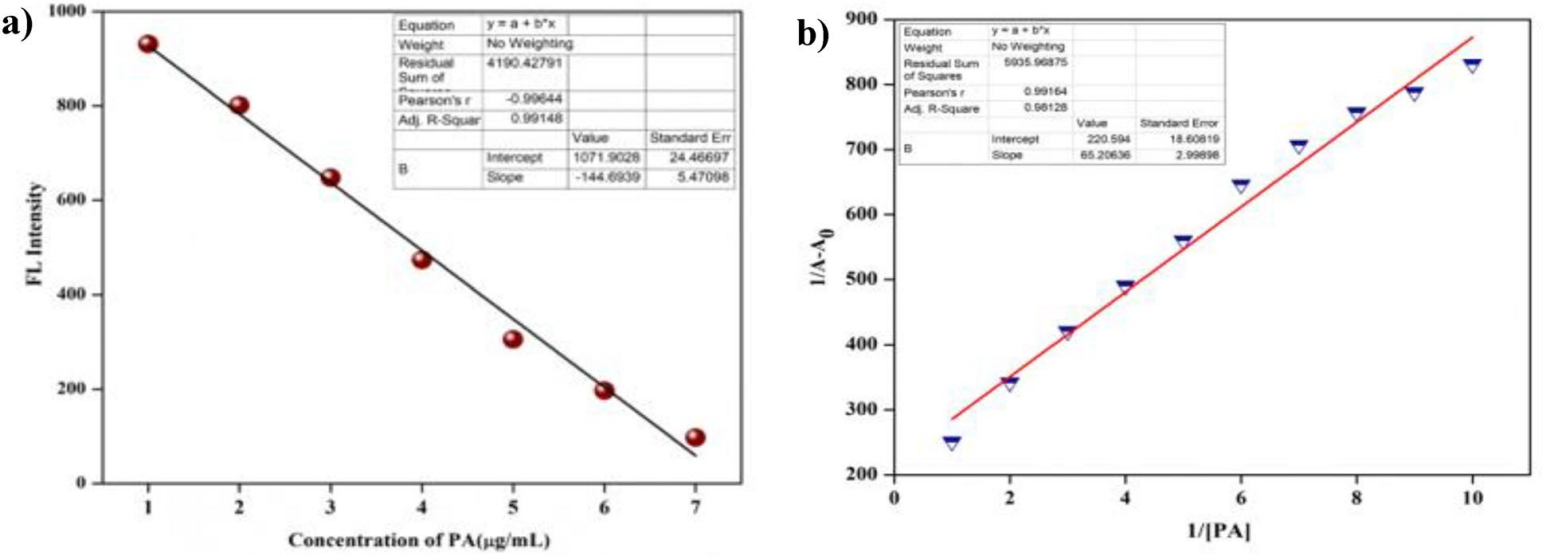
**a** Graph showing LOD of PA. **b** At lower concentration of PA, emission quenching linearity relationship

The limit of detection was calculated using the formula, LOD = 3 $\sigma$/slope, where the standard deviation of the blank solution is represented as $\sigma$. The detection limit [33] was found to be 1.766 × 10^−9^ M and the linearity coefficient R^2^ = 0.99148 illustrated in (Fig. 14a) The quenching efficiency of **3e** was quantified using the Stern–Volmer equation [34] I_0_/I = 1 + K_sv_ [Q] where I_0_ and I represent emission intensities before and after the addition of PA, and [Q] represents the concentration of **3e**. At low concentrations, the linear plot was obtained and the Stern–Volmer constant (K_sv_) was determined to be 6.52 × 10^–6^ M (Fig. 14b).

#### Reversibility studies

**Fig. 15:**
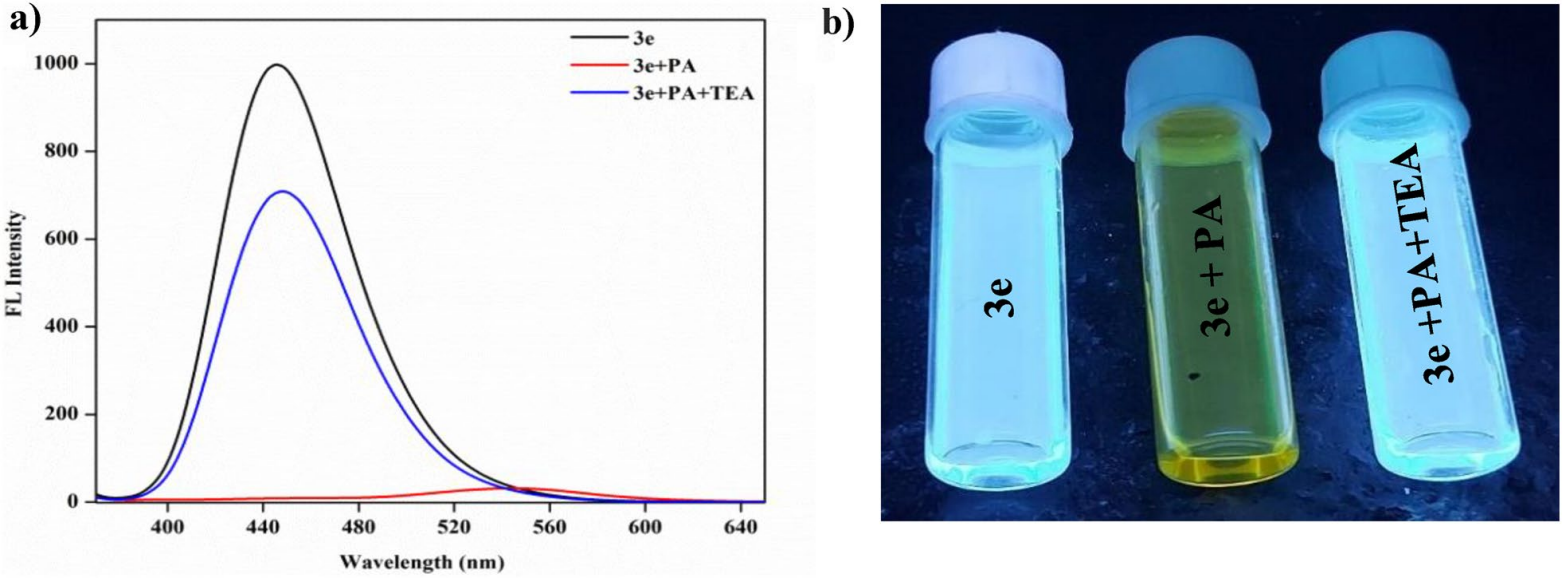
**a** Fluorescent spectra on reversibility studies with probe **3e**, **3e** + PA and **3e** + PA + TEA. **b** Reversibility studies under UV light (365 nm)

Reversibility studies have been conducted to look ino the effects of reversible coordination. Studies were carried out with the help of triethyl amine (TEA). The addition of picric acid to the probe causes a notable decrease in fluorescence intensity of **3e** which is regained upon adding 0.2 µL of TEA (Fig. 15). Thus the probe can be used as an efficient turn-off sensor for the detection of PA.

#### Plausible binding mechanism

**Fig. 16:**
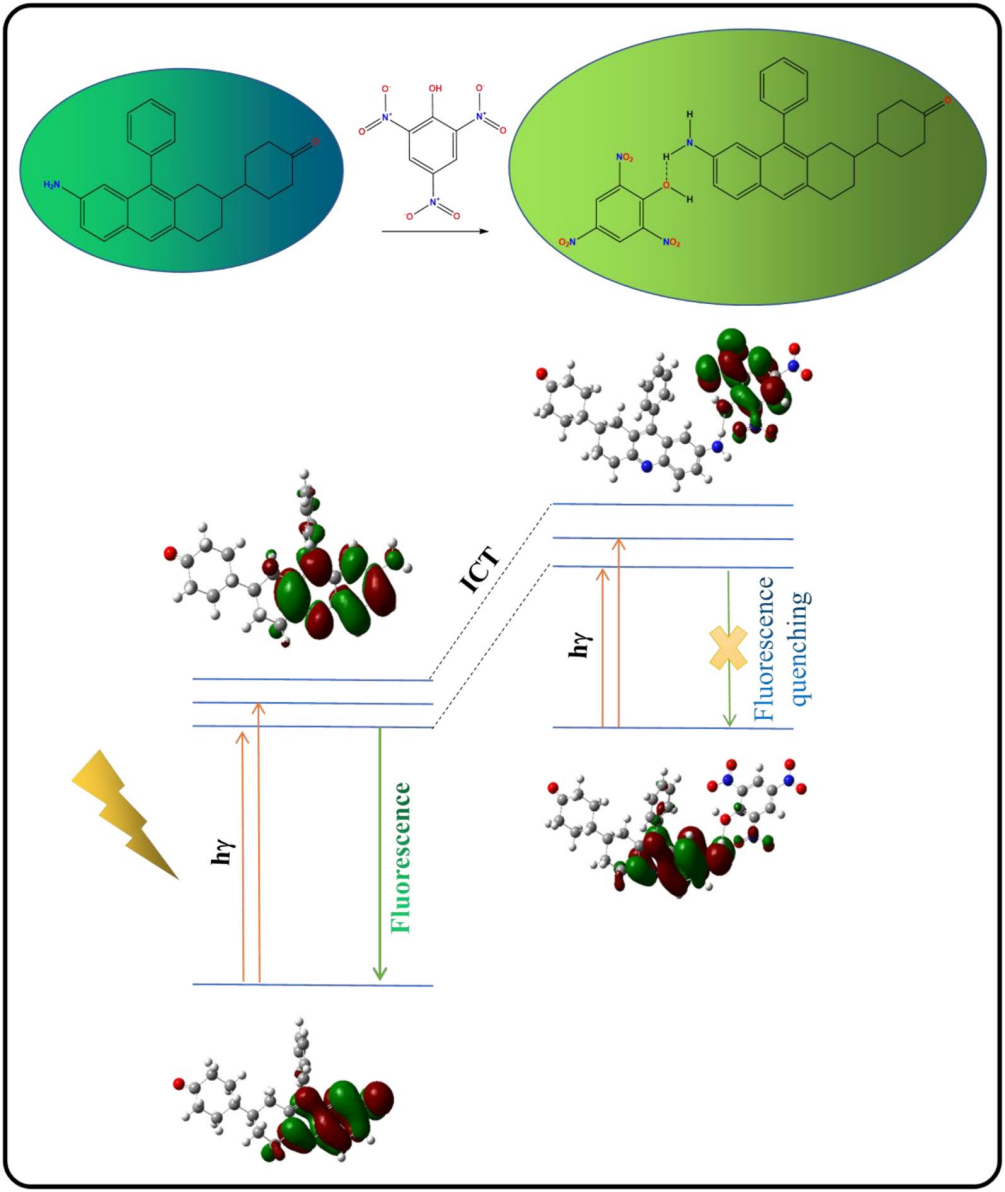
Plausible binding mechanism of probe **3e** interaction with PA

Fluorescence sensing can be classified as “turn on” as well as “turn off” types that depend on various quenching mechanisms namely Photoinduced Electron Transfer (PET), Intramolecular Charge Transfer (ICT), Forster Resonance Energy (FRET), Aggregation-Induced Emission (AIE). These mechanisms involve the transfer of electron density from the excited states of the fluorophore to the analyte either leading to an increase or decrease in fluorescence intensity [35]. To investigate the binding mechanism of probe **3e** with PA, studies like NMR titration, FT-IR, and DFT studies have been conducted and explained in detail. Photo-induced electron transfer (PET) occurs when the analyte PA is added to probe **3e** which is explained in the mechanism given in Fig. 16.

#### ^1^H NMR Titration

**Fig. 17:**
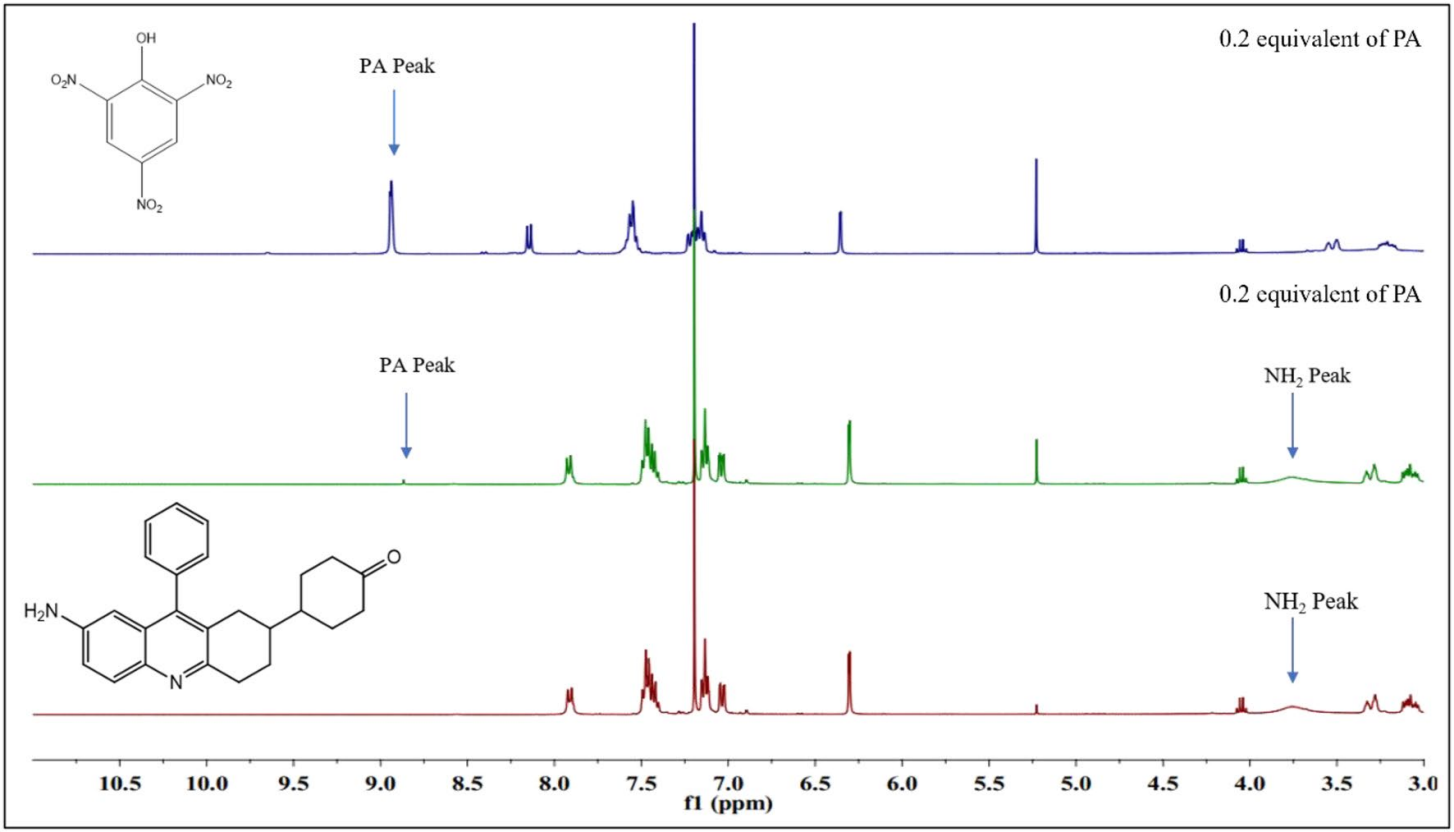
^1^H NMR titration of probe **3e** with PA in CDCl_3_

^1^H NMR titration of probe **3e** was carried in CDCl_3_ solvent at ambient temperature by adding on 0.2 equivalent of the analyte PA to study the binding mechanism between the analyte and PA. The probe alone showed a singlet at δ3.4 ppm due to the presence of the NH_2_ group. The addition of picric acid results in a gradual decrease of the singlet peak at δ 3.4 ppm and completely disappears after one addition simultaneously a new singlet peak emerges at δ 8.9 ppm gradually due to the aromatic protons present in the PA whereas the -OH peak cannot be observed due to the intermolecular hydrogen bonding between the probe and the analyte. A gradual shift of the aromatic peaks (δ 7.92, 7.50–7.40, 7.19, 7.08, 6.36 ppm) of the probe 3e towards downfield (δ 8.23, 7.63–7.57, 7.28, 7.22, 6.42) can be observed, which is due to the release of electrons from the probe towards the analyte (Fig. 17). Thus, from the titration studies, we can conclude the interaction of the probe and analyte via strong intermolecular H-bonding interaction between the –OH moiety of PA and the –NH_2_ moiety of probe **3e**.

#### FT-IR studies

**Fig. 18:**
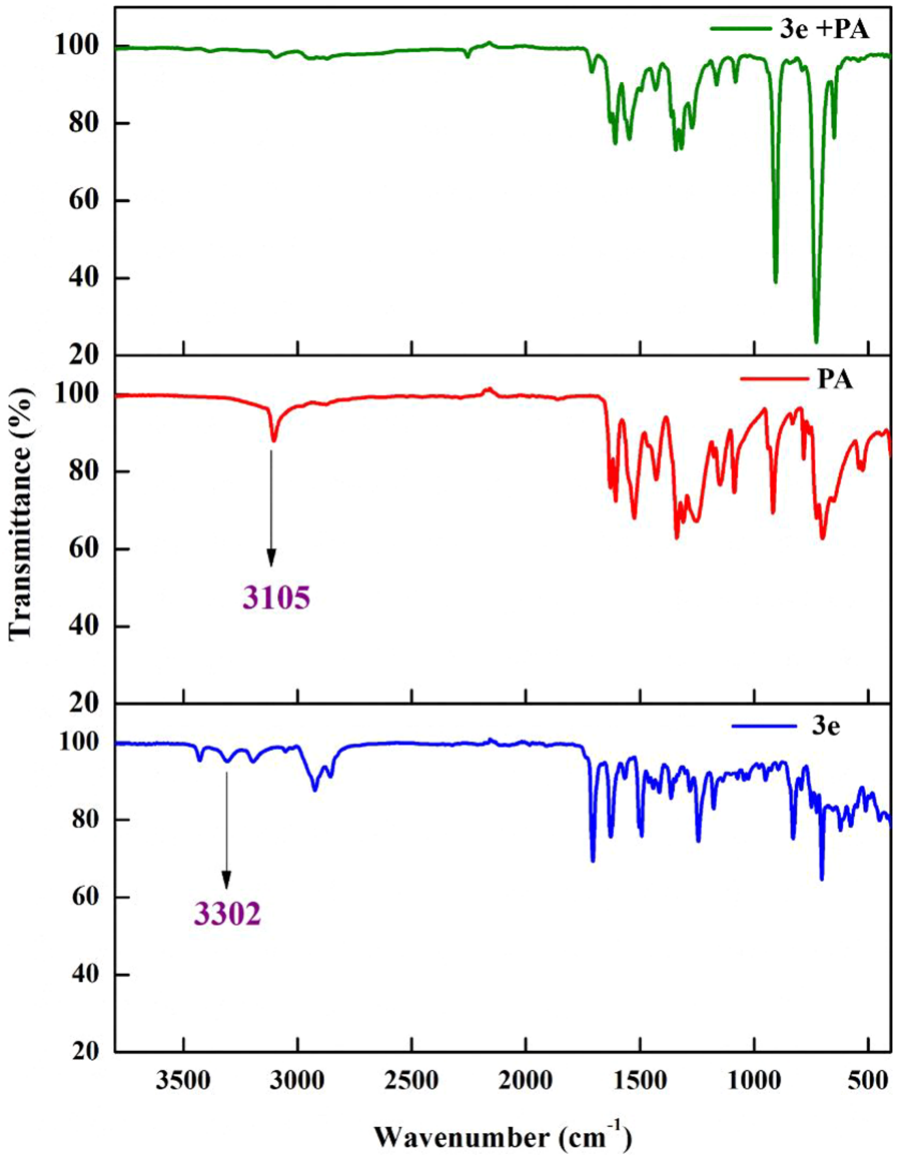
FT-IR spectra of probe **3e**, PA, and probe **3e** after the addition of picric acid

The binding mechanism of the probe and analyte was also confirmed using FTIR studies. The FTIR data of probe **3e**, analyte PA, and probe after the addition of the analyte is shown in Fig. 18. The NH_2_ peak at 3302 cm^−1^ of the probe as well as the OH peak at 3105 cm^−1^ of the analyte diminishes on adding the analyte to the probe. Hence proves the binding of -NH_2_ and -OH moiety during the complex formation.

#### Binding mechanism of probe-analyte via TD-DFT

**Fig. 19:**
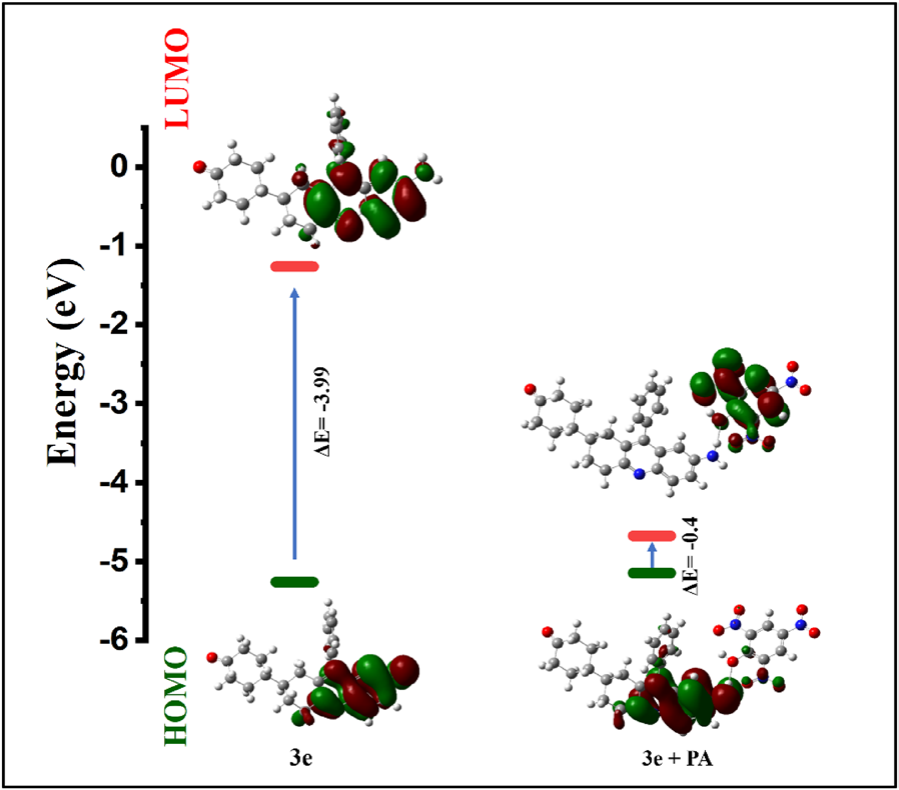
FMO diagram of **3e** and **3e** + PA

Time-dependent density functional theory (TD-DFT) studies on **3e** and **3e** + PA further confirmed the binding mechanism and energy change occurring in the probe alone and probe with the analyte. The frontier molecular orbital (FMO) diagram of the probe before and after the addition of the analyte is represented in Fig. 19. Electron density is distributed across the quinoline moiety because of the presence of -NH_2_ (an electron-donating group) in the HOMO and LUMO energy states of the probe. Adding upon the electron-deficient analyte PA shows the movement of electron density towards PA in the LUMO excitation state [36]. Here, we can observe a decrease in the energy gap between HOMO and LUMO of **3e** + PA when compared with probe **3e** alone. **3e + PA** is stabilized by an energy of 0.4 eV, indicating that PA has a strong bond with probe 3e. The selected transitions which exhibit the percentage of major contributions of Inter Charge Transfer (ICT) obtained were tabulated in the supporting information (Table S9) along with the HOMO and LUMO orbital images. (Table S10 and S11).

## Conclusion

In conclusion, three different methods have synthesized a series of quinoline-based acridines. Among these, the one-pot synthesis using less toxic and more affordable DES has given desired products in a simpler and greener way. Green metric calculations further demonstrate also indicate that the newly synthesized compounds using DES enhance sustainability and reliability in the synthesis process proving to be a simple and eco-friendly option. A theoretical and experimental study was conducted on the synthesized compounds, including density functional studies for spectral calculations through the Gaussian program based on the B3LYP method and the 6-311G basis set and binding affinity assessments with Bovine Serum Albumin (BSA) using AutoDock Vina 4.2. Furthermore, the novel quinoline-based acridine moiety (3e) was designed and confirmed through various characterization techniques, including SC-XRD. It was analysed for its selective detection of 2,4,6-trinitrophenol (PA), a nitro explosive. Photophysical investigations determined a 1:1 binding stoichiometric ratio between the probe and analyte, with a LOD of 1.766 × 10^–9^ M. Compound 3e exhibits absorption at 354 nm and emission at 445 nm, demonstrating a “turn-off” fluorescence chemosensor when PA is added. The underlying binding mechanism was further elucidated through ^1^H NMR titration, FTIR, and TD-DFT techniques.

## Supplementary Information

Supplementary material 1.

## Data Availability

Submitted as Supplementary Data along with the manuscript.
