# Supplementary material for: Synthesis and in silico studies of quinoline appended acridine via conventional and green methods: photophysical analysis of novel fluorophore for picric acid detection using a ‘turn-off' fluorescence approach
Source: BMC Chem. 2025 Apr 9;19(1):93. doi: 10.1186/s13065-025-01452-y (PMC11983936; doi:10.1186/s13065-025-01452-y)
Supplement: Supplementary file 1 — Supplementary material 1. [file 13065_2025_1452_MOESM1_ESM.docx]

**Synthesis and *In Silico* studies of quinoline appended acridine via conventional and green methods: Photophysical analysis of novel fluorophore for picric acid detection using a ‘Turn-Off' Fluorescence Approach**

**Rebecca Susan Philip^1^, V. Vijayakumar^1^***

^1^Department of Chemistry, School of Advanced Sciences, Vellore Institute of Technology, Vellore-632014, India

^1^*Department of Chemistry, School of Advanced Sciences, Vellore Institute of Technology, Vellore-632014, India

| **S.No** | **Table of Contents** | **Page no.** |
| --- | --- | --- |
| **1.** | **Spectral characterization of 3a to 4d** | **S2-S24** |
| **2.** | **Various Deep Eutectic Solvents with their molecular ratio** | **S25** |
| **3.** | **Physical properties of 3a to 4d calculated using DFT** | **S26** |
| **4.** | **Selected transitions obtained from TD-DFT calculation of 3a to 4d** | **S27** |
| **5.** | **Docking energy results of 3a to 4d** | **S28** |
| **6.** | **Molecular docking 3D and 2D images of 3a to 4d** | **S29-S33** |
| **7.** | **Crystal data and structure refinement for 3e** | **S34** |
| **8.** | **Selected Bond Lengths, Bond Angles, and Torsional angles for 3e** | **S35** |
| **9.** | **Solvatochromism studies of 3a to 4d** | **S35-S37** |
| **10.** | **General procedure for the preparation of the stock solution of 3e** | **S37** |
| **11.** | **Comparison of LOD of present work with earlier reports** | **S38** |
| **12.** | **Selected transitions obtained from TD-DFT calculation of 3e and 3e+PA** | **S38** |
| **13.** | **FMO of 3e and 3e+PA with energy in eV** | **S39-S42** |
| **14.** | **References** | **S42-S43** |

**`**

**S.1. Spectral characterization of compounds 3a to 4d**


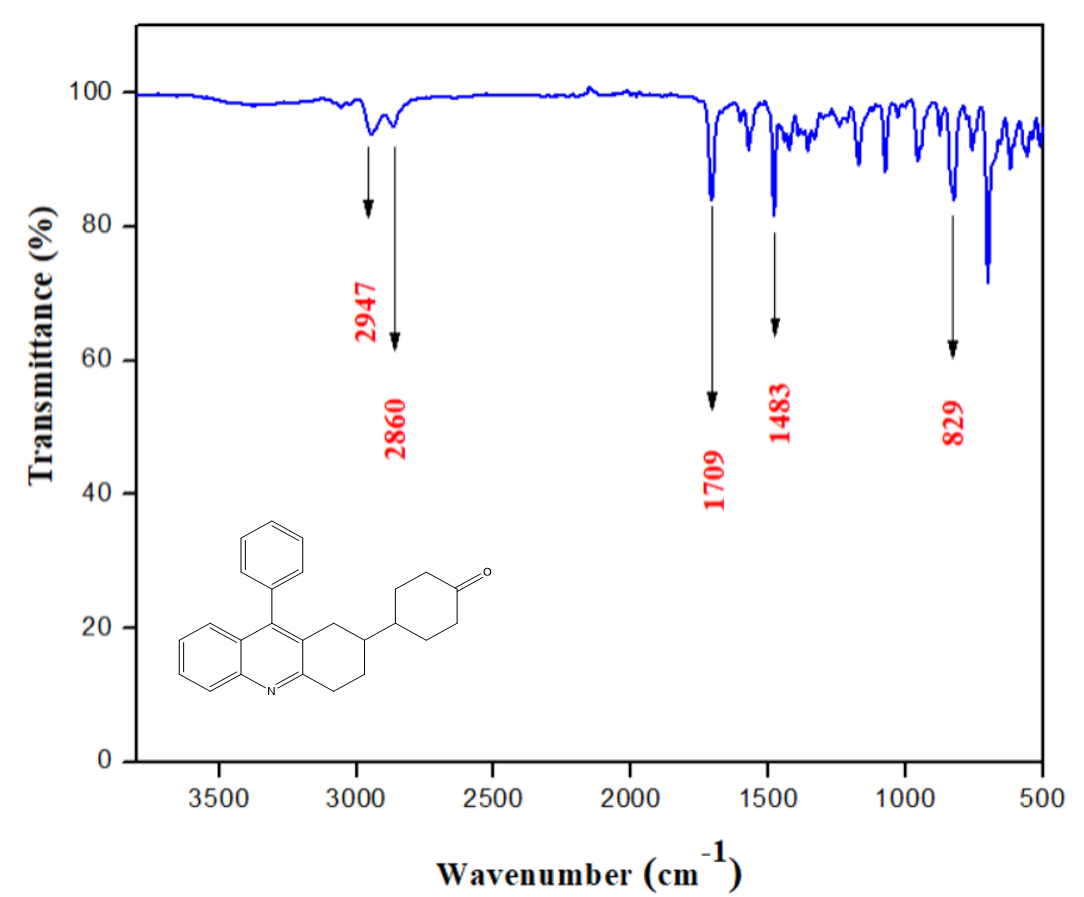


**Figure S1. FTIR spectrum of 4-(9-phenyl-1,2,3,4-tetrahydroacridin-2-yl)cyclohexan-1-one (3a)**


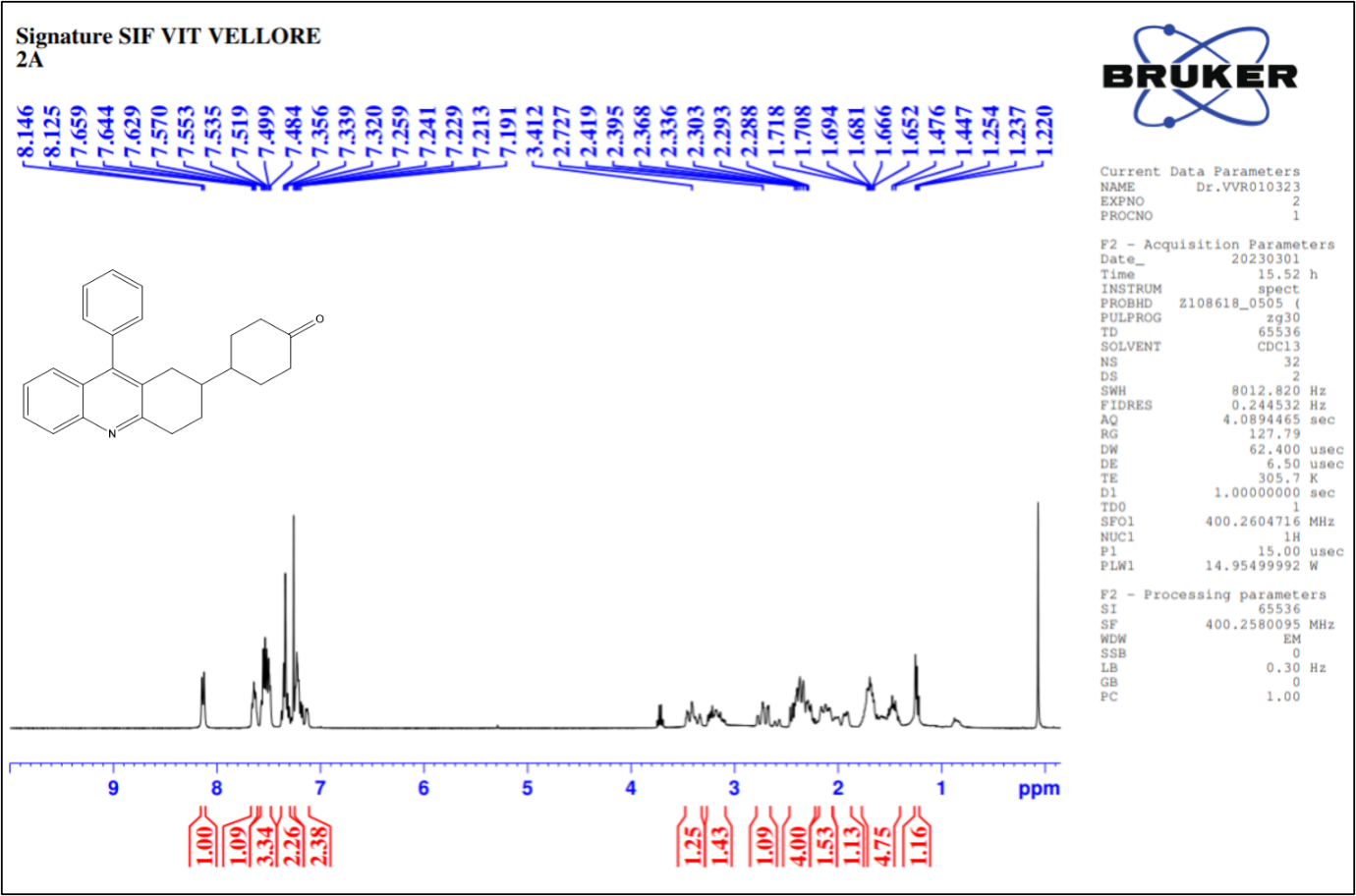


**Figure S2. ^1^H- NMR spectrum of 4-(9-phenyl-1,2,3,4-tetrahydroacridin-2-yl)cyclohexan-1-one (3a)**

**
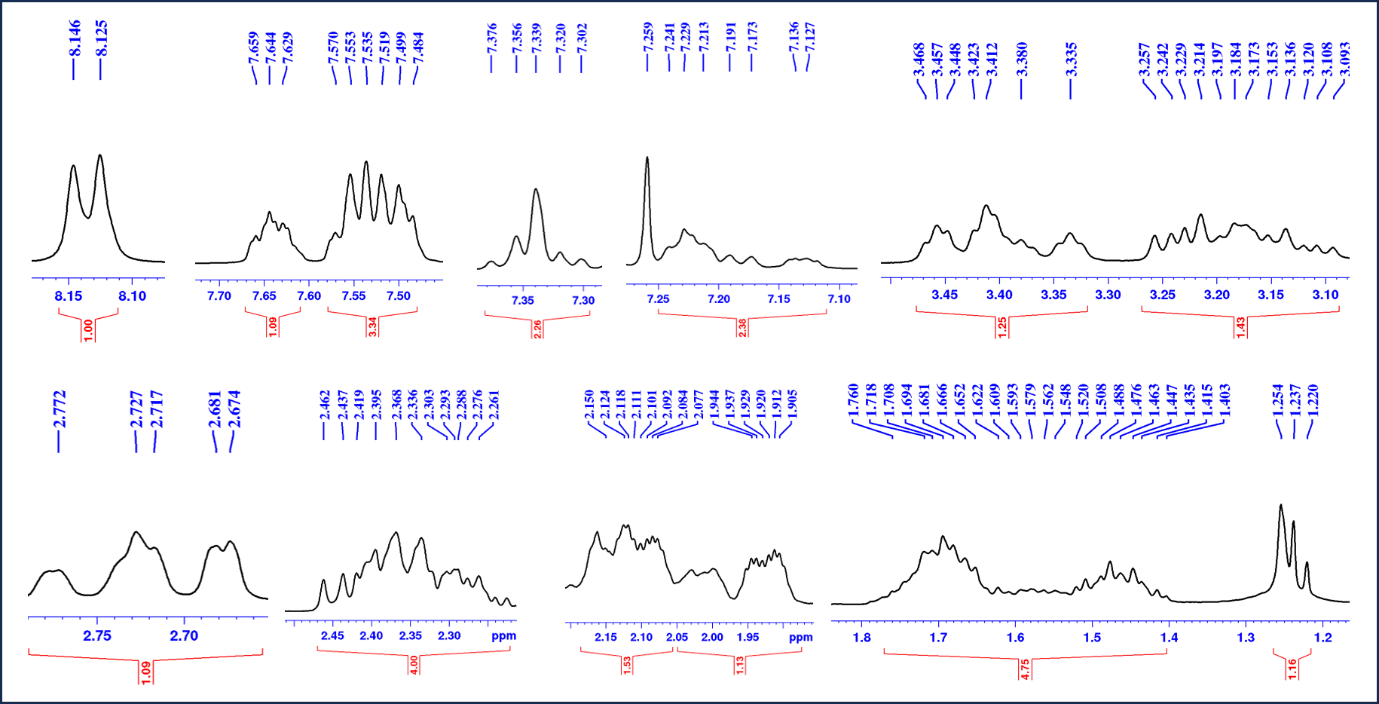
**

**Figure S3. Enlarged ^1^H- NMR spectrum of 4-(9-phenyl-1,2,3,4-tetrahydroacridin-2-yl)cyclohexan-1-one (3a)**


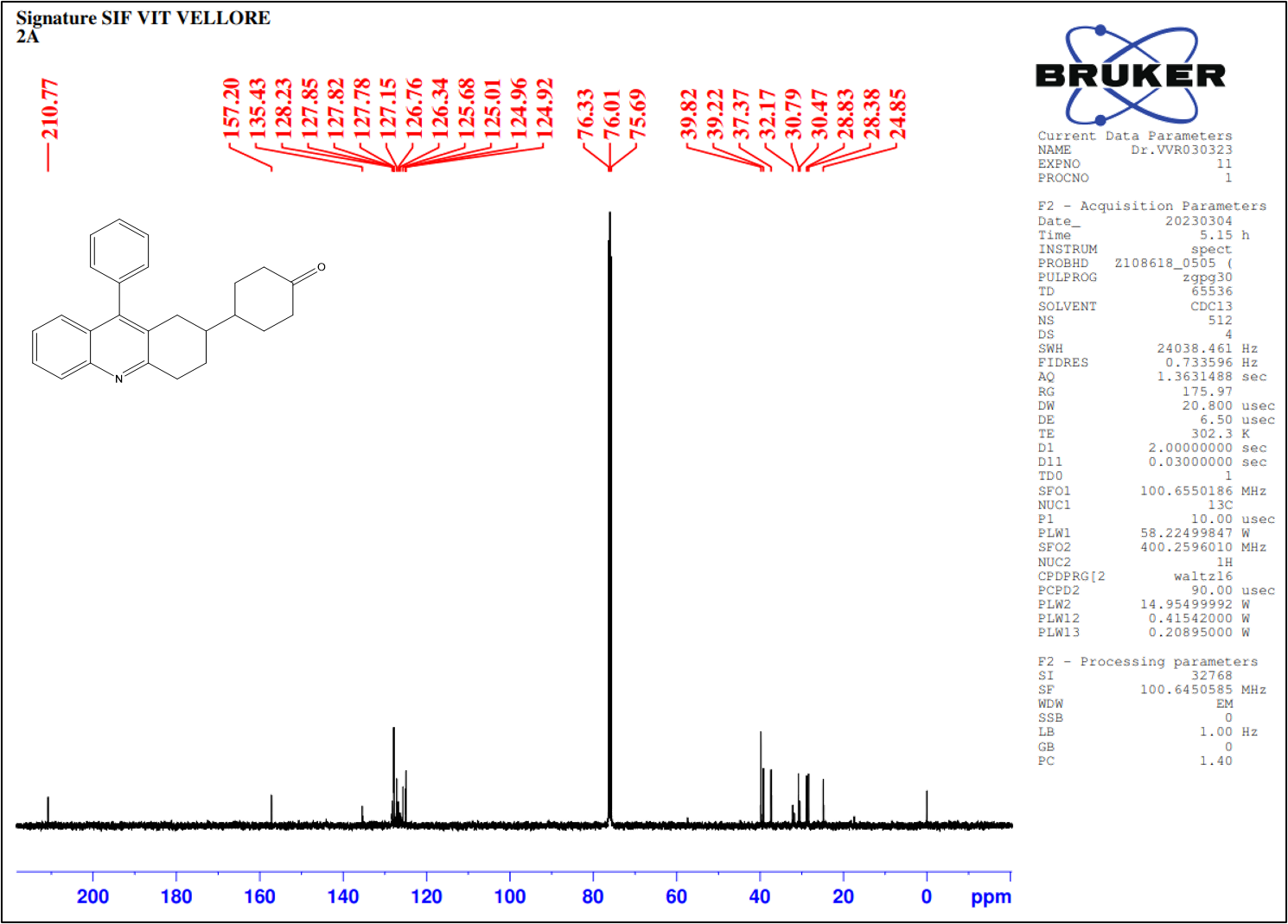


**Figure S4. ^13^C- NMR spectrum of4-(9-phenyl-1,2,3,4-tetrahydroacridin-2-yl)cyclohexan-1-one (3a)**


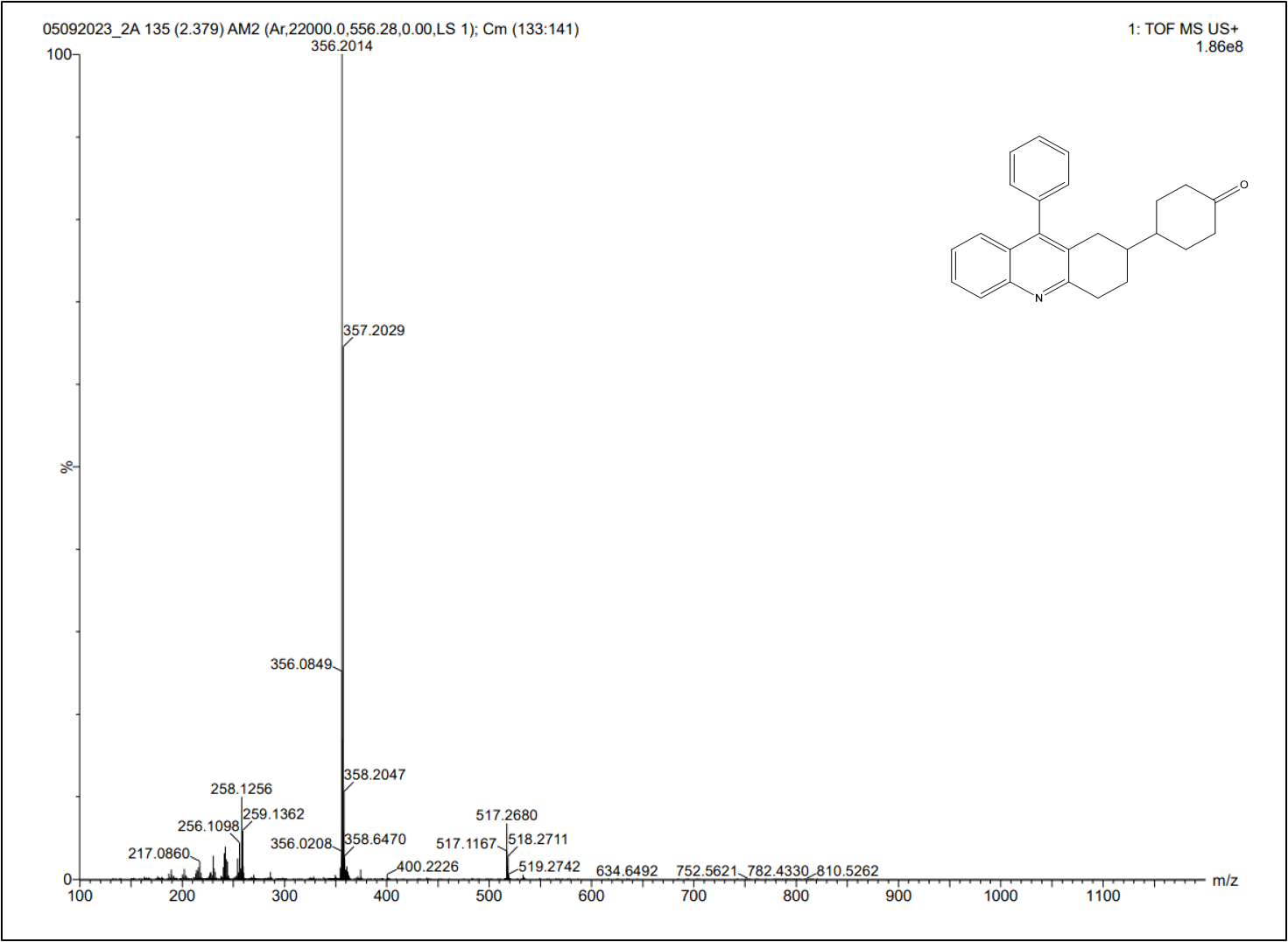


**Figure S5. HRMS spectrum of 4-(9-phenyl-1,2,3,4-tetrahydroacridin-2-yl)cyclohexan-1-one (3a)**


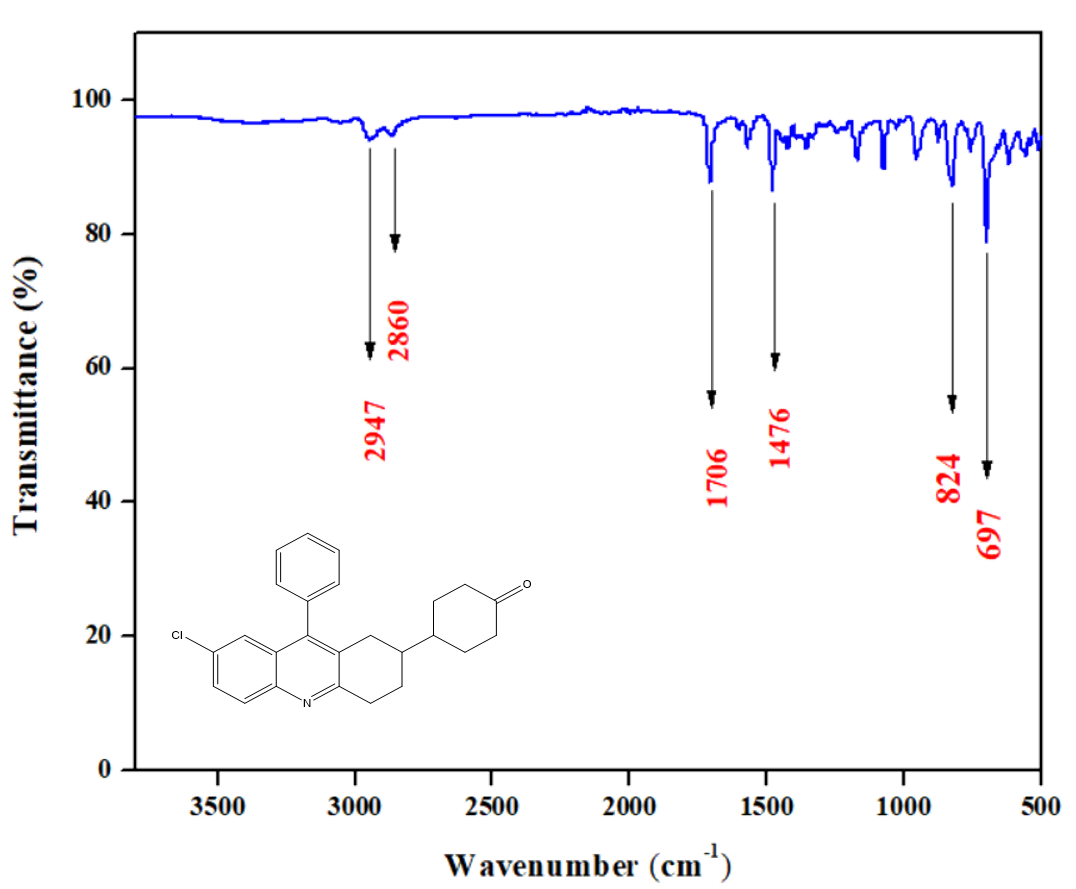


**Figure S6. FTIR spectrum of 4-(7-chloro-9-phenyl-1,2,3,4-tetrahydroacridin-2-yl)cyclohexan-1-one (3b)**


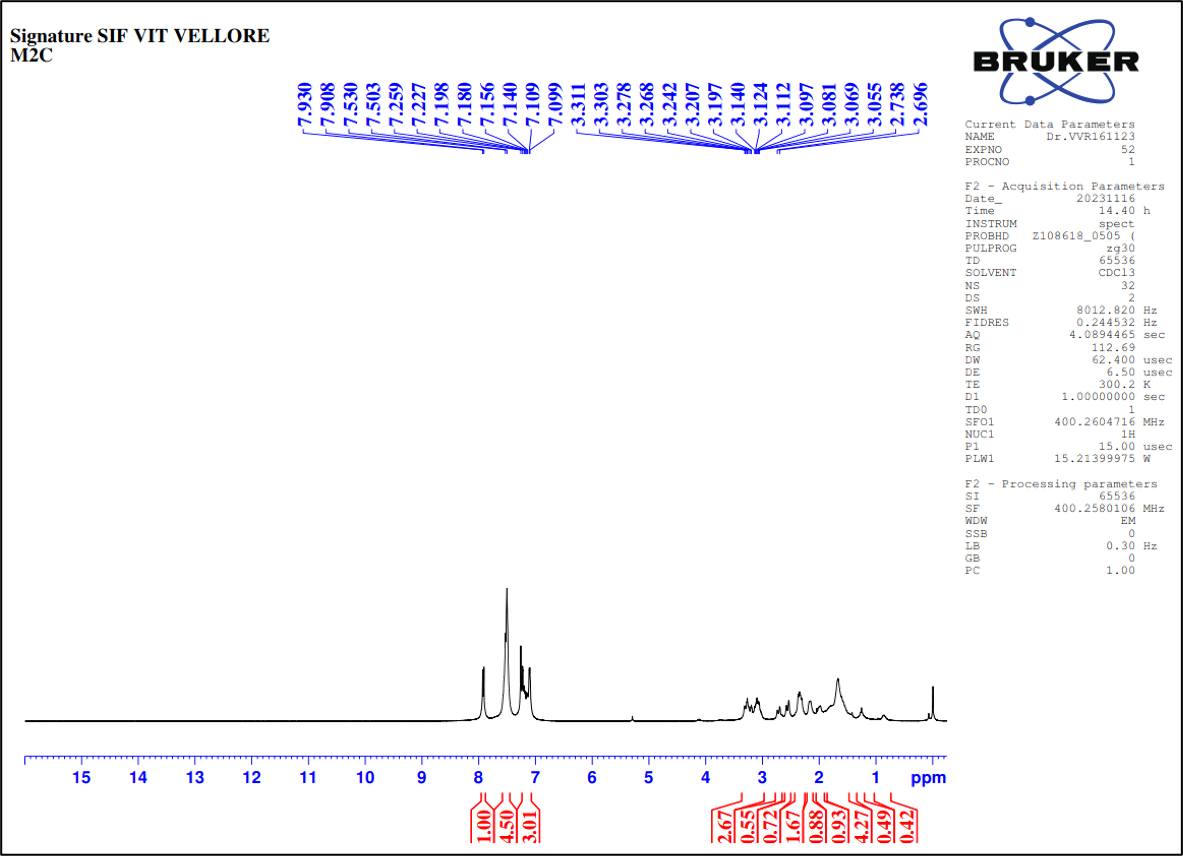


**Figure S7. ^1^H- NMR spectrum of 4-(7-chloro-9-phenyl-1,2,3,4-tetrahydroacridin-2-yl)cyclohexan-1-one (3b)**

**
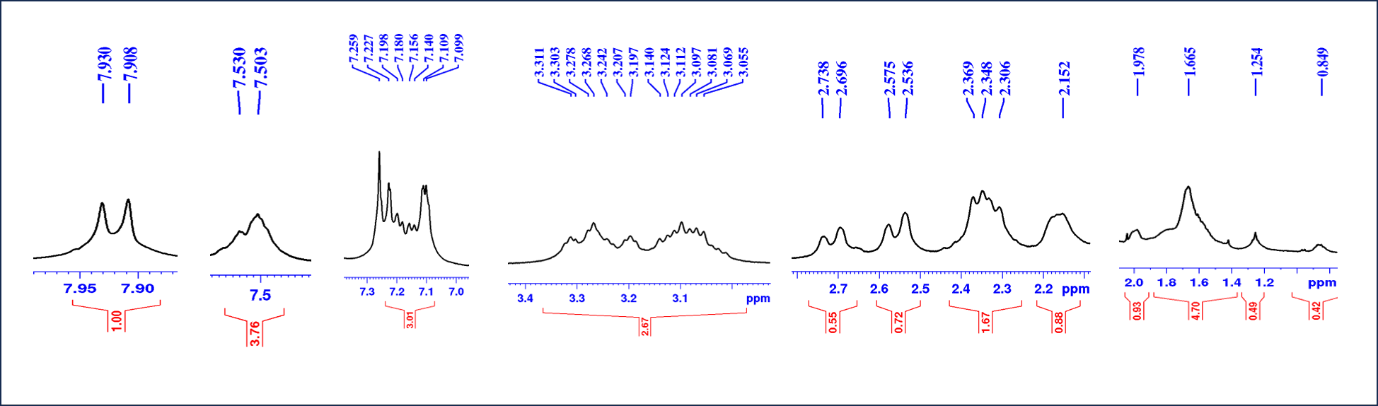
**

**Figure S8. Enlarged ^1^H- NMR spectrum of 4-(7-chloro-9-phenyl-1,2,3,4-tetrahydroacridin-2-yl) cyclohexan-1-one (3b)**


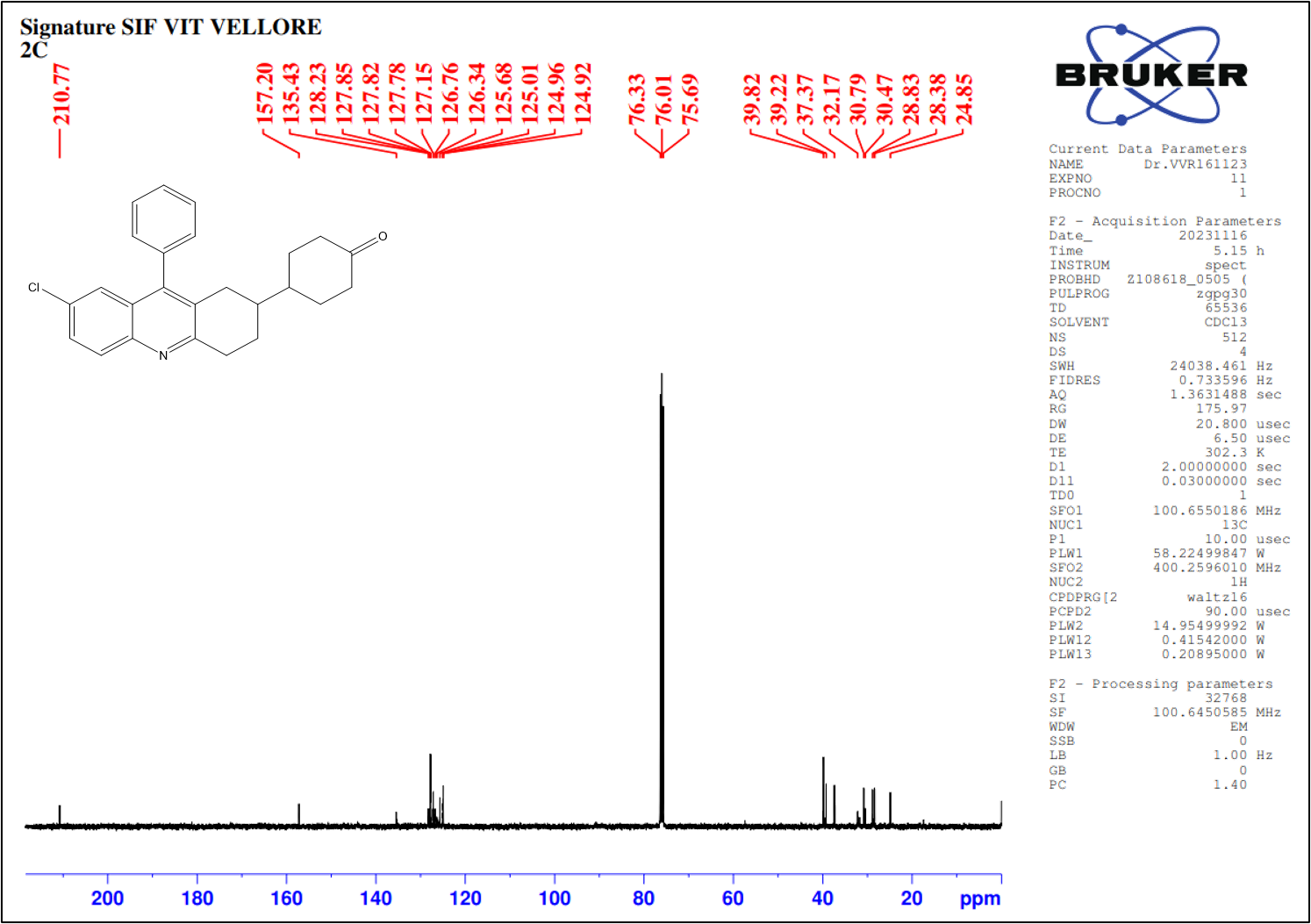


**Figure S9. ^13^C- NMR spectrum of 4-(7-chloro-9-phenyl-1,2,3,4-tetrahydroacridin-2-yl)cyclohexan-1-one** **(3b)**


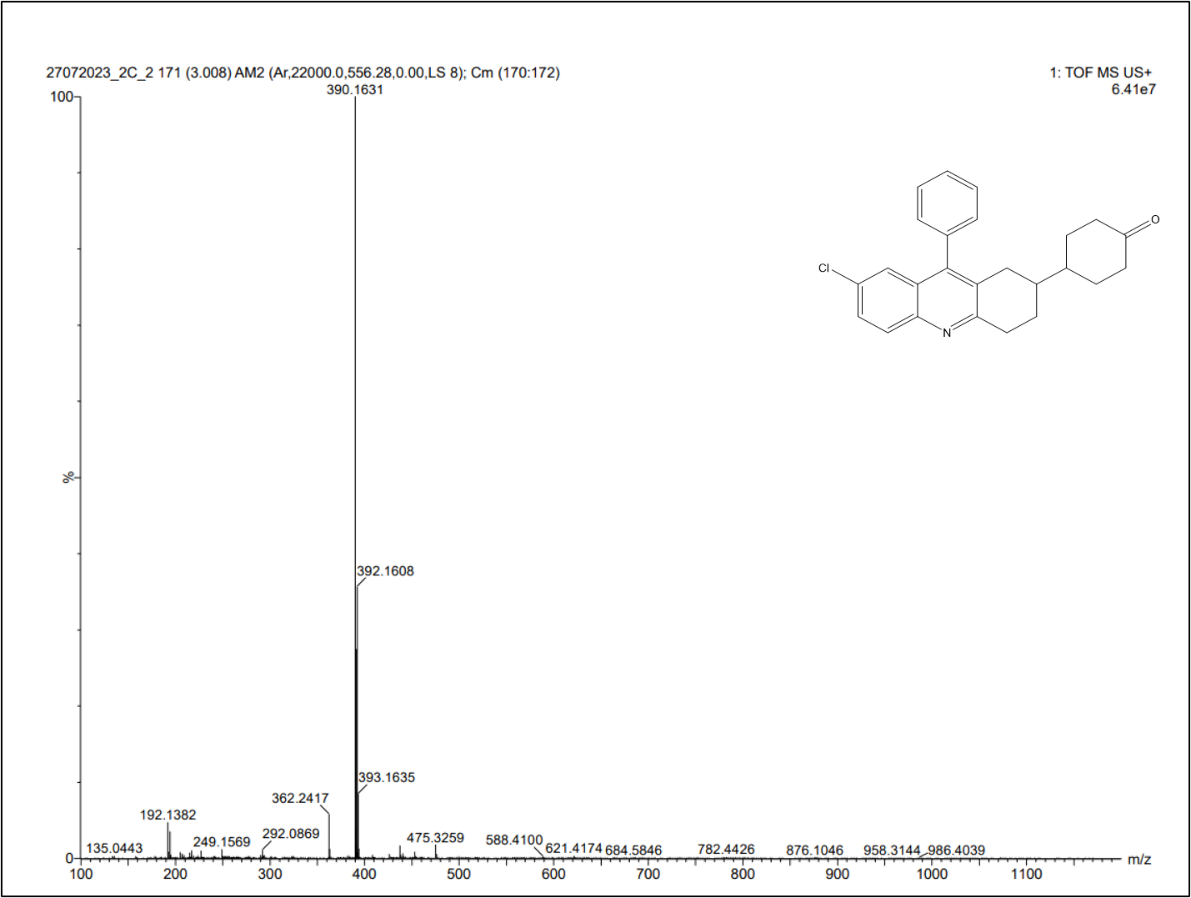


**Figure S10. HRMS spectrum of 4-(7-chloro-9-phenyl-1,2,3,4-tetrahydroacridin-2-yl)cyclohexan-1-one (3b)**


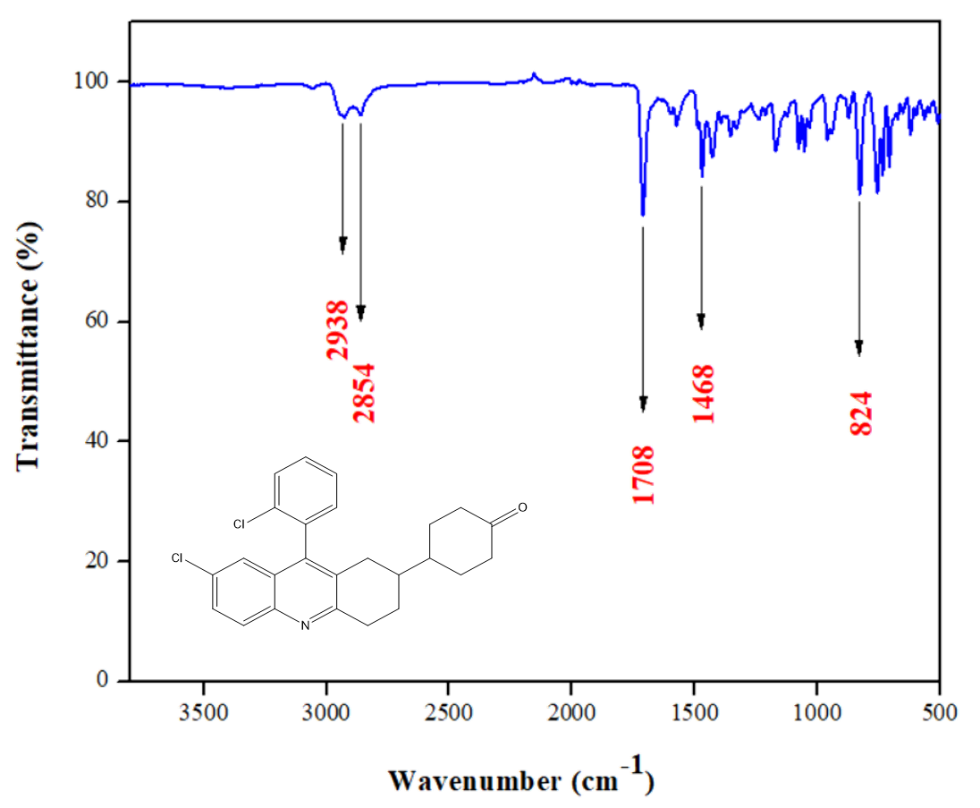


**Figure S11. FTIR spectrum of 4-(7-chloro-9-(2-chlorophenyl)-1,2,3,4-tetrahydroacridin-2-yl)cyclohexan-1-one (3c)**


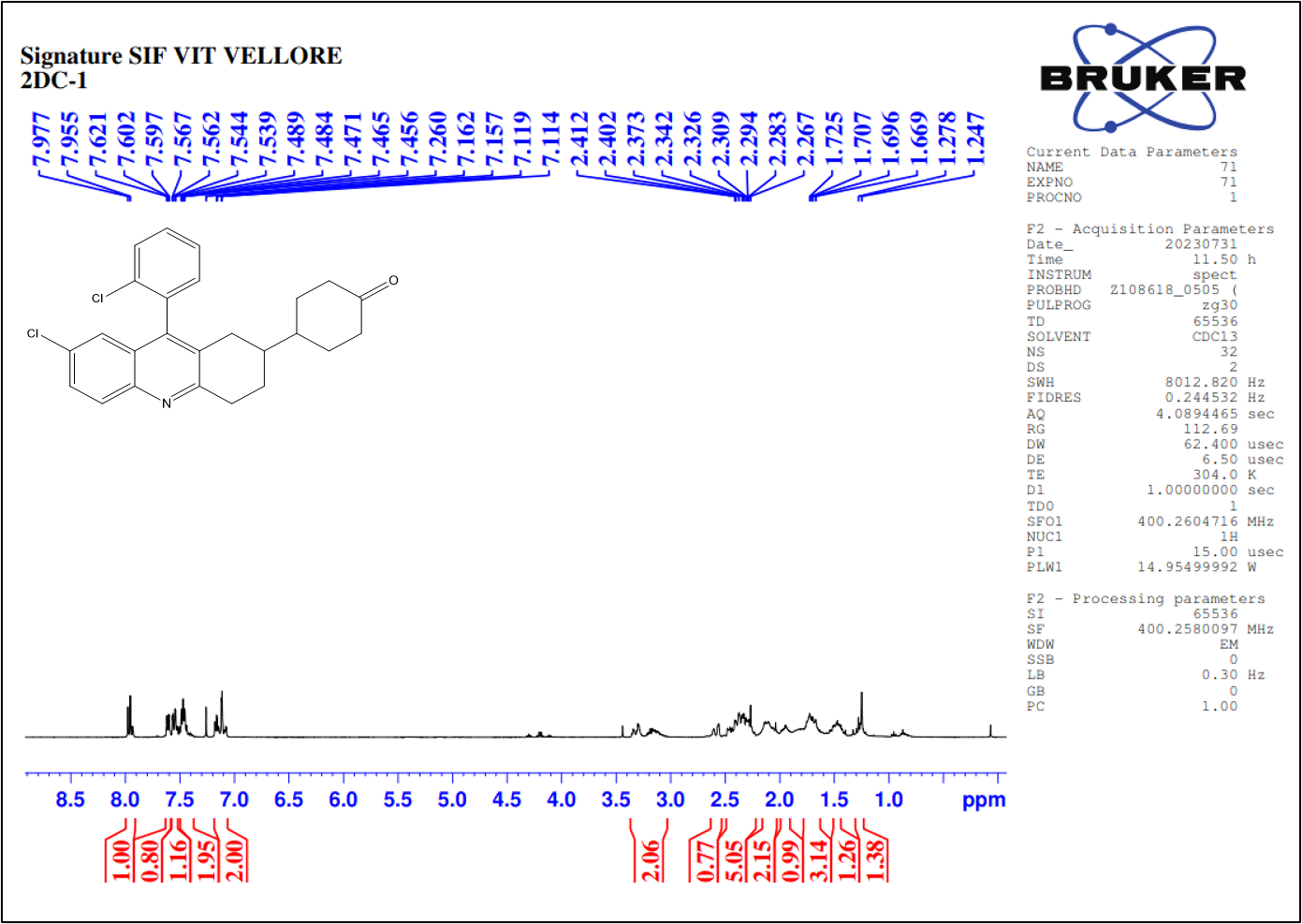


**Figure S12. ^1^H- NMR spectrum of 4-(7-chloro-9-(2-chlorophenyl)-1,2,3,4-tetrahydroacridin -2-yl)cyclohexan-1-one (3c)**

**
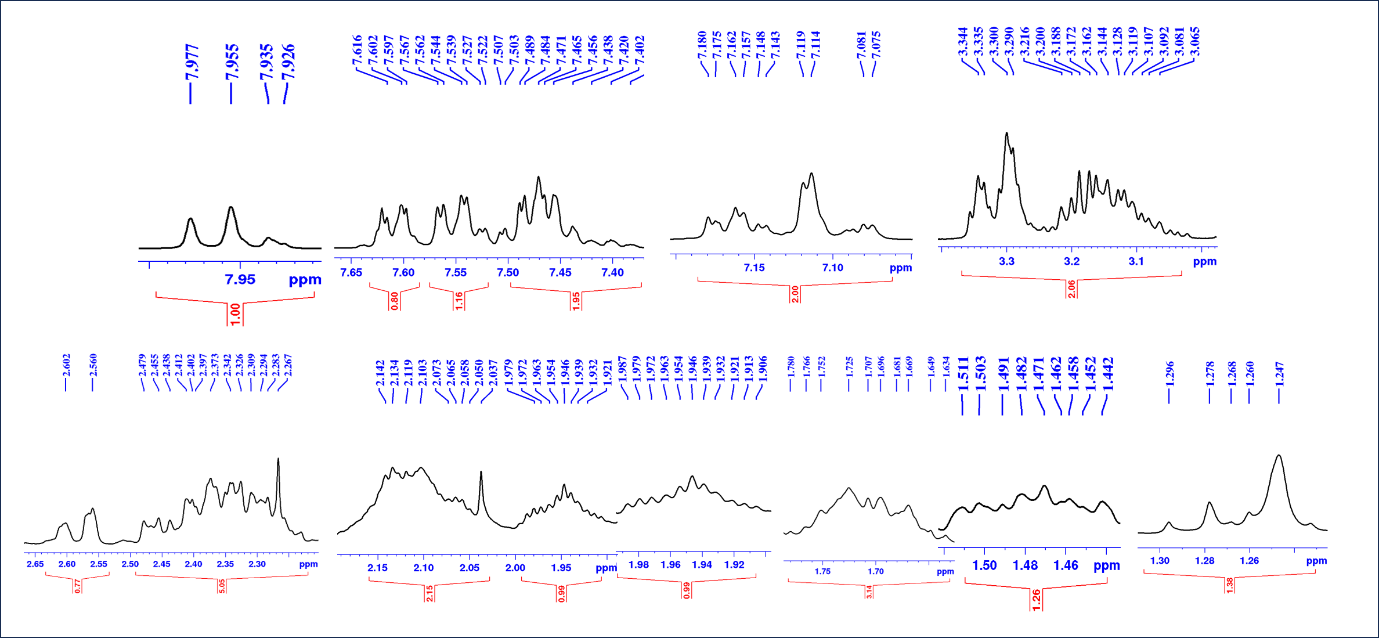
**

**Figure S13. Enlarged ^1^H- NMR spectrum of 4-(7-chloro-9-(2-chlorophenyl)-1,2,3,4 tetrahydroacridin-2-yl)cyclohexan-1-one (3c)**


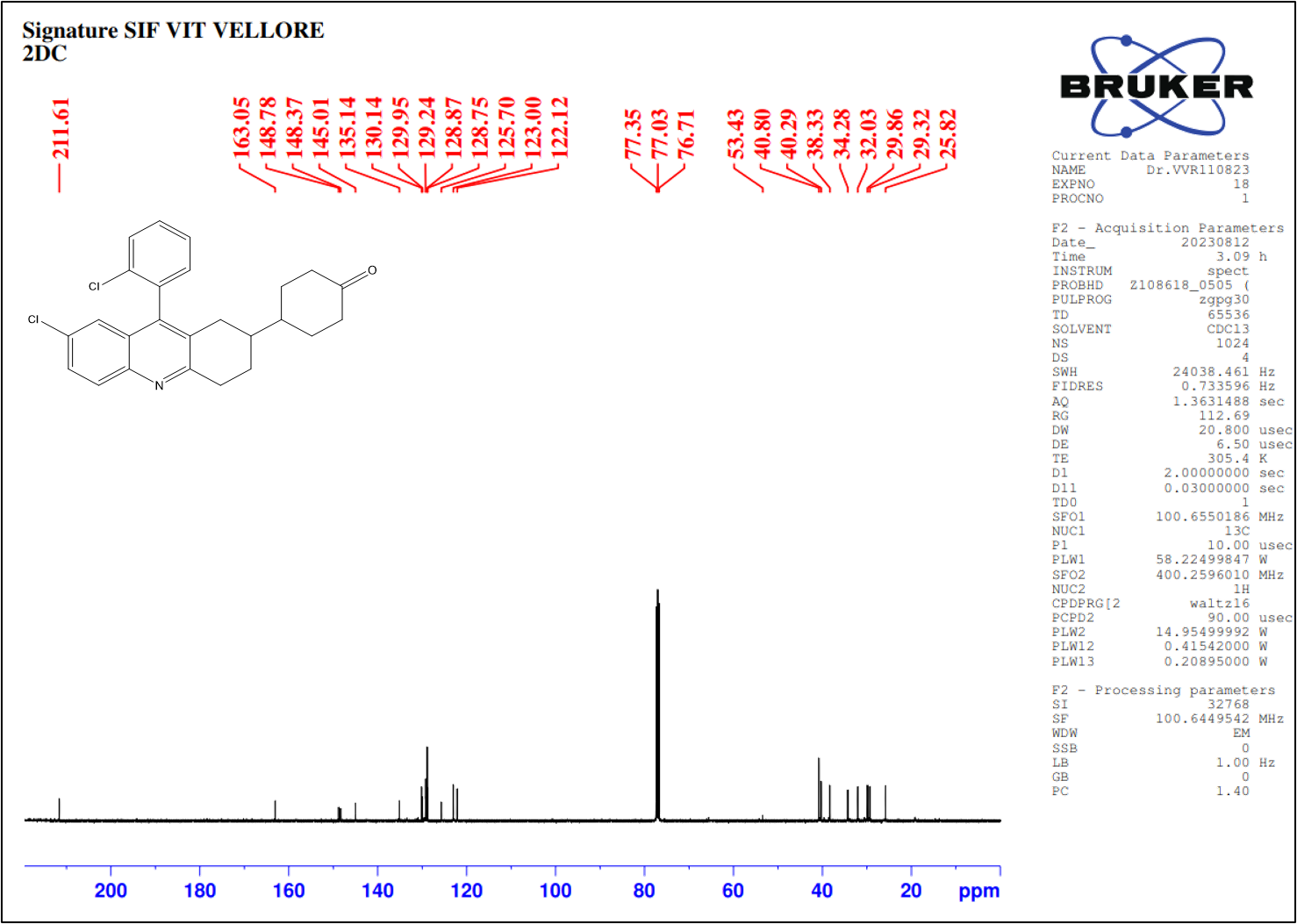


**Figure S14. ^13^C- NMR spectrum of 4-(7-chloro-9-(2-chlorophenyl)-1,2,3,4-tetrahydroacridin-2-yl)cyclohexan-1-one (3c)**


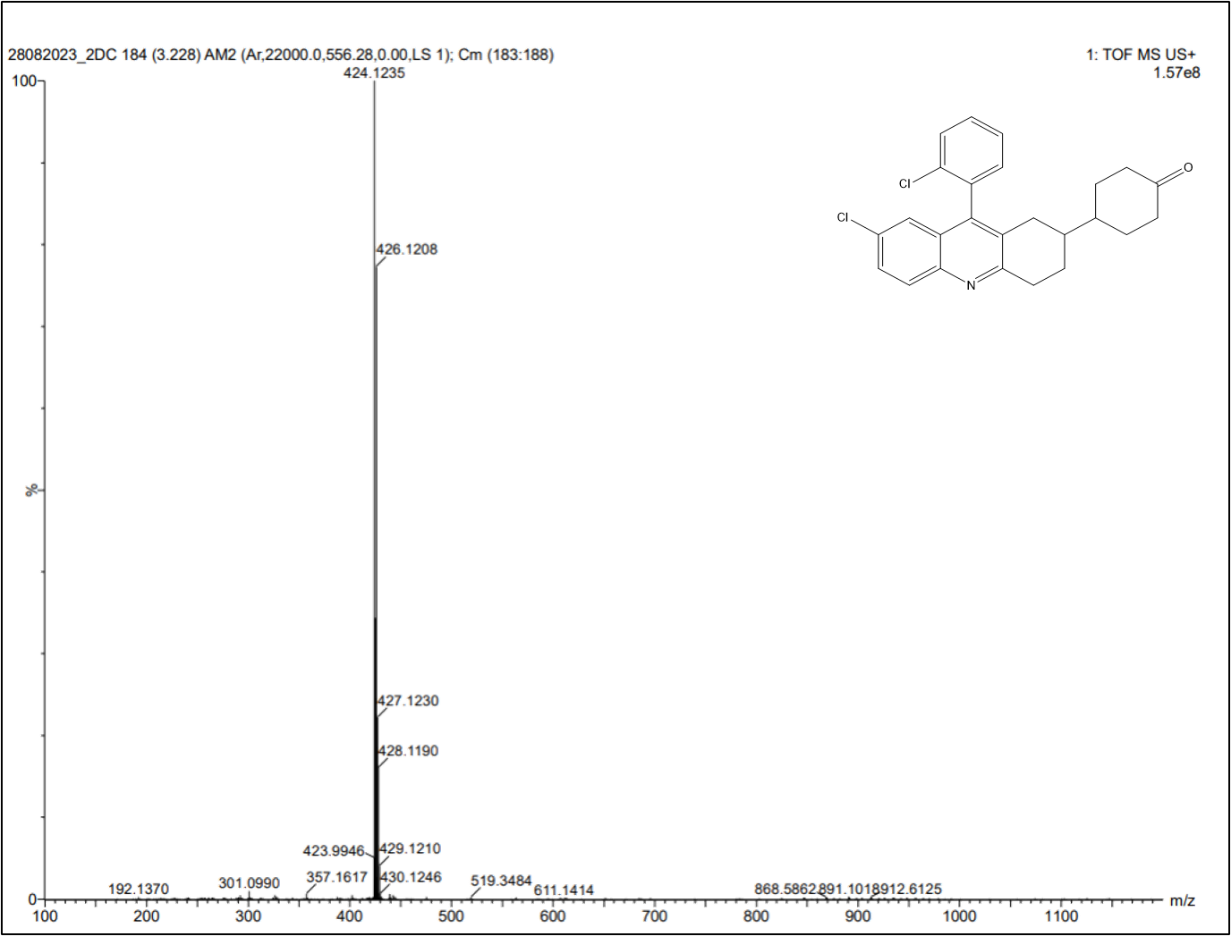


**Figure S15. HRMS spectrum of 4-(7-chloro-9-(2-chlorophenyl)-1,2,3,4-tetrahydroacridin-2-yl)cyclohexan-1-one (3c)**


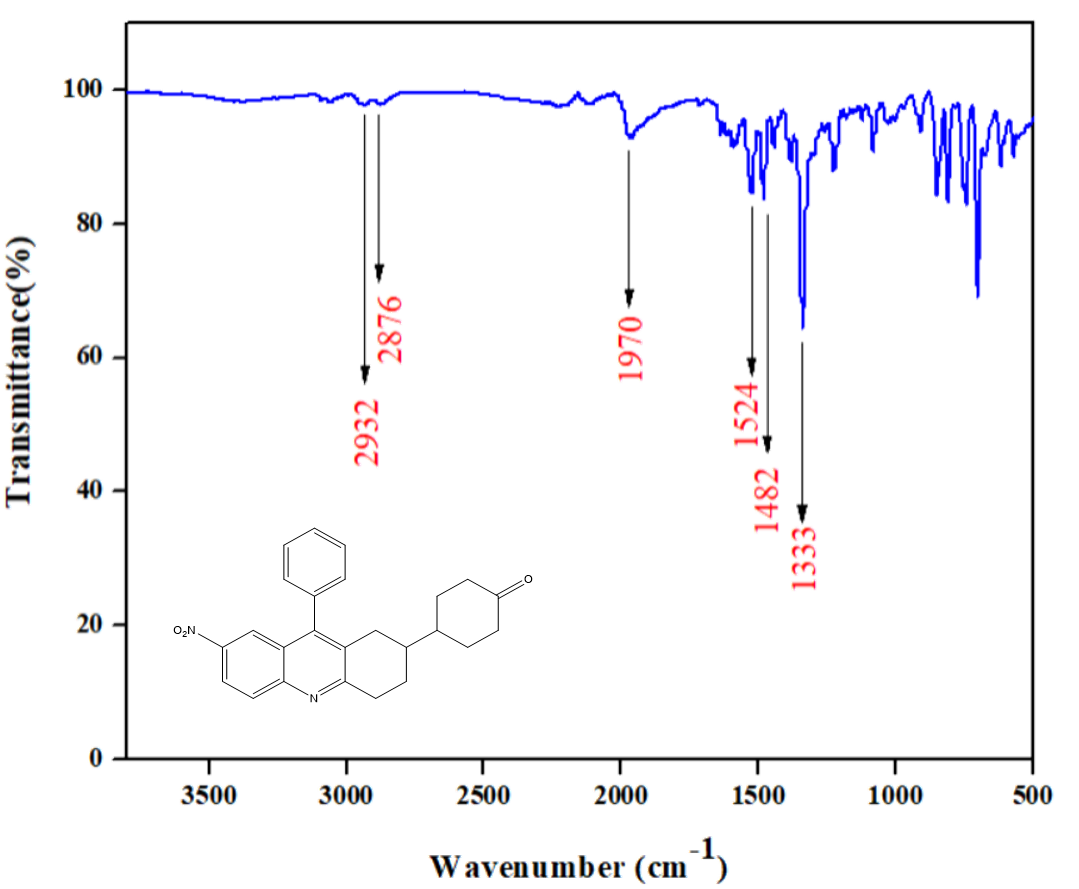


**Figure S16. FTIR spectrum of 4-(7-nitro-9-phenyl-1,2,3,4-tetrahydroacridin-2-yl)cyclohexan-1-one (3d)**


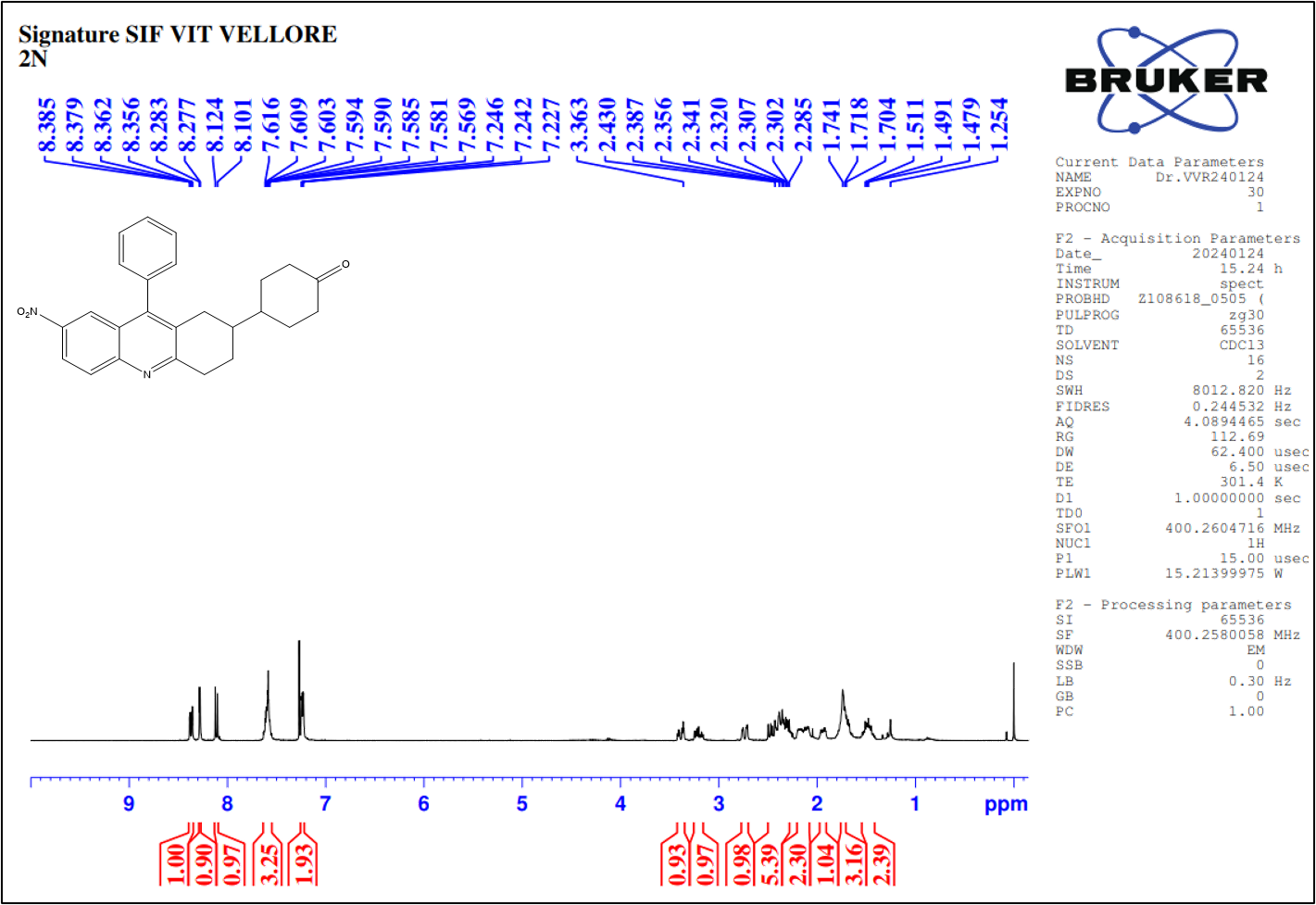


**Figure S17. ^1^H- NMR spectrum of 4-(7-nitro-9-phenyl-1,2,3,4-tetrahydroacridin-2-yl)cyclohexan-1-one (3d)**


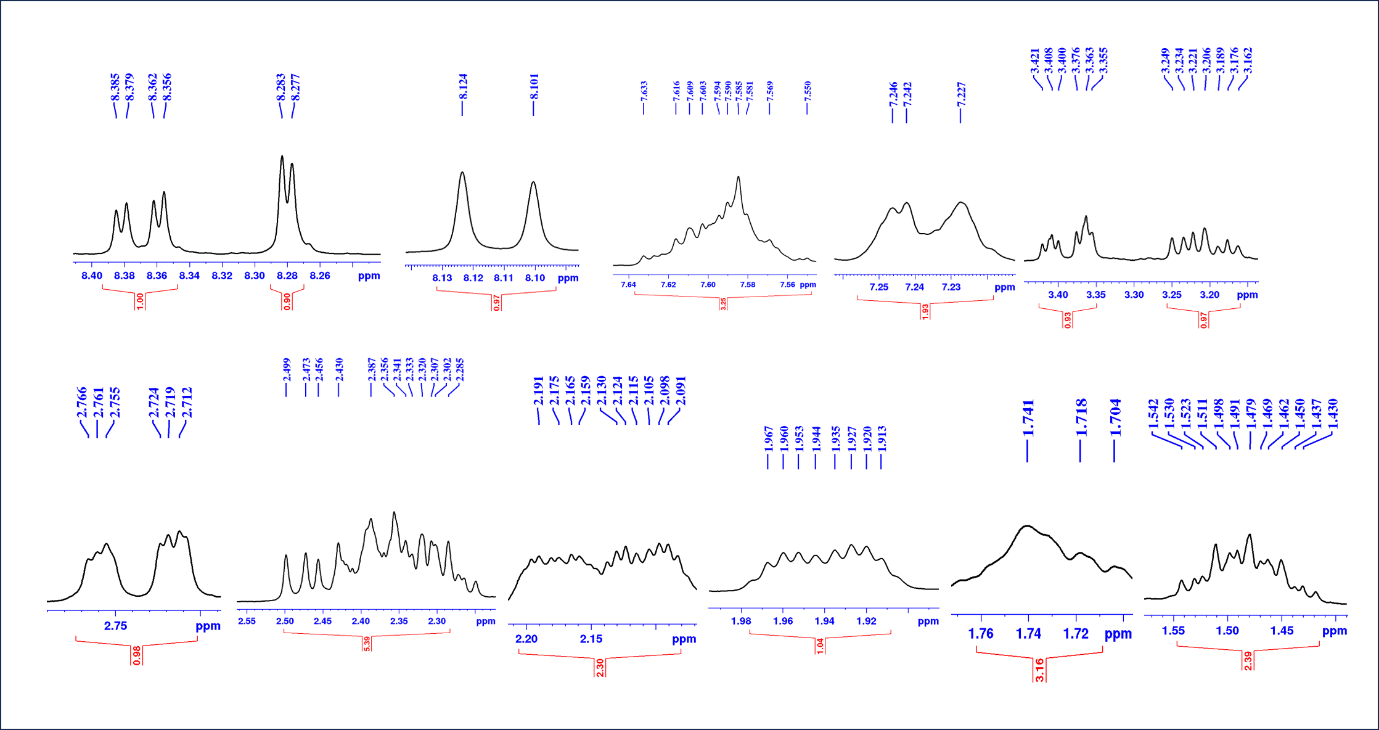


**Figure S18.Enlarged ^1^H- NMR spectrum of 4-(7-nitro-9-phenyl-1,2,3,4-tetrahydroacridin-2-yl)cyclohexan-1-one (3d)**


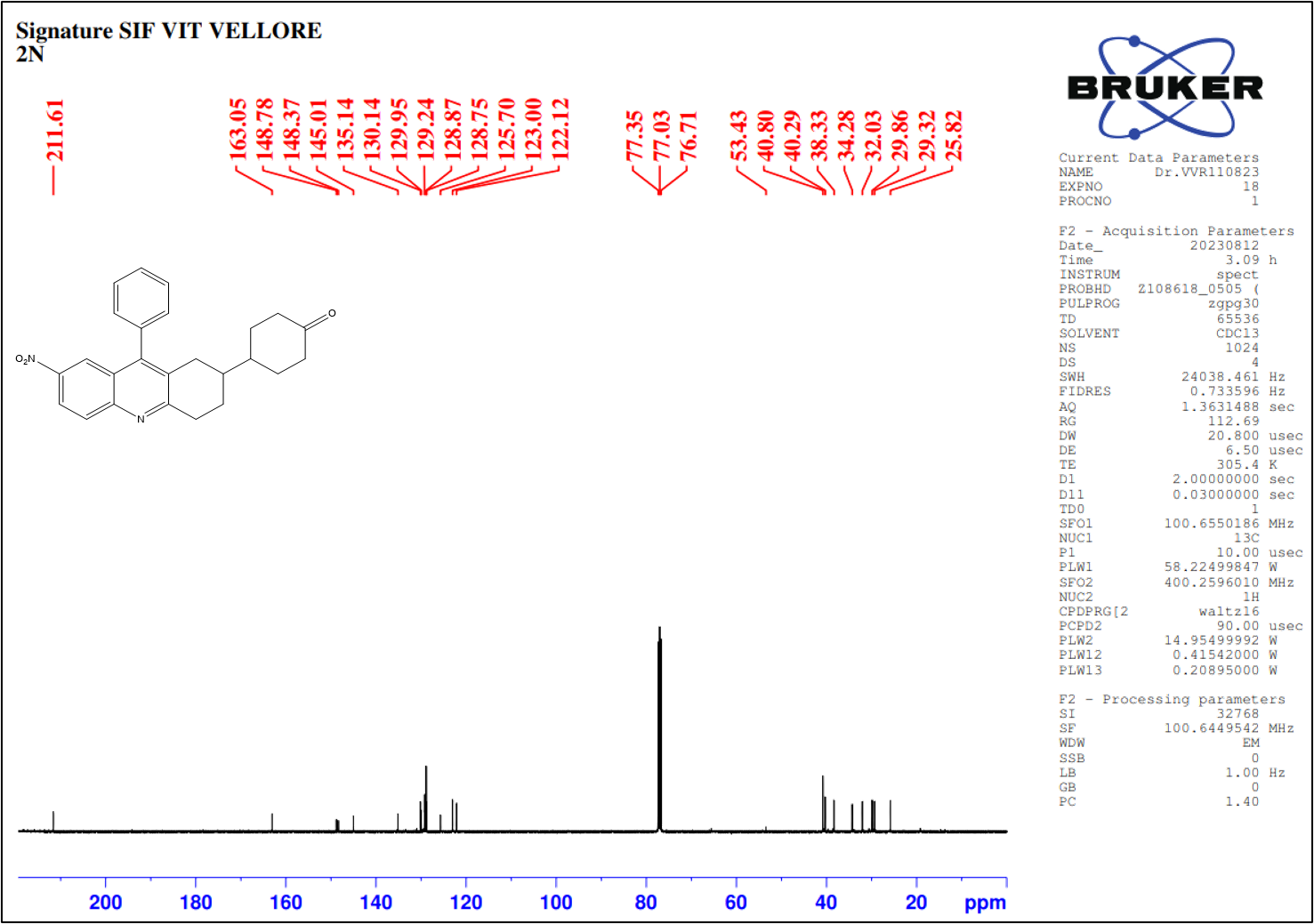


**Figure S19. ^13^C- NMR spectrum of 4-(7-nitro-9-phenyl-1,2,3,4-tetrahydroacridin-2-yl)cyclohexan-1-one (3d)**


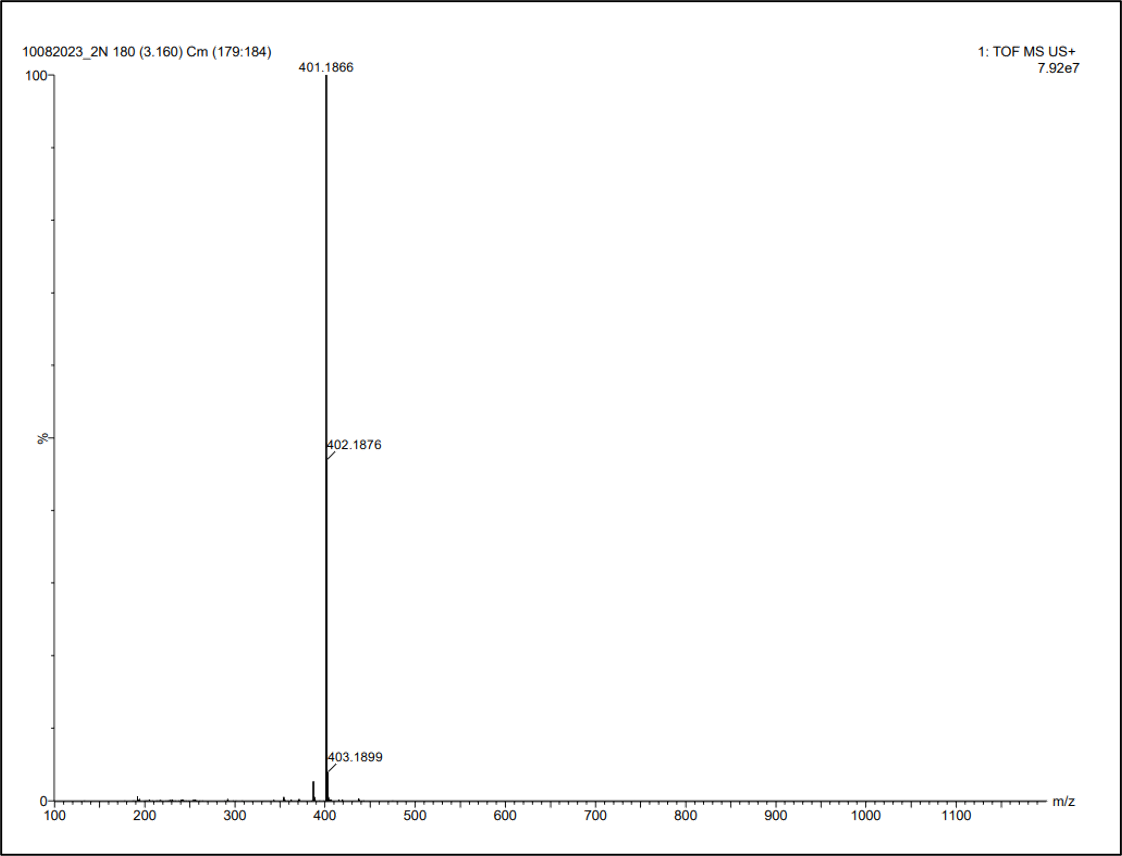

**Figure S20. HRMS spectrum of 4-(7-nitro-9-phenyl-1,2,3,4-tetrahydroacridin-2-yl)cyclohexan-1-one (3d)**


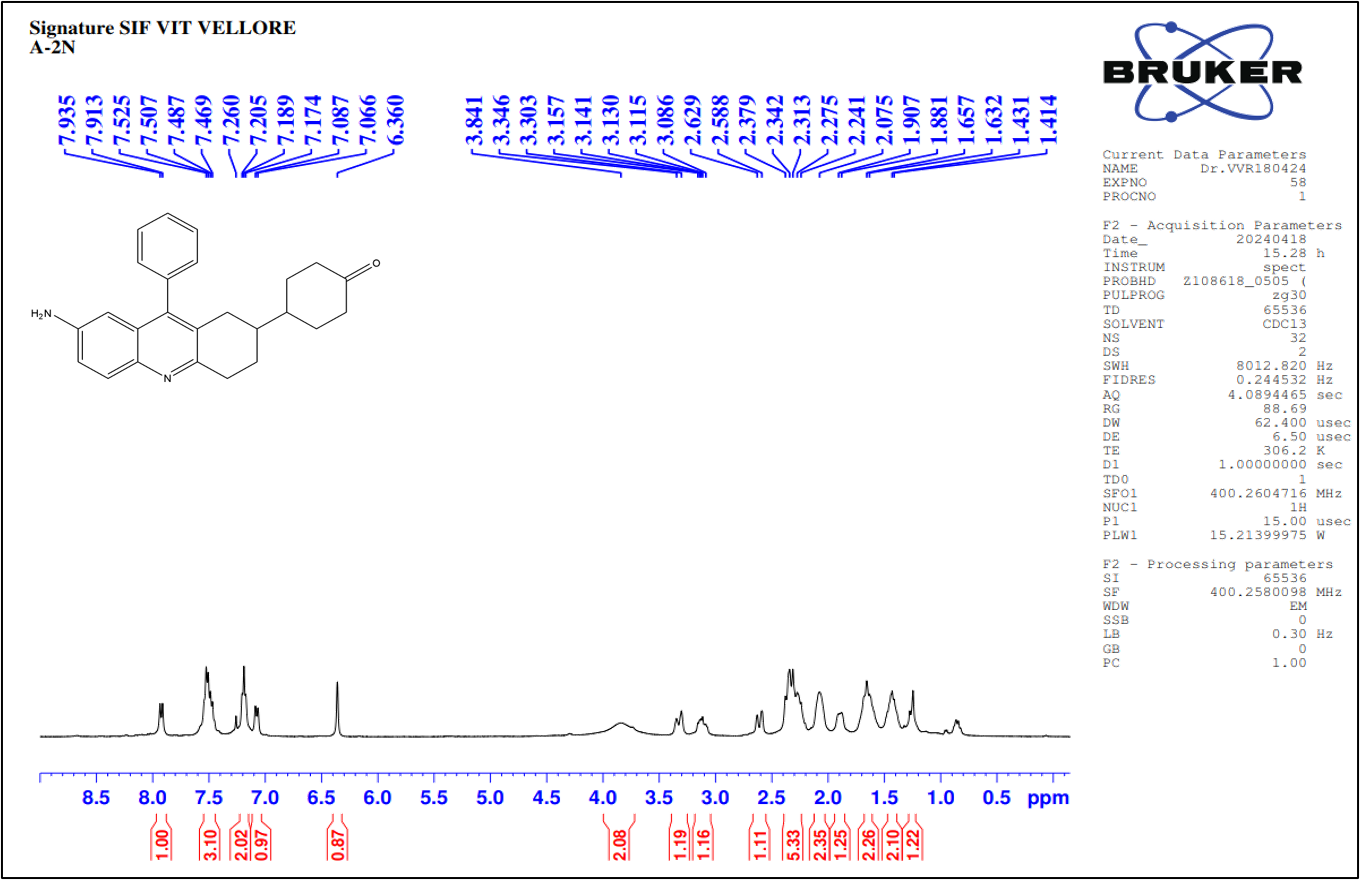


**Figure S21. ^1^H- NMR spectrum of 4-(7-amino -9-phenyl-1,2,3,4-tetrahydroacridin-2-yl)cyclohexan-1-one) (3e)**


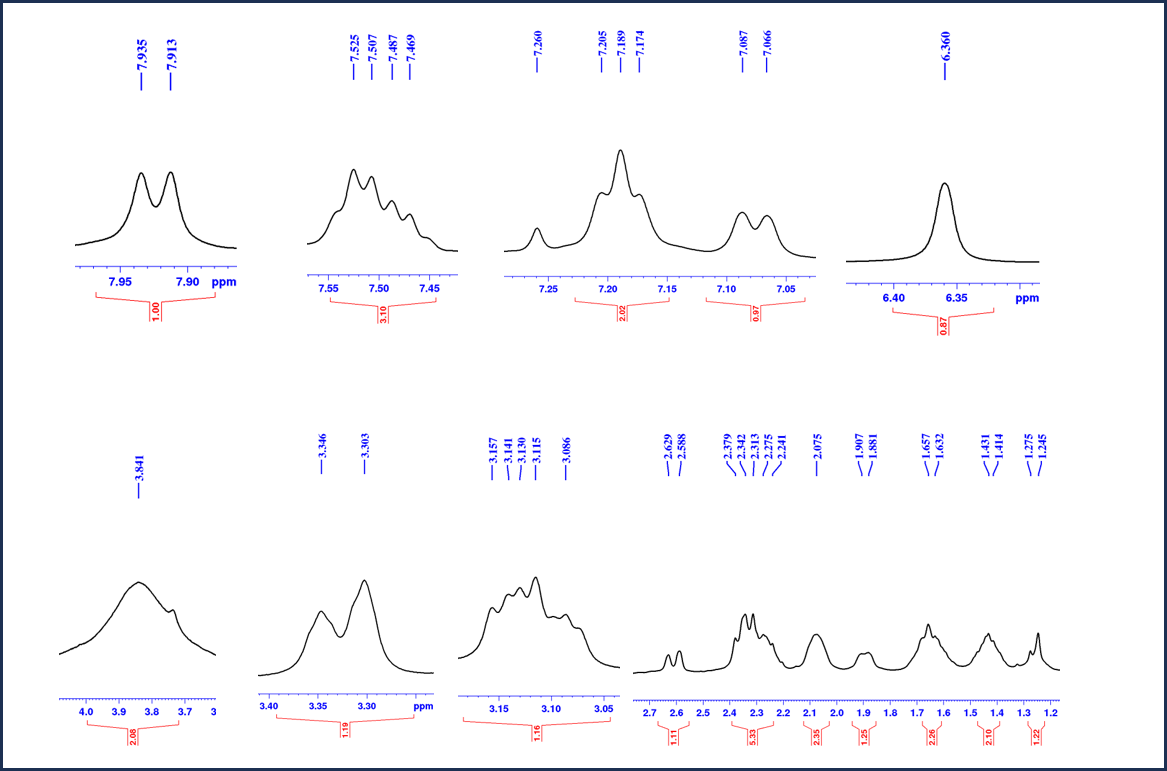


**Figure S22.Enlarged ^1^H- NMR spectrum of 4-(7-amino -9-phenyl-1,2,3,4-tetrahydroacridin-2-yl)cyclohexan-1-one) (3e)**


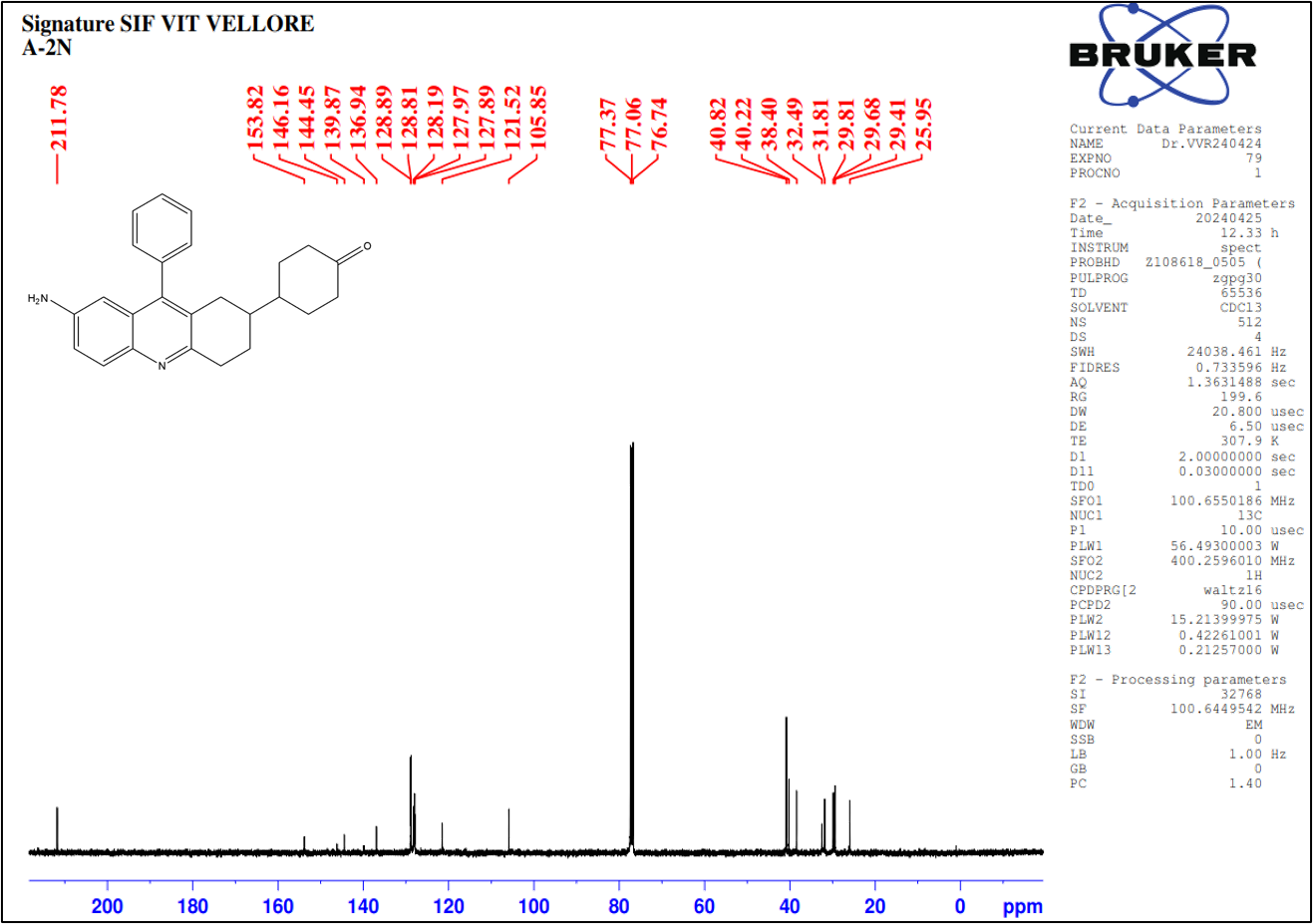


**Figure S23. ^13^C- NMR spectrum of 4-(7-amino -9-phenyl-1,2,3,4-tetrahydroacridin-2-yl)cyclohexan-1-one) (3e)**

**
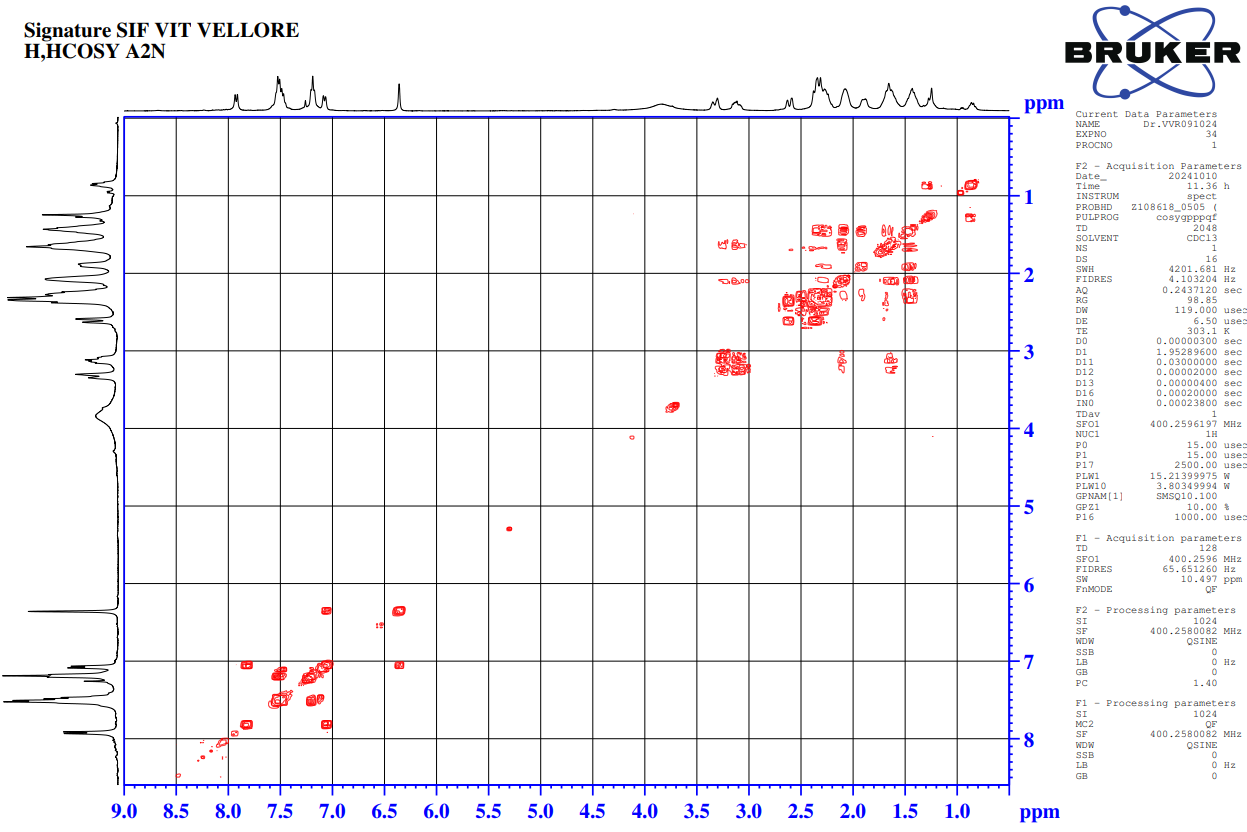
**

**Figure S24. H,H COSY spectrum of 4-(7-amino -9-phenyl-1,2,3,4-tetrahydroacridin-2-yl)cyclohexan-1-one (3e)**

**
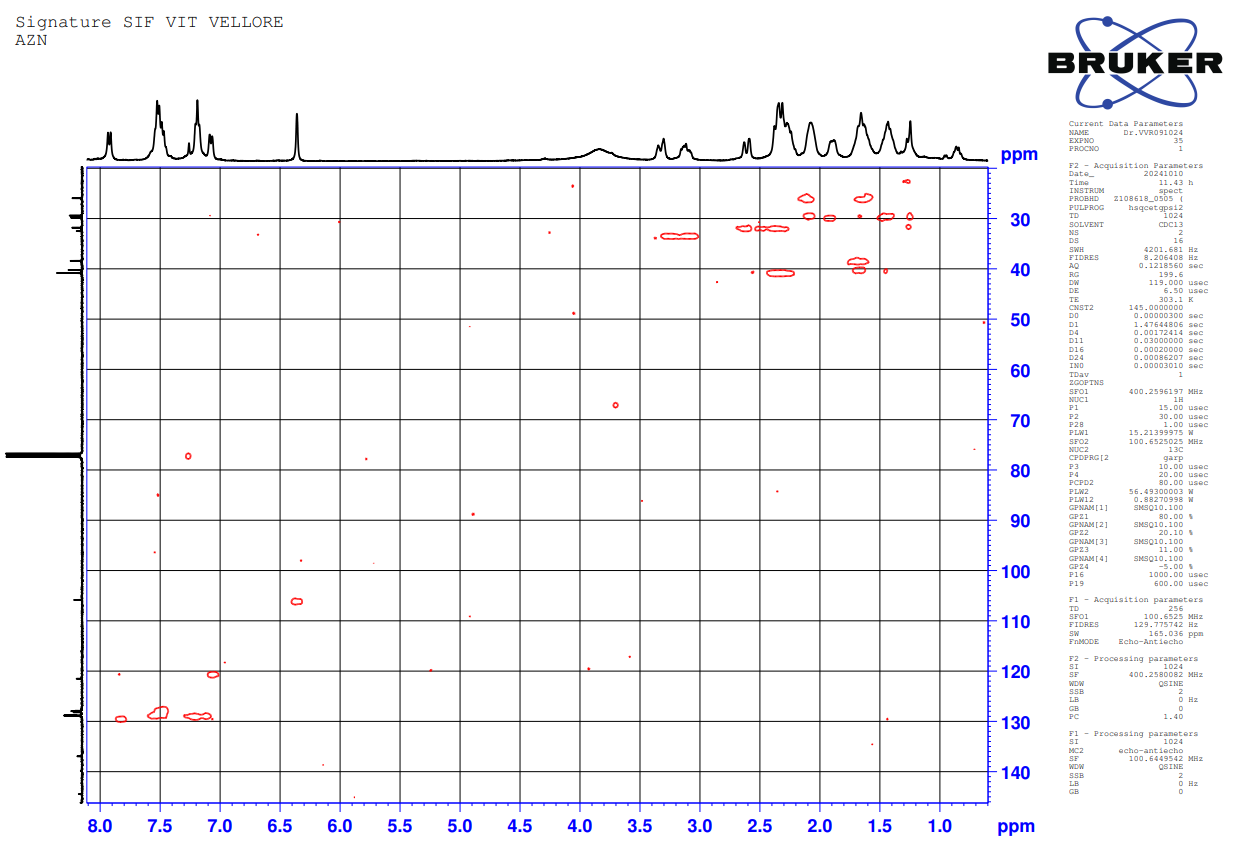
**

**Figure S25. HSQC spectrum of 4-(7-amino -9-phenyl-1,2,3,4-tetrahydroacridin-2-yl)cyclohexan-1-one) (3e)**


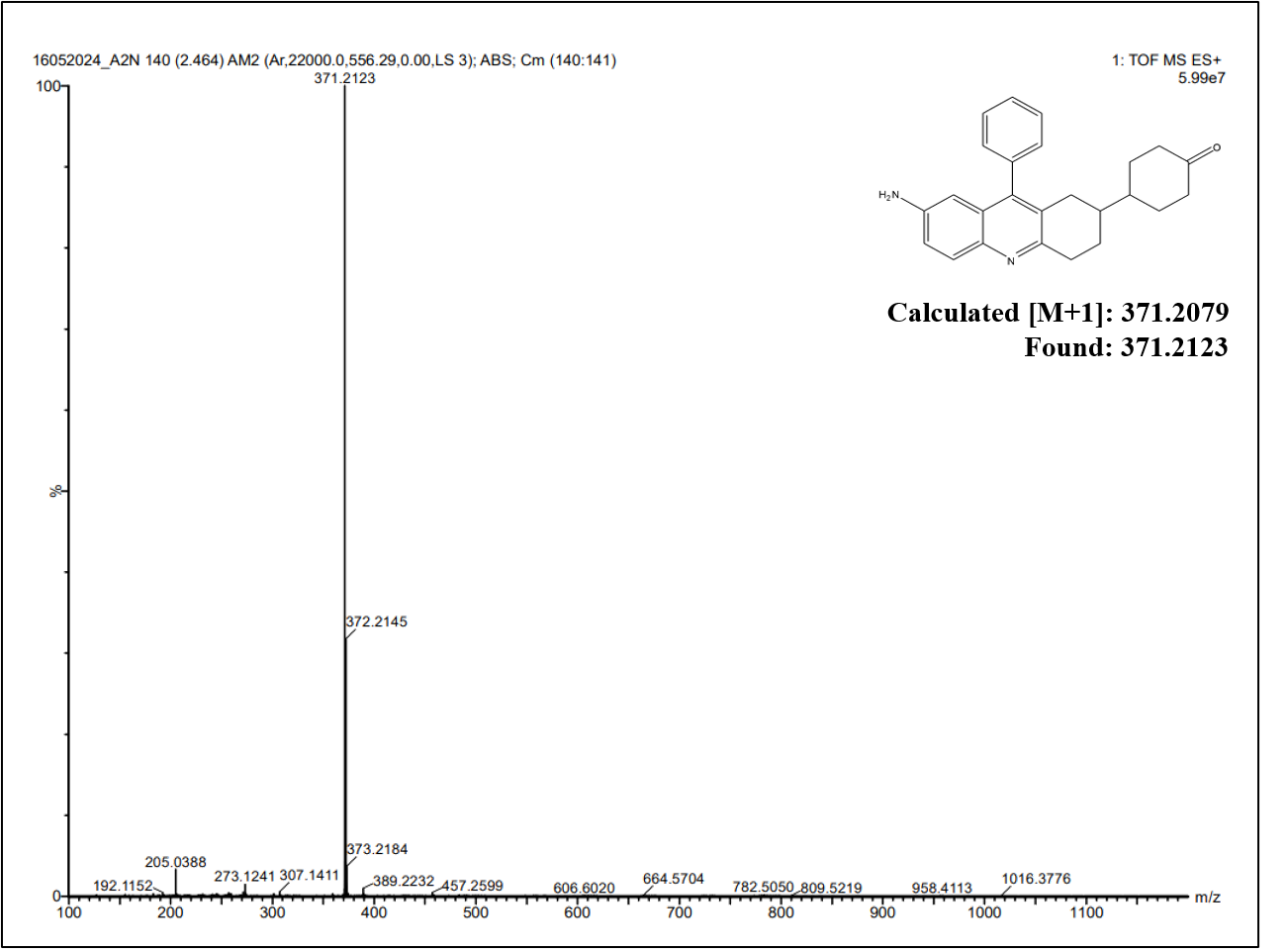


**Figure S26. HRMS spectrum of 4-(7-amino-9-phenyl-1,2,3,4-tetrahydroacridin-2-yl)cyclohexan-1-one (3e)**


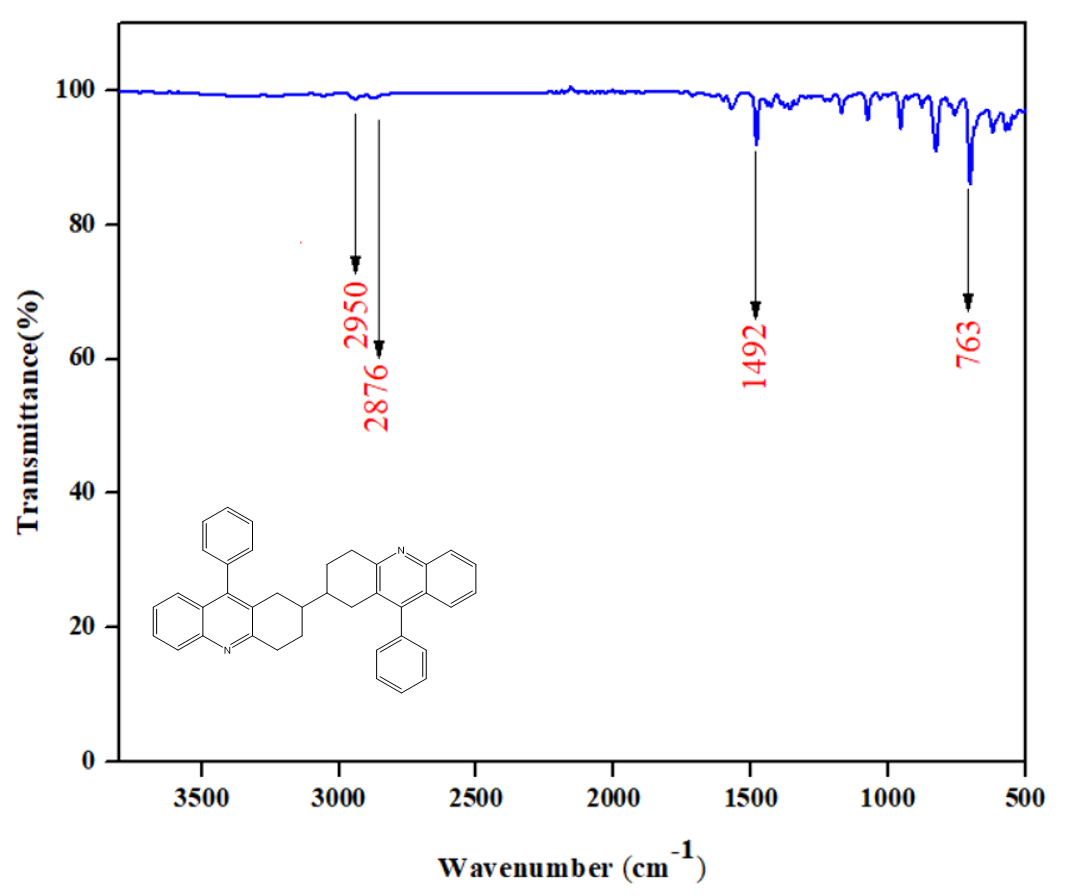


**Figure S27. FTIR spectrum of 9,9'-diphenyl-1,1',2,2',3,3',4,4'-octahydro-2,2'-biacridine (4a)**


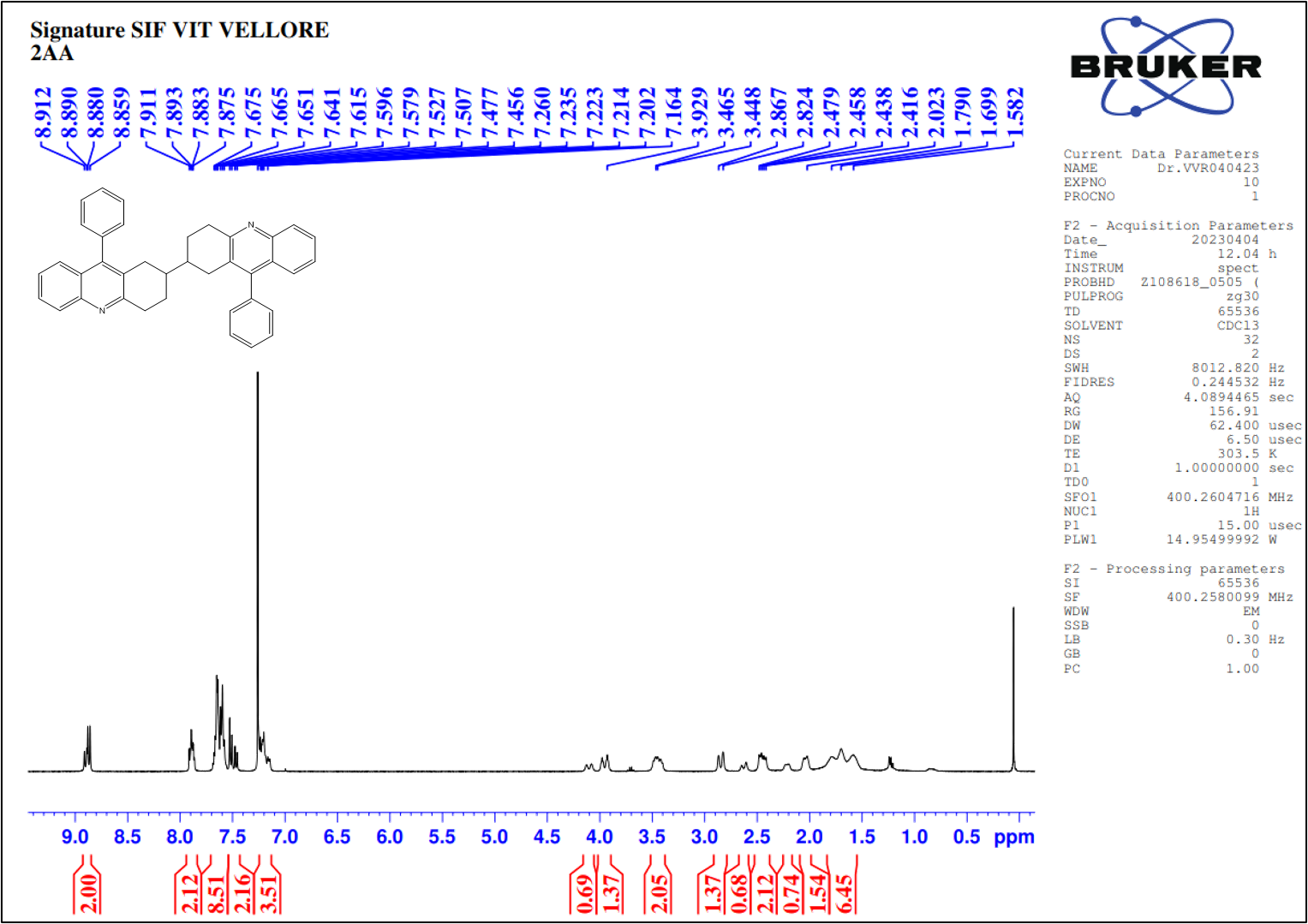


**Figure S28. ^1^H- NMR spectrum of 9,9'-diphenyl-1,1',2,2',3,3',4,4'-octahydro-2,2'-biacridine (4a)**

**
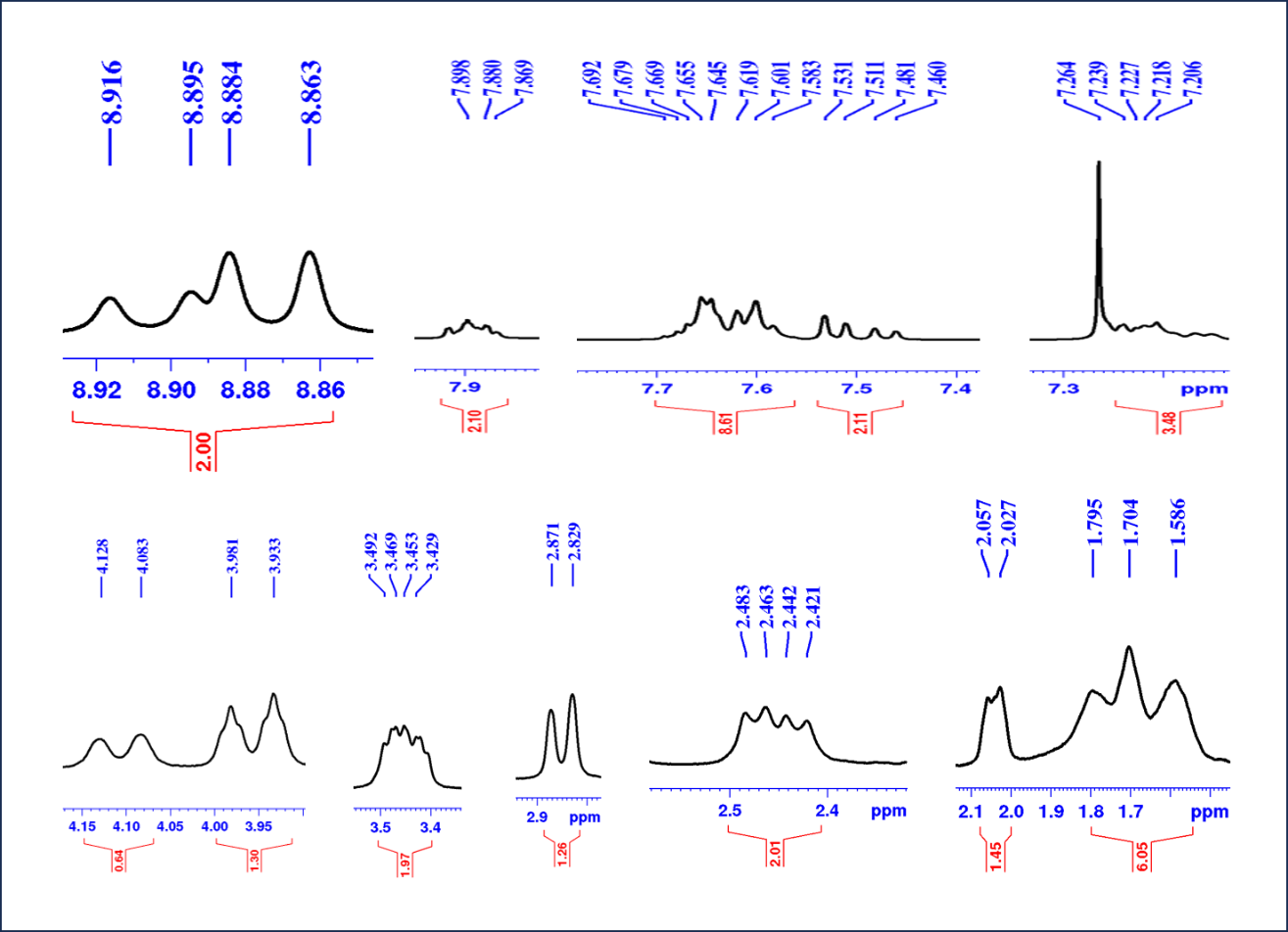
**

**Figure S29. Enlarged ^1^H- NMR spectrum of 9,9'-diphenyl-1,1',2,2',3,3',4,4'-octahydro-2,2'-biacridine (4a)**


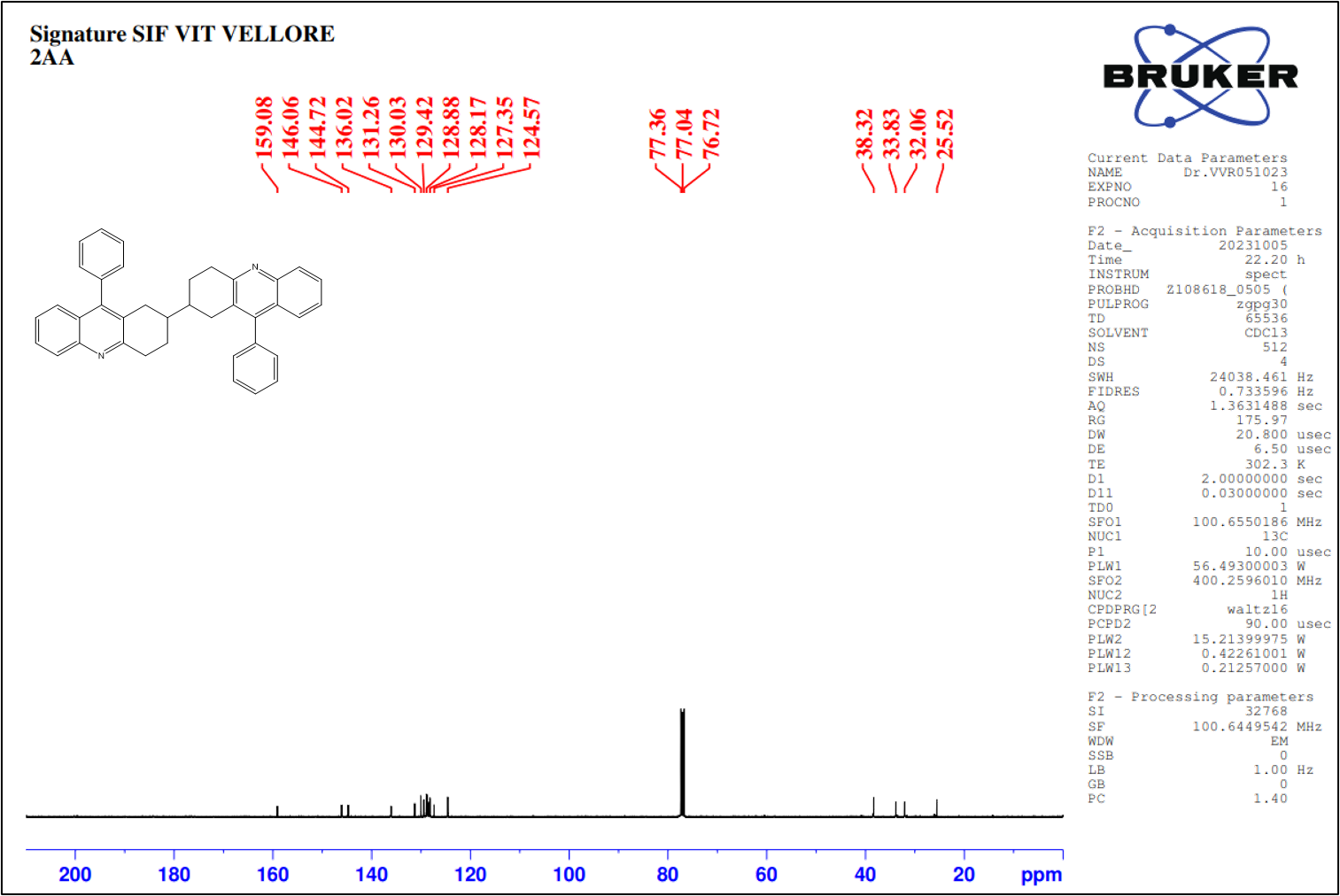


**Figure S30. ^13^C NMR spectrum of 9,9'-diphenyl-1,1',2,2',3,3',4,4'-octahydro-2,2'-biacridine (4a)**


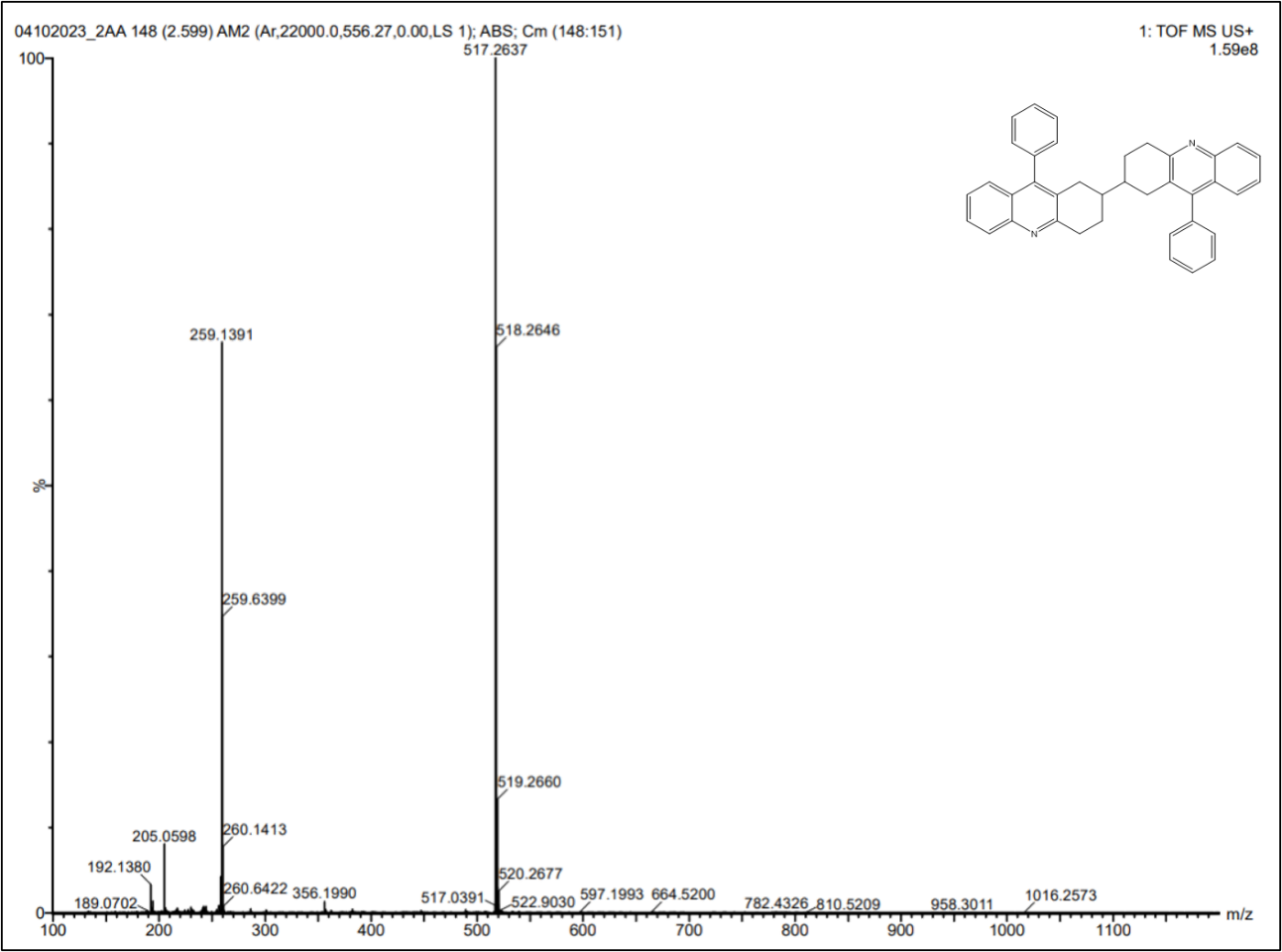


**Figure S31. HRMS spectrum of 9,9'-diphenyl-1,1',2,2',3,3',4,4'-octahydro-2,2'-biacridine (4a)**


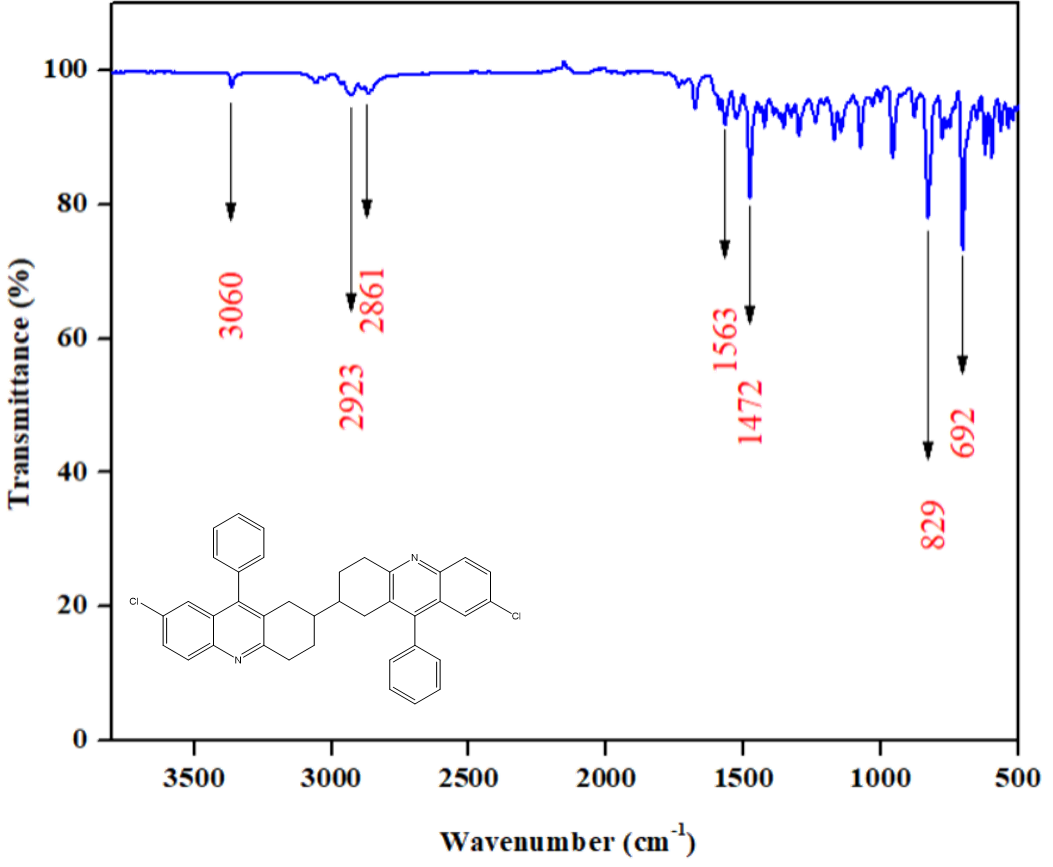


**Figure S32. FTIR spectrum of 7,7'-dichloro-9,9'-diphenyl-1,1',2,2',3,3',4,4'-octahydro-2,2'-biacridine (4b)**


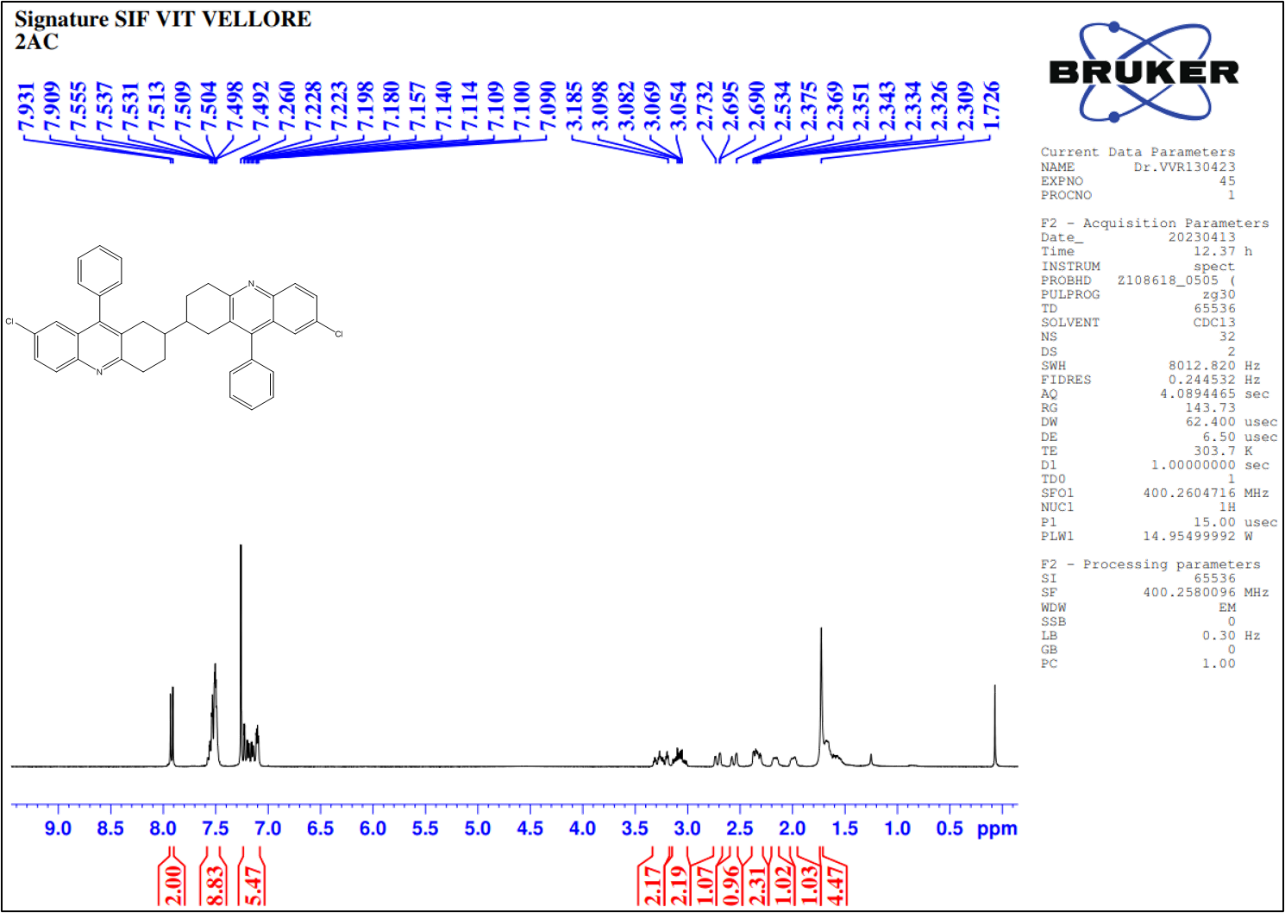


**Figure S33. ^1^H NMR spectrum of 7,7'-dichloro-9,9'-diphenyl-1,1',2,2',3,3',4,4'-octahydro-2,2'-biacridine (4b)**

**
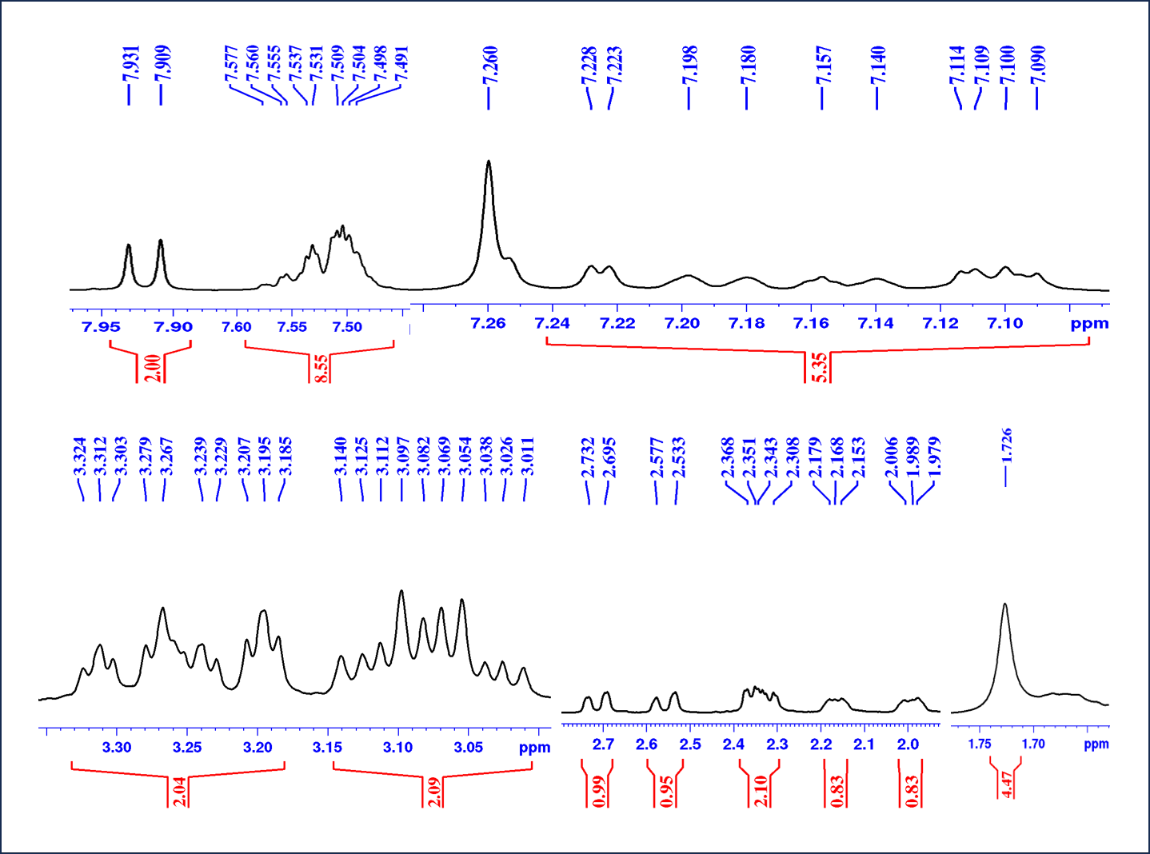
**

**Figure S34. Enlarged ^1^H NMR spectrum of 7,7'-dichloro-9,9'-diphenyl-1,1',2,2',3,3',4,4'-octahydro-2,2'-biacridine (4b)**

^
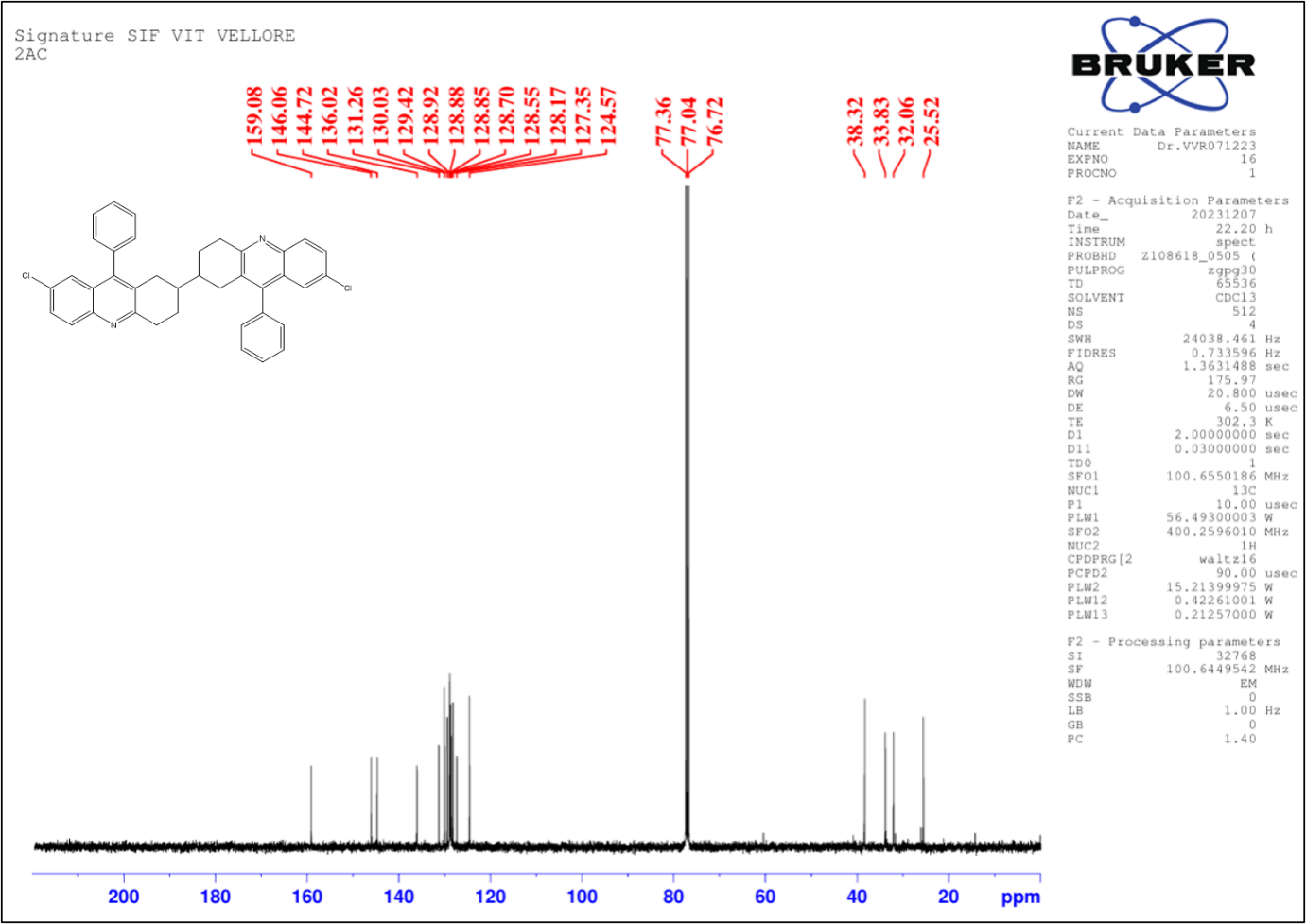
^

**Figure S35. ^13^C- NMR spectrum of 7,7'-dichloro-9,9'-diphenyl-1,1',2,2',3,3',4,4'-octahydro-2,2'-biacridine (4b)**


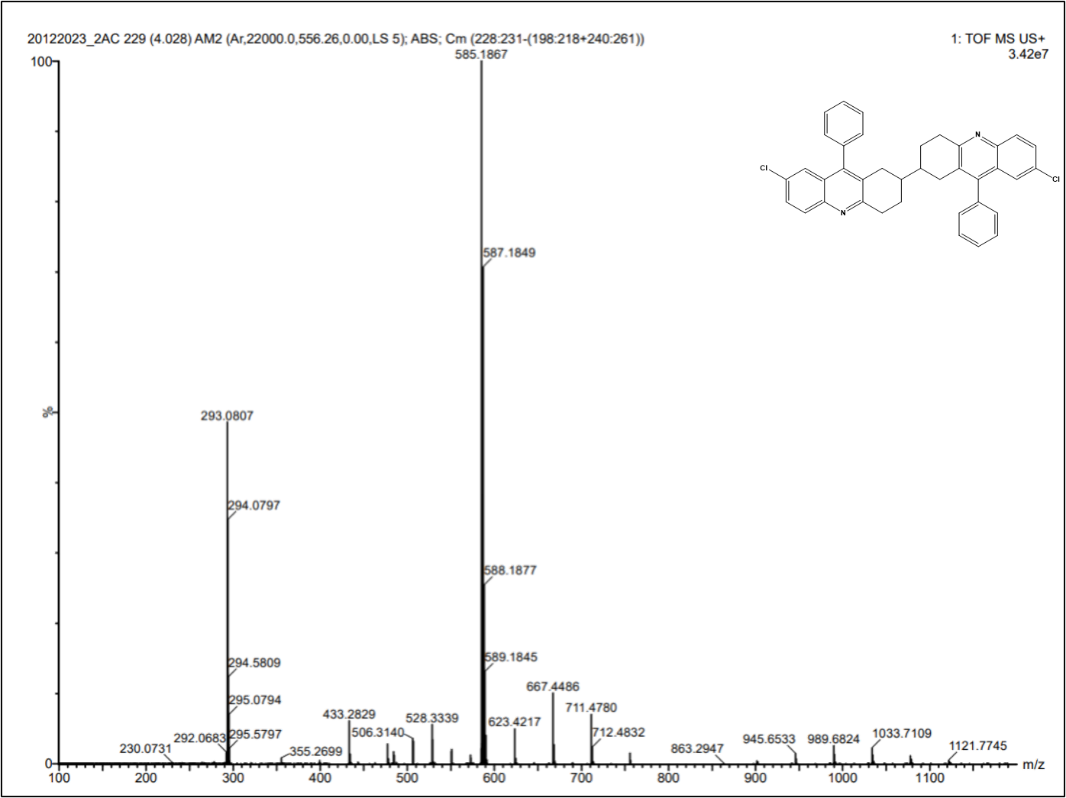


**Figure S36. HRMS spectrum of 7,7'-dichloro-9,9'-diphenyl-1,1',2,2',3,3',4,4'-octahydro-2,2'-biacridine (4b)**


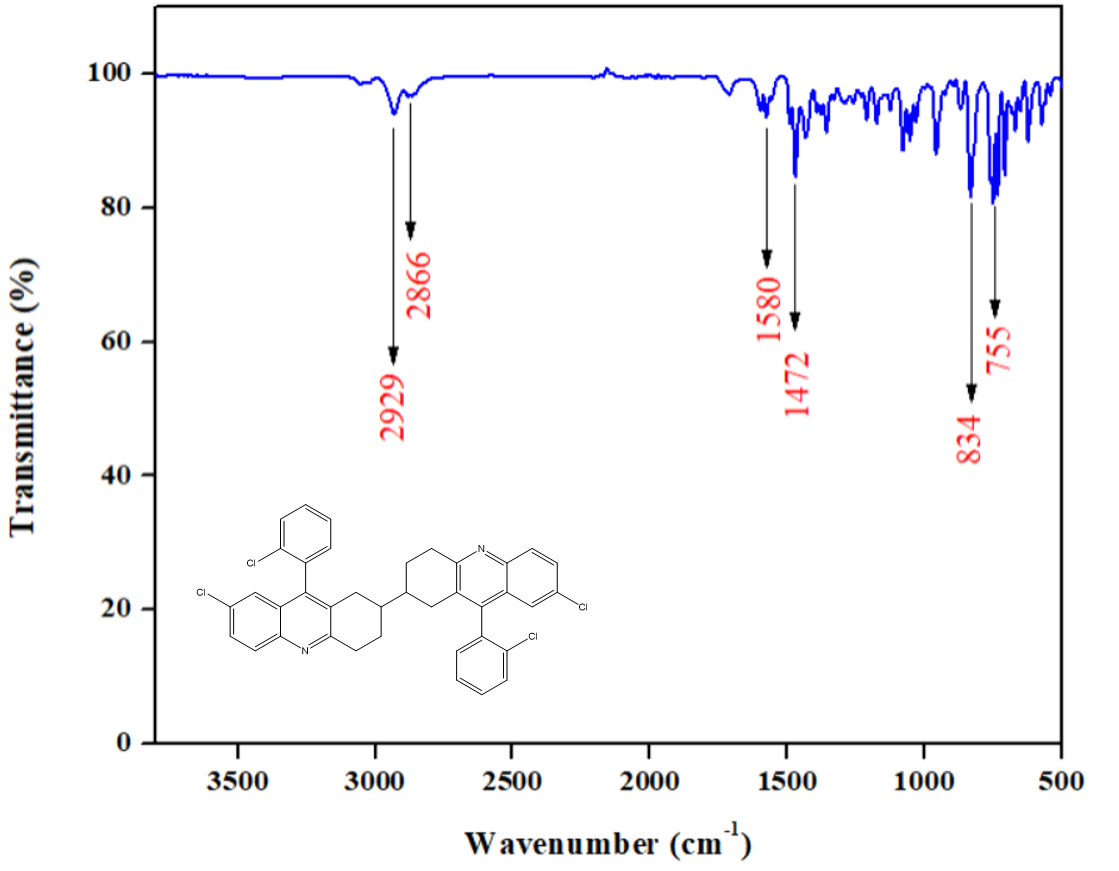


**Figure S37. FTIR spectrum of 7,7'-dichloro-9,9'-bis(2-chlorophenyl)-1,1',2,2',3,3',4,4'-octahydro-2,2'-biacridine (4c)**


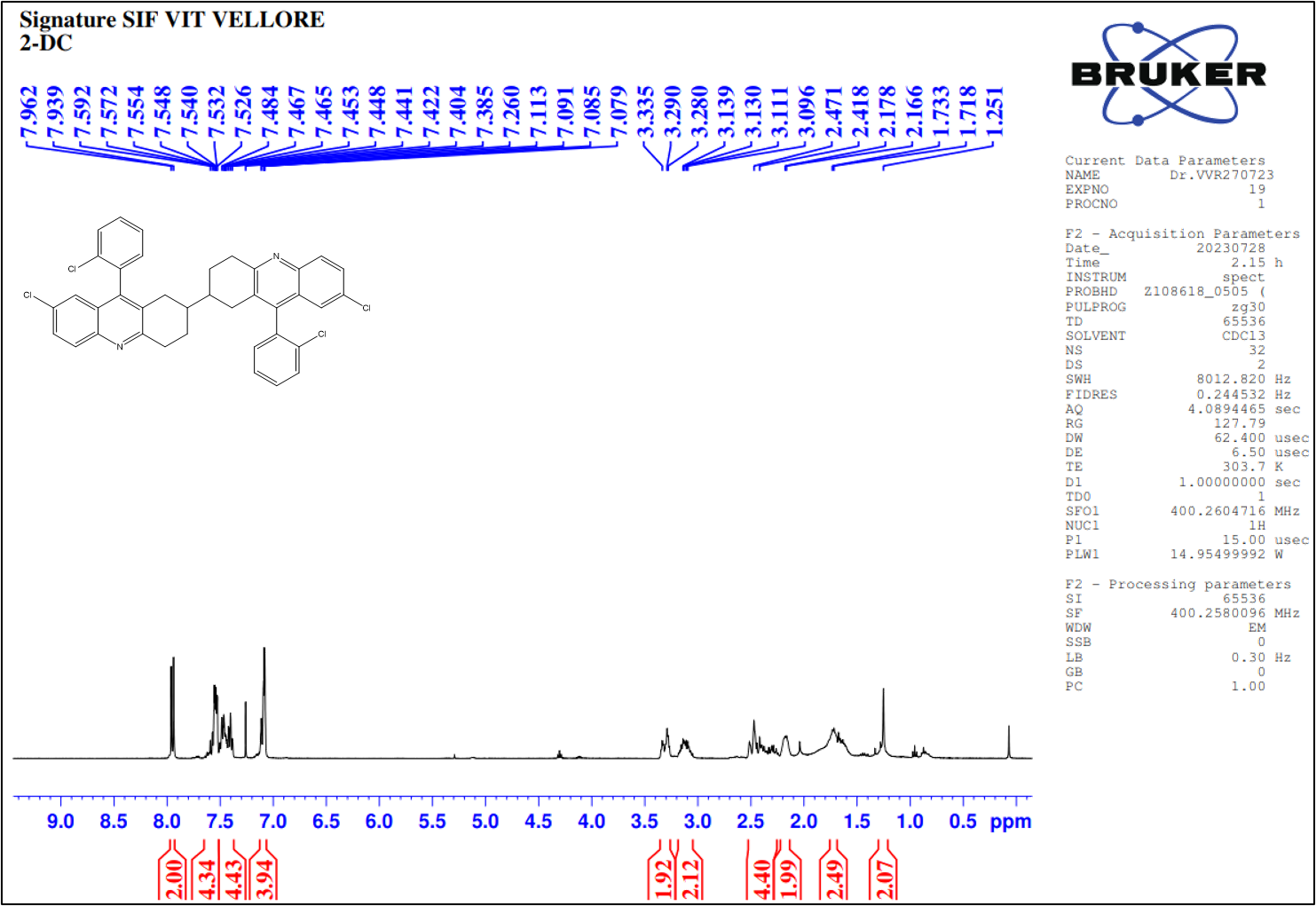


**Figure S38. ^1^H- NMR spectrum of7,7'-dichloro-9,9'-bis(2-chlorophenyl)-1,1',2,2',3,3',4,4'-octahydro-2,2'-biacridine (4c)**

**
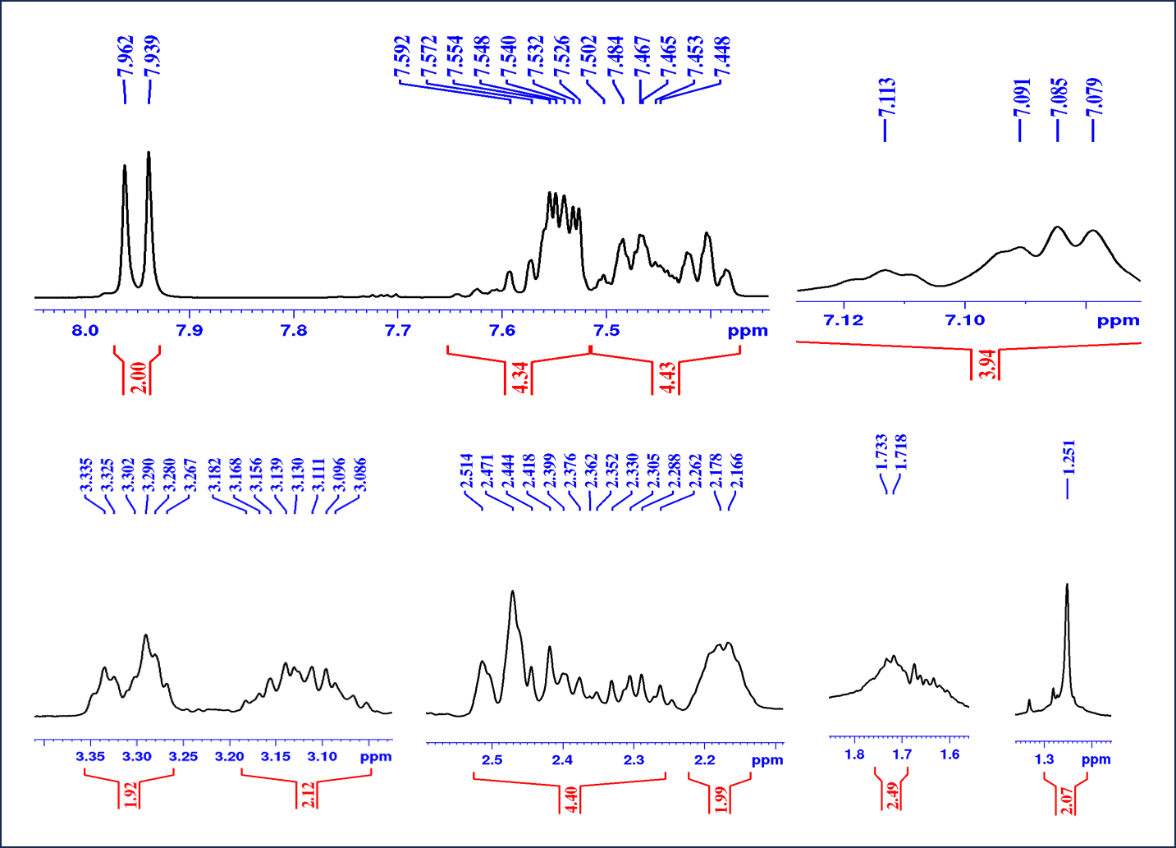
**

**Figure S39. Enlarged ^1^H- NMR spectrum of7,7'-dichloro-9,9'-bis(2-chlorophenyl)-1,1',2,2',3,3',4,4'-octahydro-2,2'-biacridine (4c)**


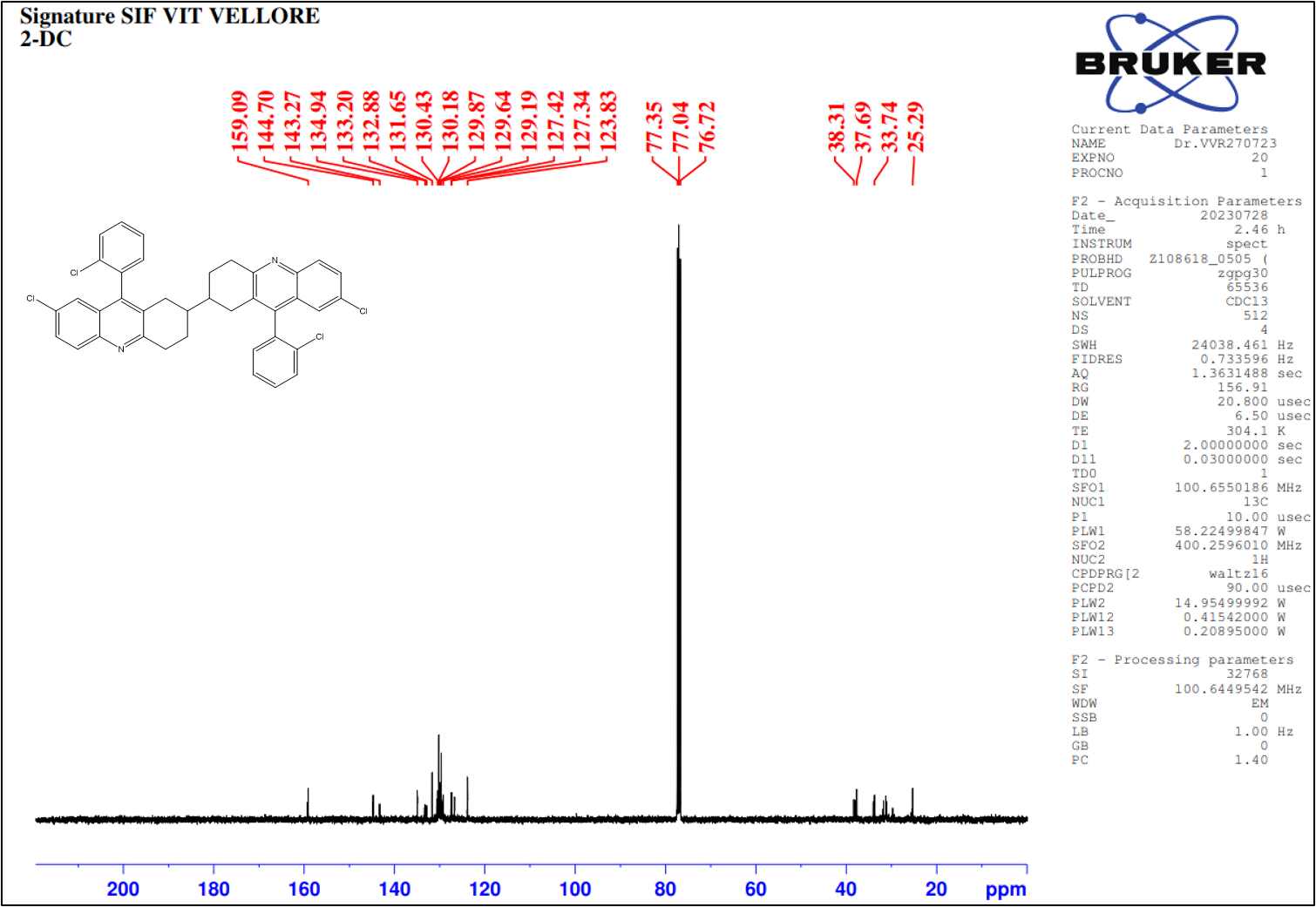


**Figure S40. ^13^C- NMR spectrum of 7,7'-dichloro-9,9'-bis(2-chlorophenyl)-1,1',2,2',3,3',4,4'-octahydro-2,2'-biacridine (4c)**


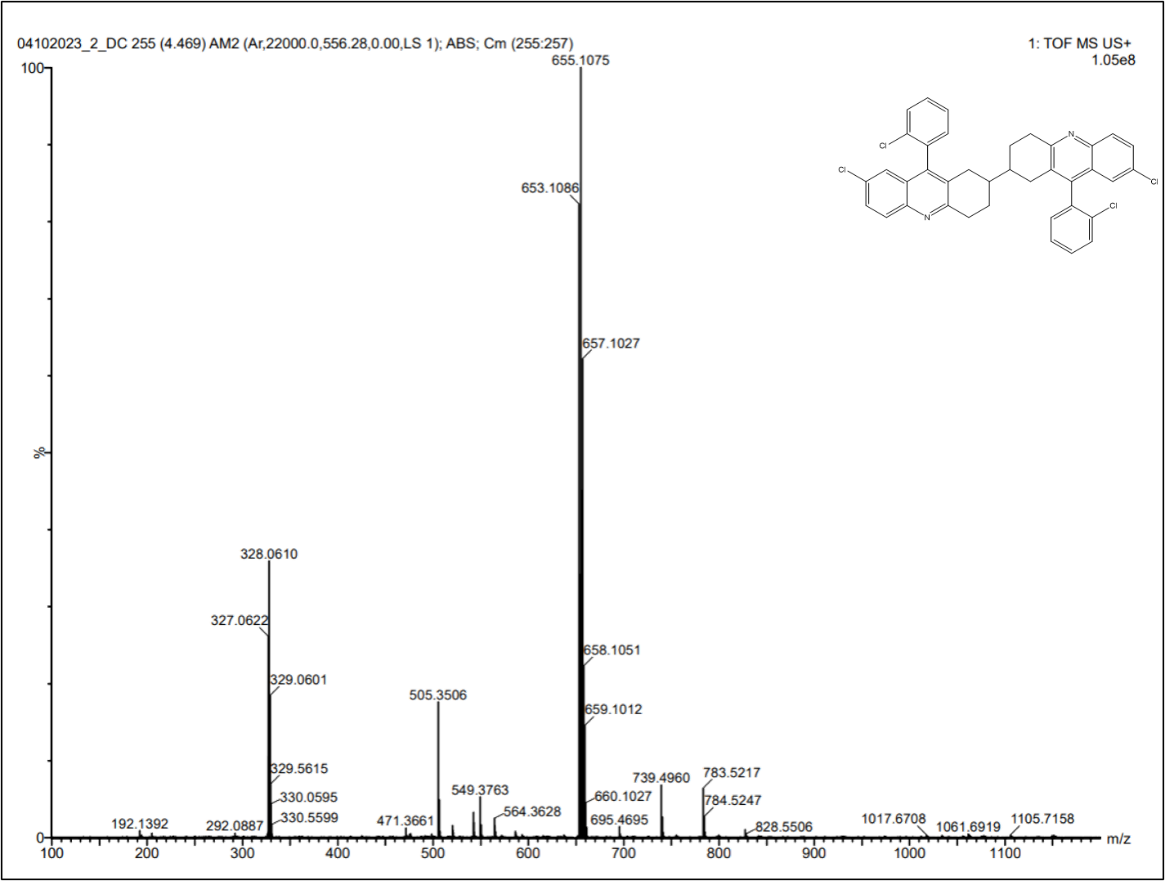


**Figure S41. HRMS spectrum of 7,7'-dichloro-9,9'-bis(2-chlorophenyl)-1,1',2,2',3,3',4,4'-octahydro-2,2'-biacridine (4c)**


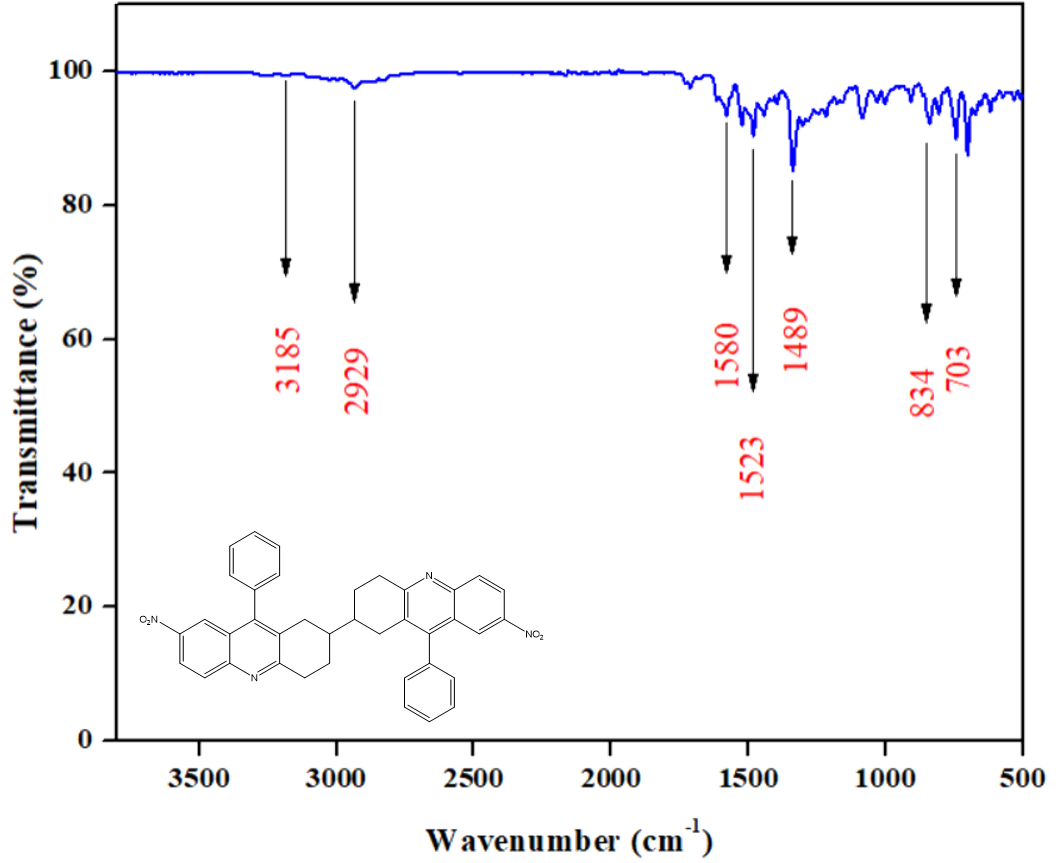


**Figure S42. FTIR spectrum of 7,7'-dinitro-9,9'-diphenyl-1,1',2,2',3,3',4,4'-octahydro-2,2'-biacridine (4d)**


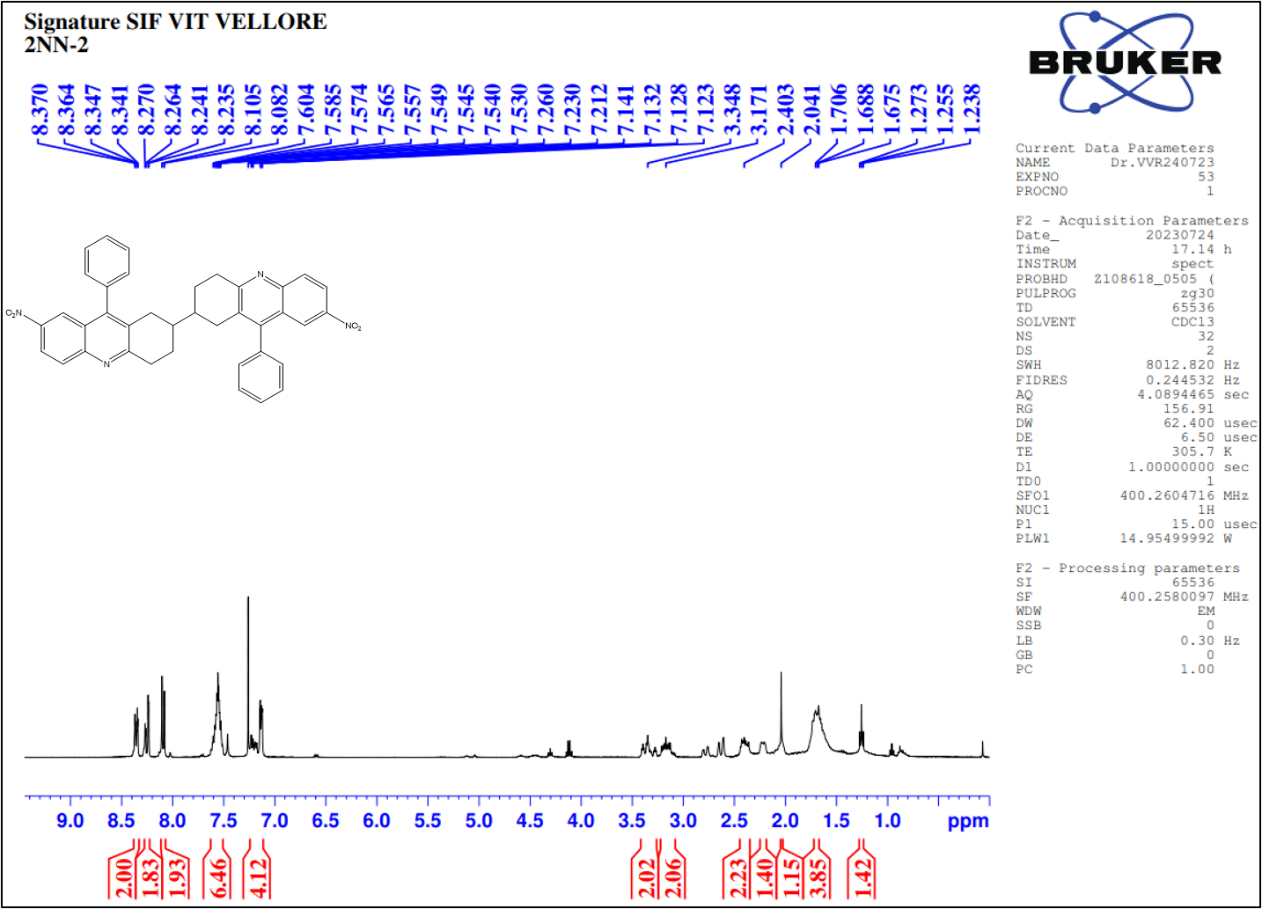


**Figure S43. ^1^H- NMR spectrum of 7,7'-dinitro-9,9'-diphenyl-1,1',2,2',3,3',4,4'-octahydro-2,2'-biacridine (4d)**

**
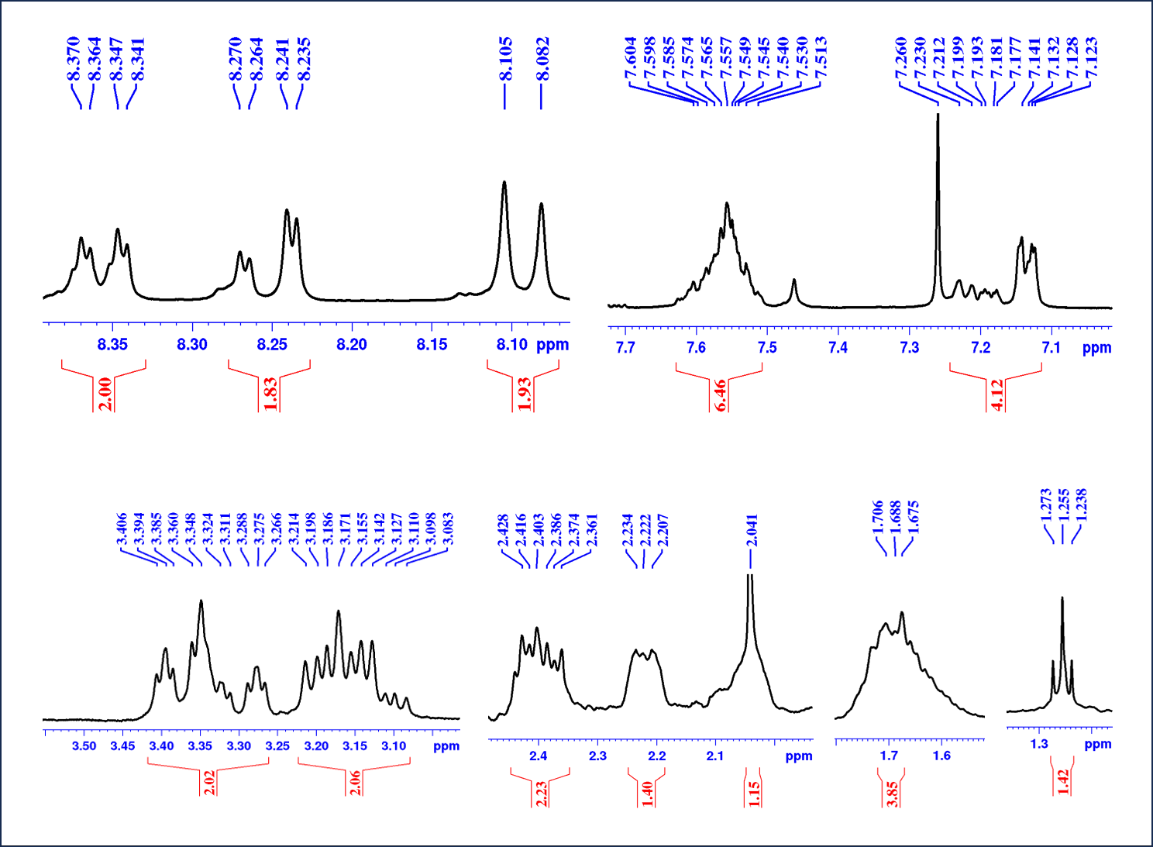
**

**Figure S44. Enlarged ^1^H- NMR spectrum of 7,7'-dinitro-9,9'-diphenyl-1,1',2,2',3,3',4,4'-octahydro-2,2'-biacridine (4d)**


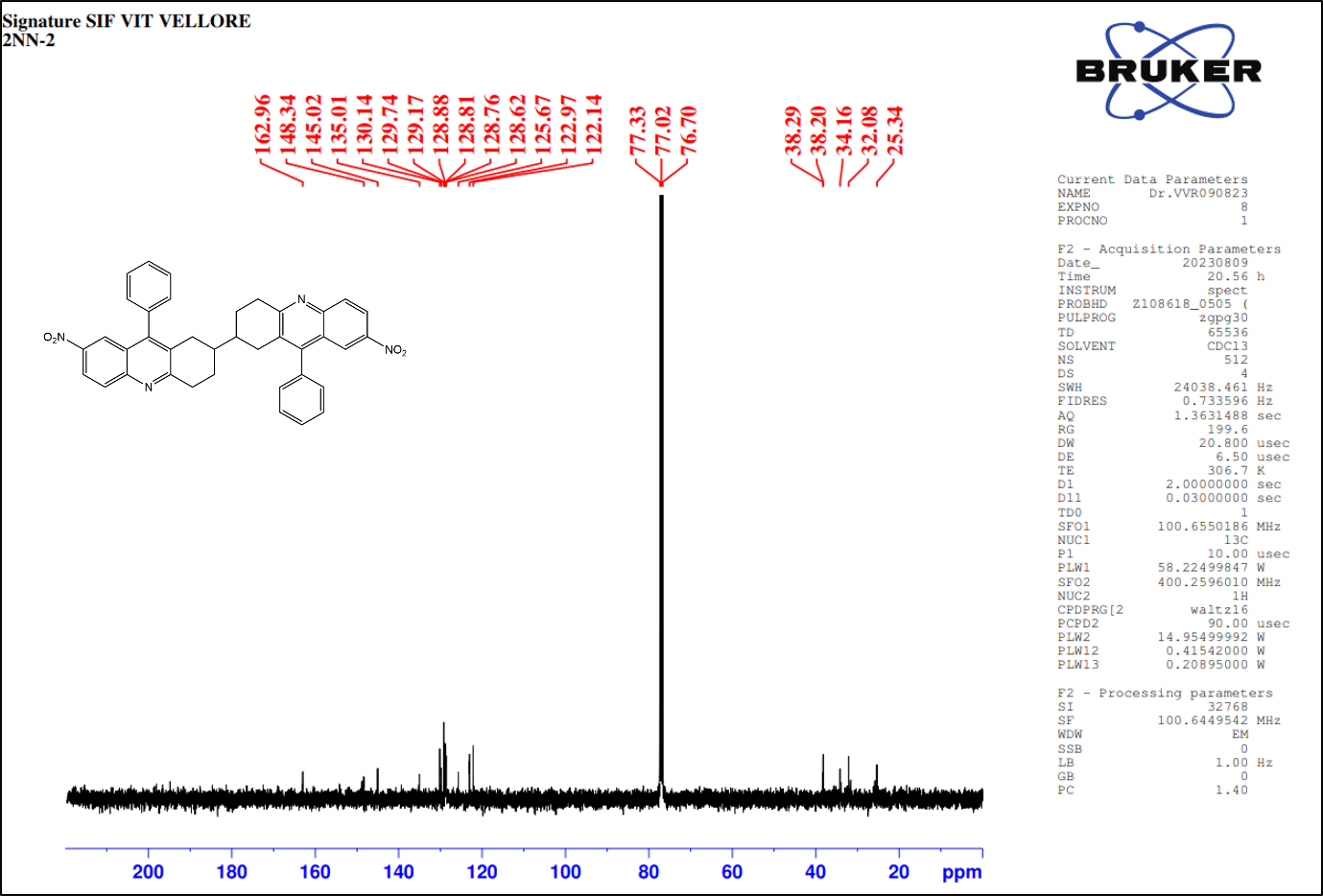


**Figure S45. ^13^C- NMR spectrum of7,7'-dinitro-9,9'-diphenyl-1,1',2,2',3,3',4,4'-octahydro-2,2'- biacridine (4d)**


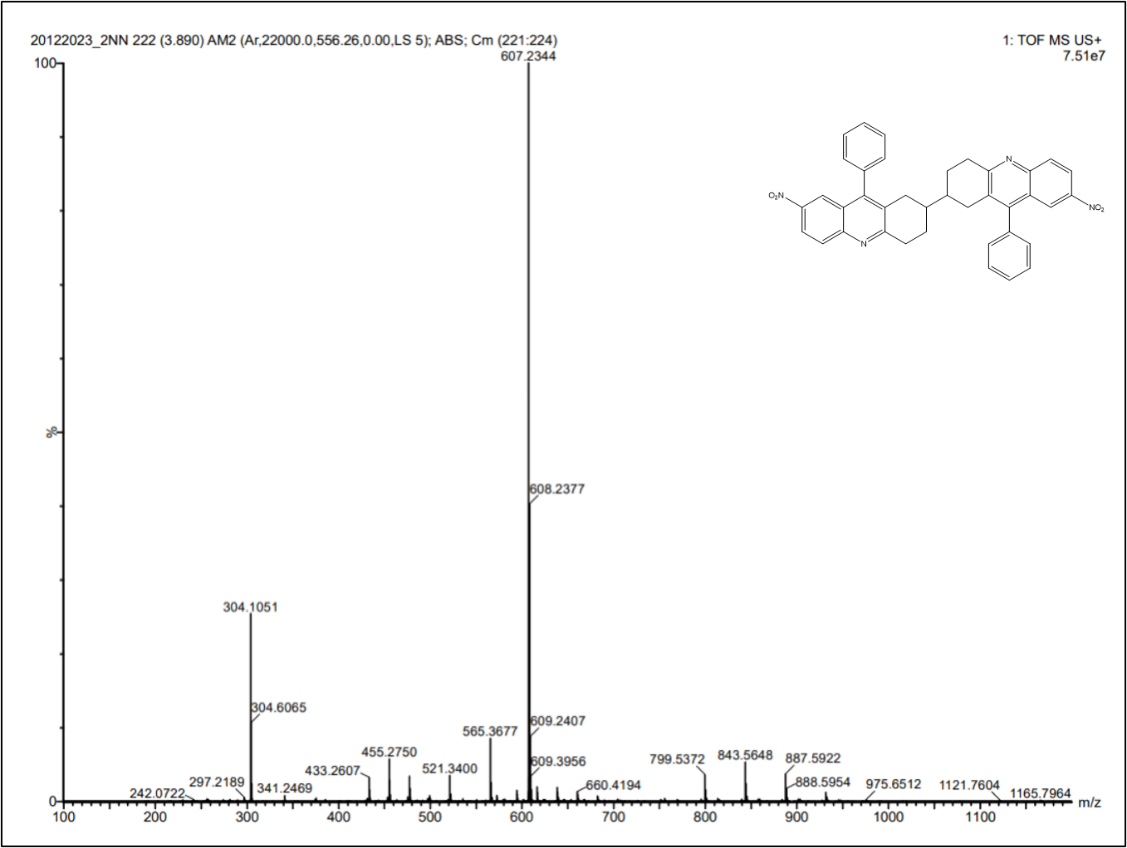


**Figure S46. HRMS spectrum of 7,7'-dinitro-9,9'-diphenyl-1,1',2,2',3,3',4,4'-octahydro-2,2'-biacridine (4d)**

**Table S1. Different synthesized deep eutectic solvents with their molecular ratio**

| S. No | DES | Structure | | Molar Ratio |
| --- | --- | --- | --- | --- |
| 1. | ChCl -Malonic acid |  |  | 1:1 |
| 2. | ChCl -oxalic acid |  |  | 1:1 |
| 3. | **ChCl-Ethylene glycol** | **** | **** | **1:2** |
| 4. | ChCl -Citric acid |  |  | 2:1 |
| 5. | ChCl -Tartaric acid |  |  | 1:1 |
| 6. | ChCl – Urea |  |  | 1:2 |
| 7. | ChCl - Fructose |  |  | 2:1 |
| 8. | ChCl - Caffeic acid |  |  | 2:1 |

**Table S2. Physical properties of the synthesized compounds 3a to 4d, calculated using DFT**

| **Compound** | **HOMO (eV)** | **LUMO (eV)** | **ΔE** | **μ (D)** | **Molecular weight** | **λ_max_**  **theoretical (nm)** |
| --- | --- | --- | --- | --- | --- | --- |
| **3a** | -5.9284 | -1.3673 | 4.5611 | 3.0039 | 355.1936 | 326 |
| **3b** | -6.1149 | -1.6024 | 4.5125 | 1.8283 | 389.1546 | 327 |
| **3c** | -6.6327 | -3.2381 | 5.5067 | 5.5067 | 400.1786 | 287 |
| **3d** | -6.6327 | -3.2544 | 3.0887 | 1.0387 | 423.1156 | 407 |
| **4a** | -5.8477 | -1.3113 | 4.5364 | 3.6147 | 516.2565 | 326 |
| **4b** | -6.0809 | -1.5921 | 4.4888 | 0.5541 | 584.178 | 328 |
| **4c** | -6.2591 | -1.5771 | 4.682 | 1.4245 | 652.1006 | 324 |
| **4d** | -6.7786 | -3.3440 | 3.4346 | 5.6158 | 606.2267 | 428 |

**Table.S3. Selected transitions obtained from TD-DFT calculation of compounds 3a-4d**

| **Compounds** | **Λ max (nm)** | **Oscillator strength (f)** | **Energy (eV)** | **Selected major contributions** |
| --- | --- | --- | --- | --- |
| 3a | 326  299  295 | 0.0044  0.0001  0.1748 | 3.80  4.14  4.19 | H-1 → L (90%)  H-2 → L+1(94%)  H →L (89%) |
| 3b | 327  299  298 | 0.0056  0.0023  0.1741 | 3.78  4.14  4.15 | H-1 → L (70%)  H-2 → L+1 (71%)  H→L (87%) |
| 3c | 323  298  287 | 0.0024  0.0000  0.1302 | 3.83  4.14  4.31 | H-2 → L(60%)  H-1 → L+2 (40%)  H→ L(92%) |
| 3d | 568  429  407 | 0.0001  0.0044  0.0973 | 2.18  2.88  3.04 | H-2→ L+1 (53%)  H →L+1 (41%)  H →L+1 (82%) |
| 4a | 326  297 | 0.0036  0.1071 | 3.79  4.16 | H-2→L (34%)  H→ L+1 (56%) |
| 4b | 328  300 | 0.0047  0.2009 | 3.77  4.12 | H-1→ L (31%)  H→ L+1 (57%) |
| 4c | 324  288 | 0.0004  0.1893 | 3.82  4.29 | H-2→ L (47%)  H→ L+1 (52%) |
| 4d | 576  428 | 0.0001  0.0087 | 2.14  2.89 | H-5→ L+1 (19%)  H-4→ L+1(20%) |

**Table S4: Docking energy results of compounds**

| **Compounds** | **Binding energy** | **Ligand efficiency** | **Inhibitory constant, Ki (um)** | **Inter molecular energy** | **vdW+H bond+ desolv energy** | **Electr ostatic energy** | **Torsional energy** | **Total internalunbound energy** |
| --- | --- | --- | --- | --- | --- | --- | --- | --- |
| **3a** | **-8.03** | **-0.3** | **1.3** | **-8.62** | **-8.57** | **-0.06** | **0.6** | **-0.96** |
| **3b** | **-9.98** | **-0.36** | **48.49** | **-10.58** | **-10.68** | **0.1** | **0.6** | **-0.68** |
| **3c** | **-8.08** | **-0.28** | **1.2** | **-8.67** | **-8.72** | **0.05** | **0.6** | **-1.38** |
| **3d** | **-8.8** | **-0.29** | **352.05** | **-9.7** | **-8.94** | **-0.76** | **0.89** | **-1.11** |
| **3e** | **-6.63** | **-0.24** | **13.76** | **-7.53** | **-7.44** | **-0.09** | **0.89** | **-1.13** |
| **4a** | **-10.28** | **-0.26** | **28.96** | **-11.18** | **-11.13** | **-0.05** | **0.89** | **-2.22** |
| **4b** | **-9.36** | **-0.22** | **136.71** | **-10.26** | **-10.18** | **-0.08** | **0.89** | **-1.8** |
| **4c** | **-9.78** | **-0.22** | **68.3** | **-10.67** | **-10.64** | **-0.03** | **0.89** | **-2.86** |
| **4d** | **-9.38** | **-0.2** | **132.09** | **-10.88** | **-10.67** | **-0.2** | **1.49** | **-2.78** |

**S.2. 2D and 3D docking images of the derivatives 3a to 4d**


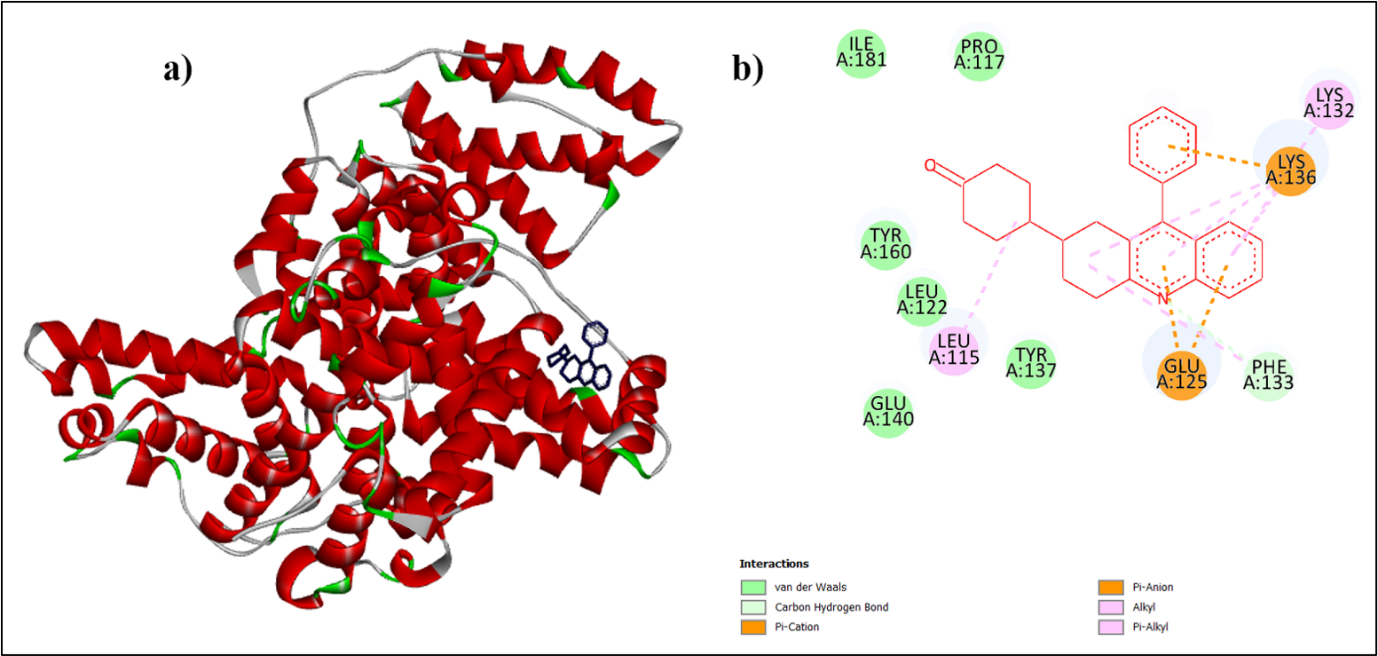


**Figure S47. a) 3D and 2D (b) interactions of reference 3a with receptor (PDB ID CODE: 4F5S)**


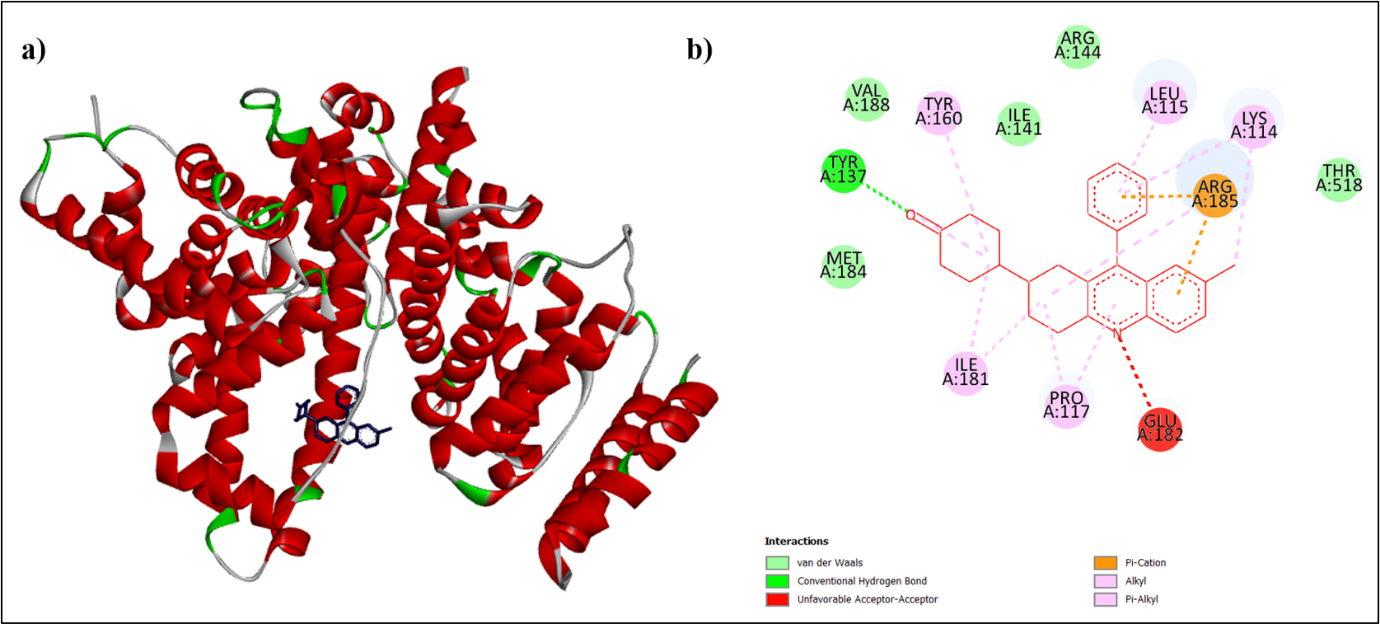


**Figure S48. a) 3D and b) 2D interactions of reference 3b with receptor (PDB ID CODE:4F5S)**


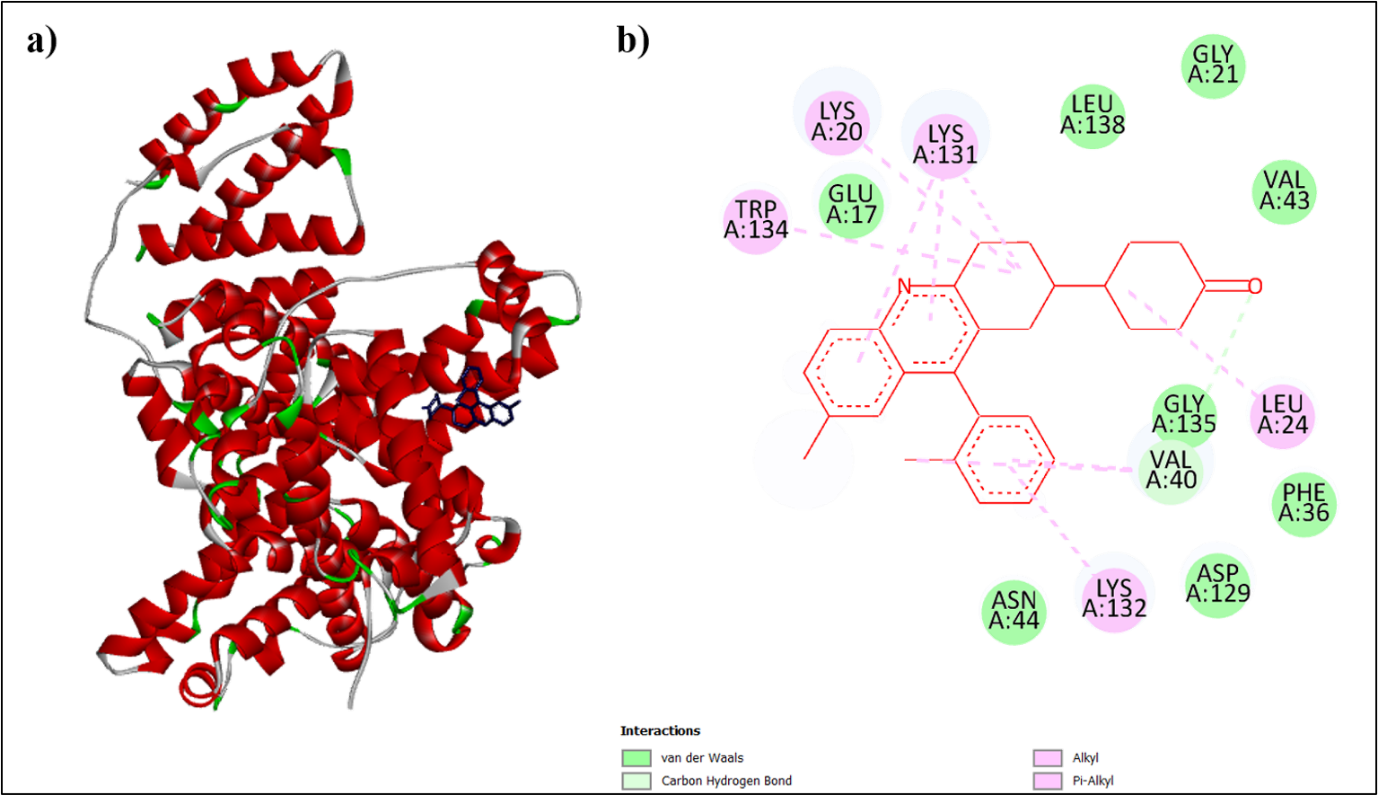


**Figure S49. a) 3D and b) 2D interactions of reference 3c with receptor (PDB ID CODE:4F5S)**


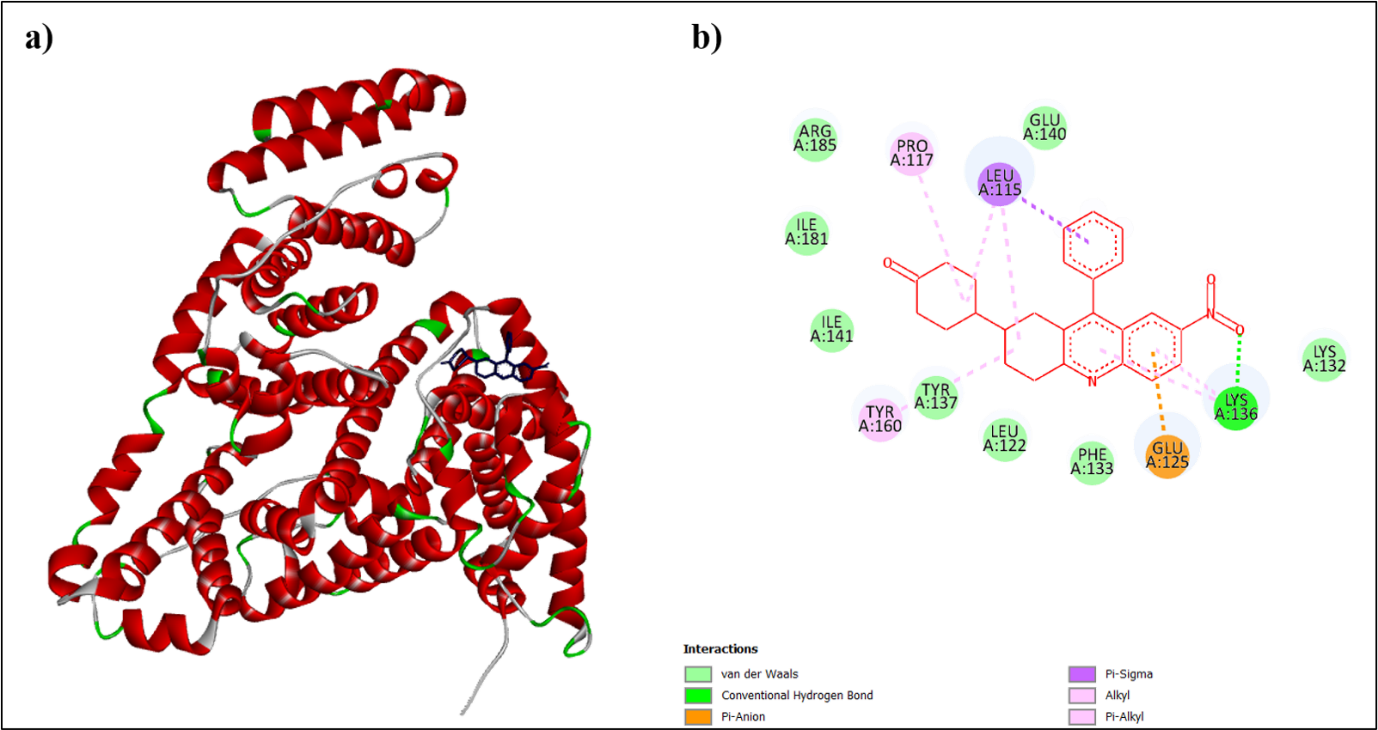


**Figure S50. a) 3D and b) 2D interactions of reference 3d with receptor (PDB ID CODE: 4F5S)**

**
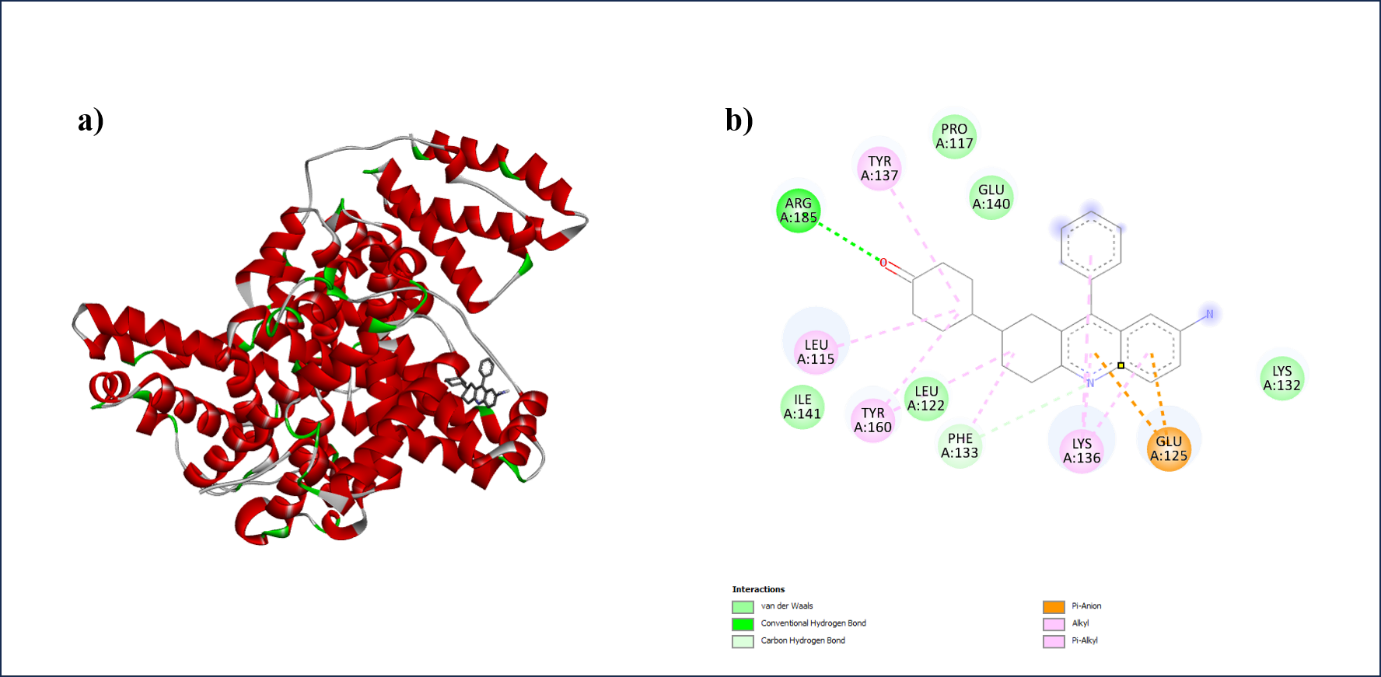
**

**Figure S51. a) 3D and b) 2D interactions of reference 3e with receptor (PDB ID CODE: 4F5S)**


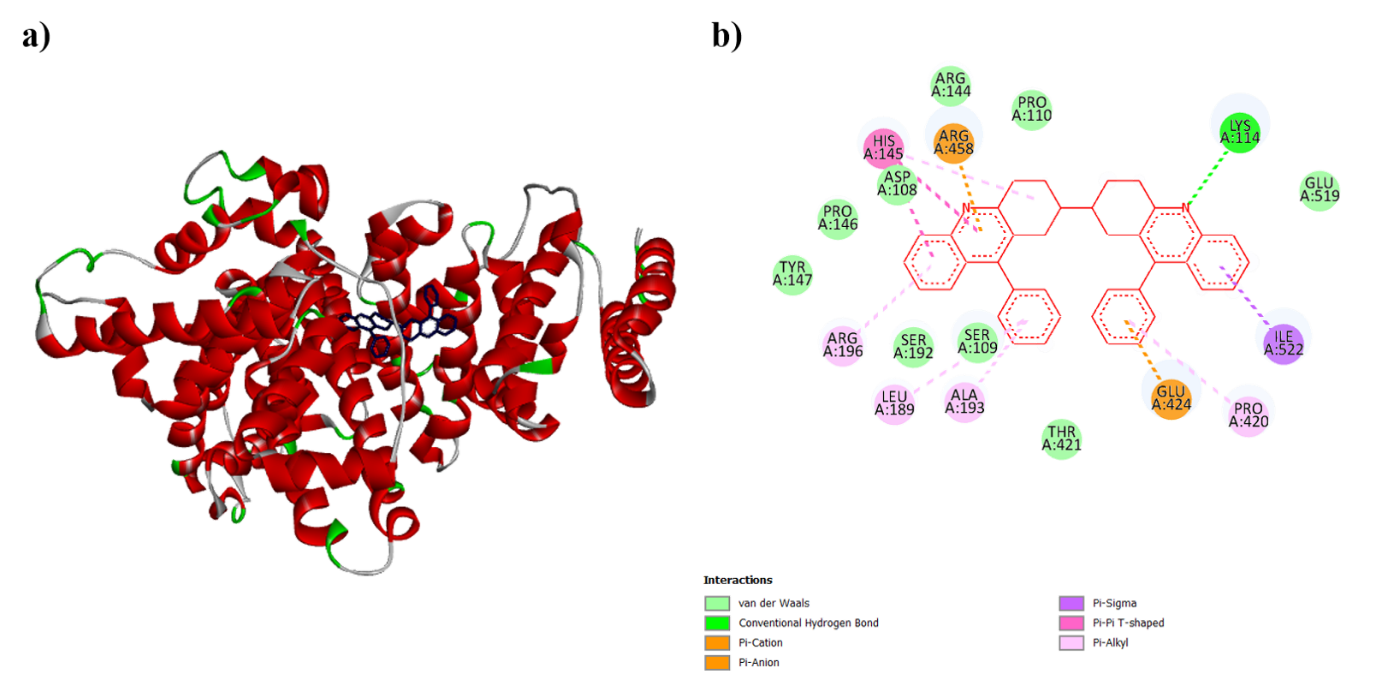


**Figure S52. a) 3D and b) 2D interactions of reference 4a with receptor (PDB ID CODE: 4F5S)**


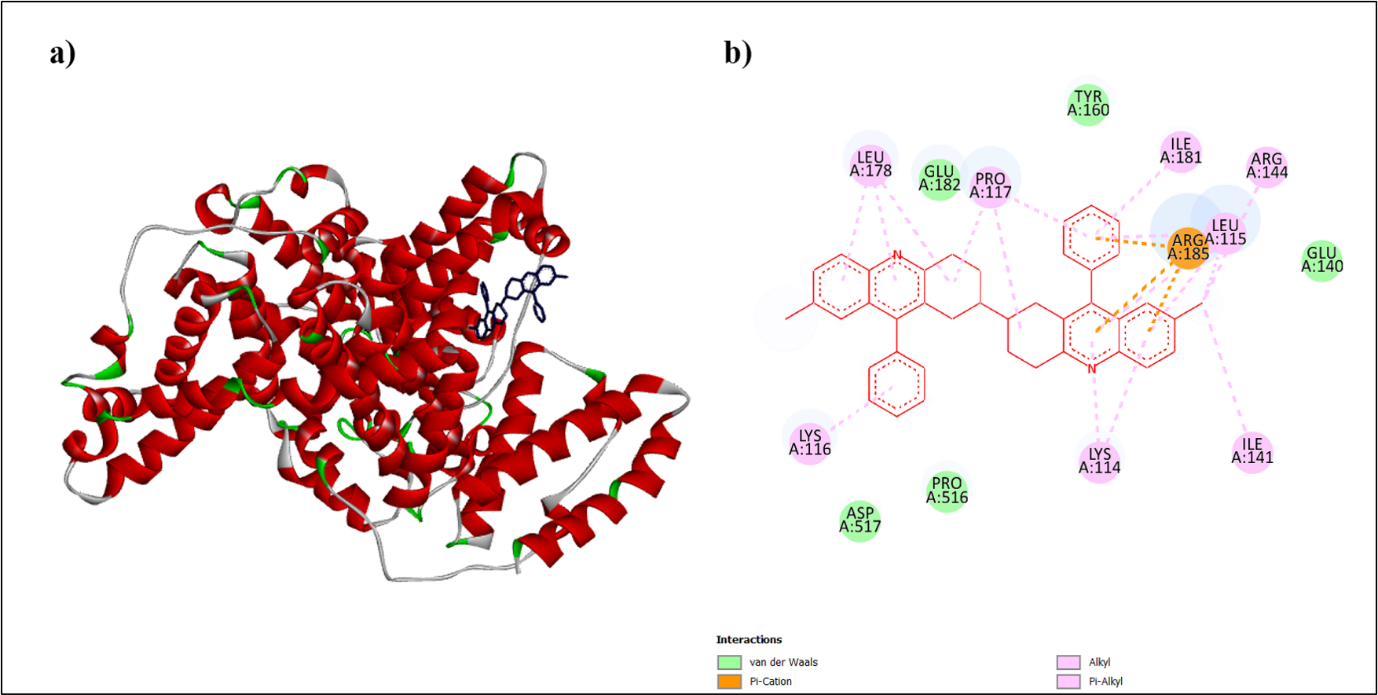


**Figure S53. a) 3D and b) 2D interactions of reference 4b with receptor (PDB ID CODE: 4F5S)**


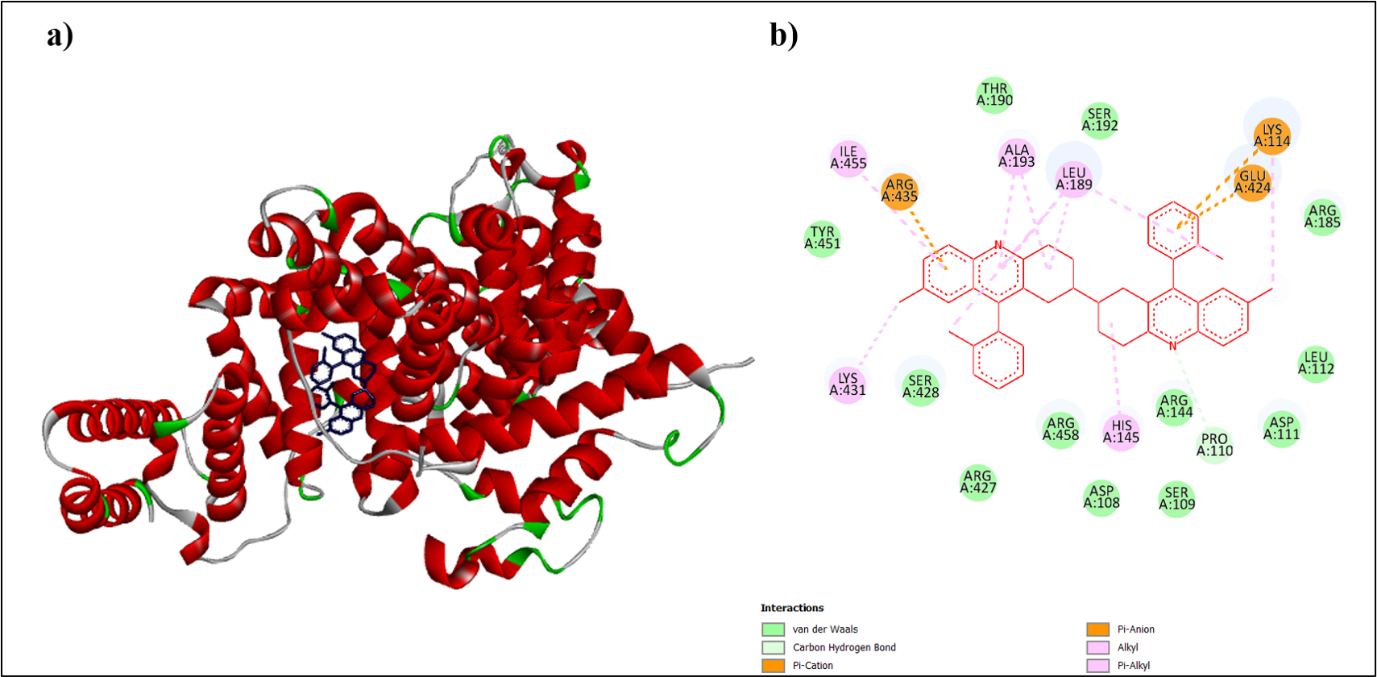


**Figure S54. a) 3D and b) 2D interactions of reference 4c with receptor (PDB ID CODE: 4F5S)**


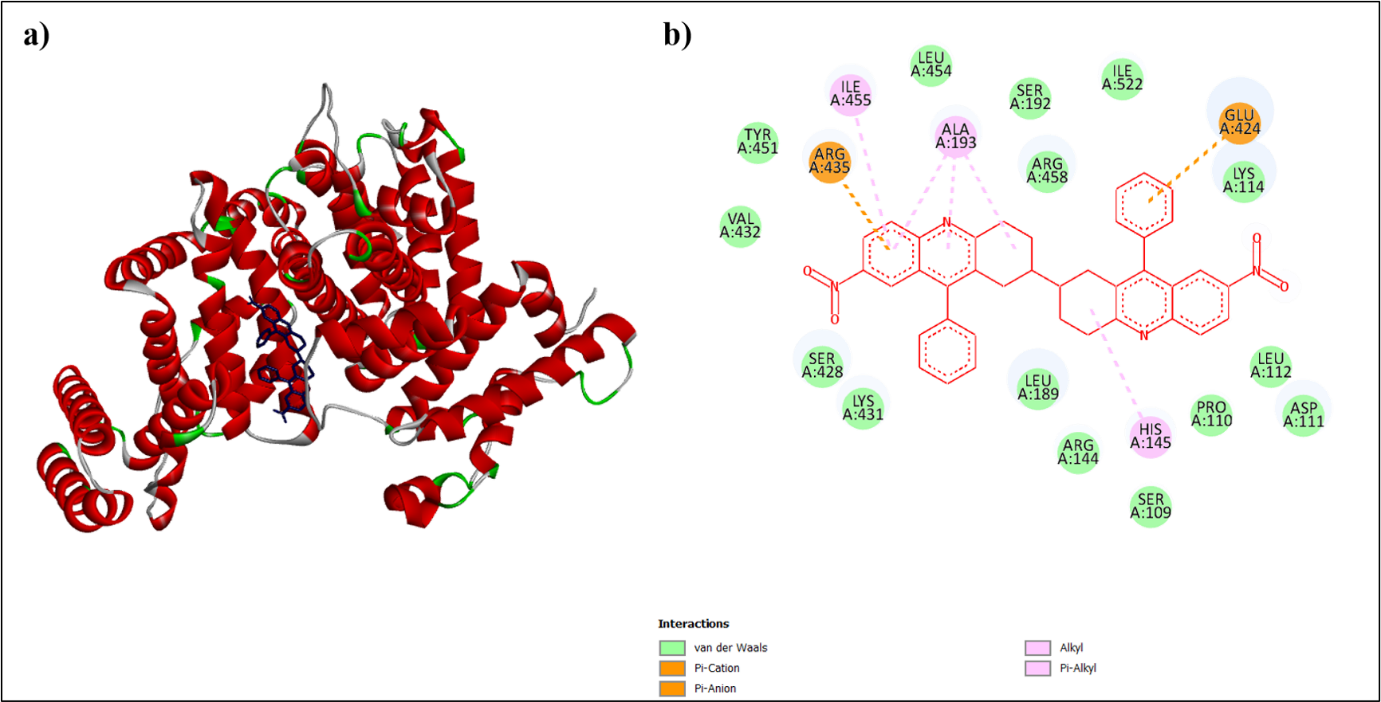


**Figure S55. a) 3D and b) 2D interactions of reference 4d with receptor (PDB ID CODE: 4F5S)**

**Table** **S5. Crystal data and structure refinement for 3e.**

| Identification code | shelx |  |
| --- | --- | --- |
|  |  |  |
| Empirical formula | C_25_ H_26_ N_2_ O |  |
|  |  |  |
| Formula weight | 370.48 |  |
|  |  |  |
| Temperature | 100 K |  |
|  |  |  |
| Wavelength | 0.71073 A |  |
|  |  |  |
| Crystal system, space group | Orthorhombic, P 21 21 21 |  |
| Unit cell dimensions | a = 10.1321(6) A alpha = 90 deg. b = 10.9511(6) A beta = 90 deg. c = 17.6995(11) A gamma = 90 deg. |  |
|  |  |  |
|  |  |  |
| Volume | 1963.9(2) A^3 |  |
|  |  |  |
| Z, Calculated density | 4, 1.253 Mg/m^3 |  |
|  |  |  |
| Absorption coefficient | 0.076 mm^-1 |  |
|  |  |  |
| F(000) | 792 |  |
|  |  |  |
| Crystal size | 0.599 x 0.170 x 0.143 mm |  |
|  |  |  |
| Theta range for data collection | 2.959 to 25.359 deg. |  |
|  |  |  |
| Limiting indices | -12<=h<=12, -13<=k<=13, -21<=l<=21 |  |
|  |  |  |
| Reflections collected / unique | 34482 / 3585 [R(int) = 0.0680] |  |
| Completeness to theta = 25.242 | 99.80% |  |
|  |  |  |
| Absorption correction | Semi-empirical from equivalents |  |
|  |  |  |
| Max. and min. transmission | 1.0000 and 0.5800 |  |
|  |  |  |
| Refinement method | Full-matrix least-squares on F^2 |  |
|  |  |  |
| Data / restraints / parameters | 3585 / 8 / 280 |  |
|  |  |  |
| Goodness-of-fit on F^2 | 1.046 |  |
|  |  |  |
| Final R indices [I>2sigma(I)] | R1 = 0.0623, wR2 = 0.1585 |  |
|  |  |  |
| R indices (all data) | R1 = 0.0771, wR2 = 0.1710 |  |
|  |  |  |
| Absolute structure parameter | -1.6(10) |  |
|  |  |  |
| Extinction coefficient | 0.012(3) |  |
|  |  |  |
| Largest diff. peak and hole  CCDC Number | 0.337 and -0.284 e.A^-3  2404164 |  |
|  |  |  |
|  |  |  |
|  |  |  |
|  |  |  |

**Table S6. Selected a) Bond Lengths (Å), b) Bond Angles (°), and c) Torsional angles (°) for 3e from the crystal structure**

**a)**

| **Atoms** | **Bond length (A^o^)** |
| --- | --- |
| C(1)-O(1) | 1.19 |
| C(1)-C(6) | 1.51 |
| C(1)-C(6') | 1.51 |
| C(4)-H(4) | 1.0 |
| C(7)-H(7) | 1.0 |
| C(10)-N(1) | 1.32 |

**b)**

| **Atoms** | **Bond angle (°)** |
| --- | --- |
| O(1)-C(1)-C(6) | 121.7 |
| O(1)-C(1)-C(6’) | 120.7 |
| C(3)-C(2)-H(2A) | 109.1 |
| C(3)-C(2)-H(2B) | 109.1 |
| C(14)-N(2)-H(2C) | 114 |
| C(14)-N(2)-H(2D) | 115 |

**c)**

| **Atoms** | **Torsional angle (°)** |
| --- | --- |
| C(5)-C(4)-C(7)-C(8) | 178.8 |
| C(3)-C(4)-C(7)-C(19) | -178.0 |
| C(18)-C(17)-C(20)-C(25) | 95.9 |
| C(16)-C(17)-C(20)-C(21) | 93.4 |

**S.2. Solvatochromism study of compounds 3a to 4d in various solvents**

The absorption maxima of derivatives 3a to 3d and their dimers 4a to 4d have been recorded with various solvents to study the effects of the non-polar to polar solvents on it using UV-Vis spectrophotometer which is tabulated below in Table.S2. The analysis shows that 4c and 4d show a very slight red shift and the remaining derivatives exhibit no such shifts (Fig.S56 and Fig. S57)**.** These when compared with the 3e derivative, show that it is more active as a sensor due to ICT which enhances its activity towards an analyte.

**Table S7. Absorption maxima of 3a to 4d in various solvents**

| Solvent | Absorption, λ_abs_ (nm) of 3a | Absorption, λ_abs_ (nm) of 3b | Absorption, λ_abs_ (nm) of 3c | Absorption, λ_abs_ (nm) of 3d | Absorption, λ_abs_ (nm) of 4a | Absorption, λ_abs_ (nm) of 4b | Absorption, λ_abs_ (nm) of  4c | Absorption, λ_abs_ (nm) of 4b |
| --- | --- | --- | --- | --- | --- | --- | --- | --- |
| Toluene | 318 | 330 | 323 | 311 | 324 | 323 | 330 | 310 |
| DCM | 322 | 329 | 324 | 313 | 323 | 323 | 329 | 316 |
| THF | 317 | 322 | 322 | 309 | 323 | 322 | 323 | 309 |
| ACN | 320 | 328 | 323 | 312 | 322 | 321 | 328 | 310 |
| DMF | 318 | 322 | 323 | 313 | 323 | 322 | 323 | 310 |
| DMSO | 318 | 322 | 324 | 315 | 323 | 323 | 324 | 310 |

**
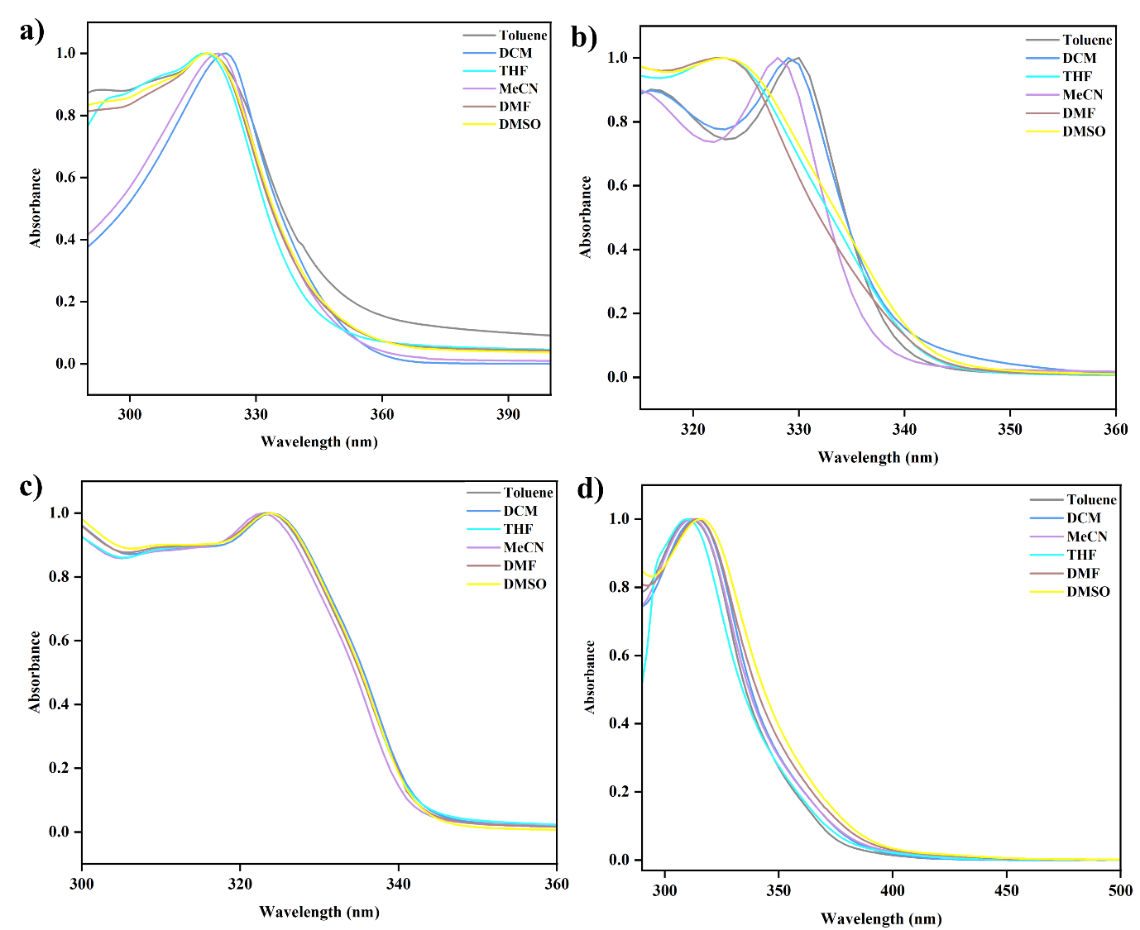
**

**Figure S56. Normalized absorption of 3a-3d in various solvents**

**
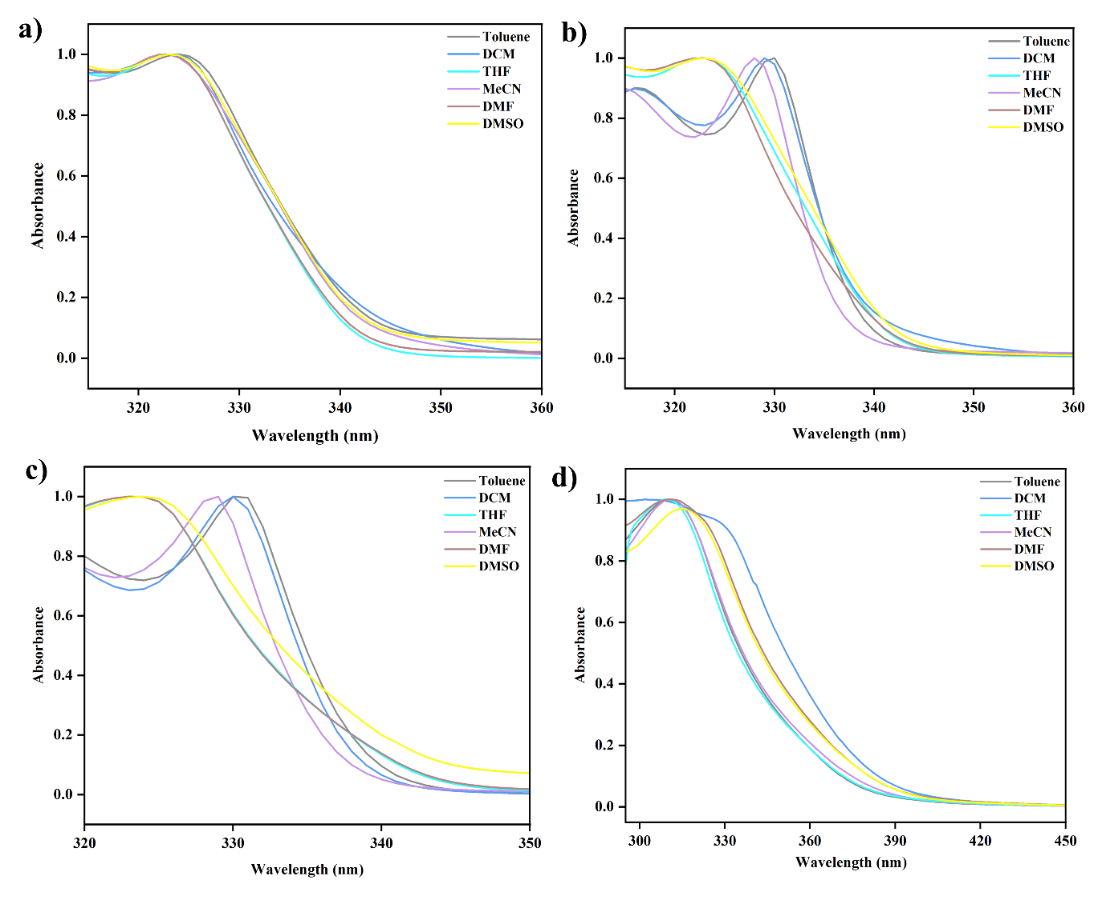
**

**Figure S57. Normalized absorption of 4a-4d in various solvent**

**S.3. General Procedure for the preparation of stock solution**

A stock solution of the sensor **3e** and various interfering compounds, namely, picric acid (PA), 2-nitrophenol (2NP), 3-nitrophenol (3NP), 4-nitrophenol (4NP), 3-niro aniline (3NA), 2-amino-4-nitrophenol (ANP), were prepared in CH_3_CN at concentrations of 1×10^−3^ M. The sensor was then subjected to both UV-Vis and Florescence spectral studies by taking 20 μl aliquots of analytes in 2 ml CH_3_CN solution containing 1x10^-5^ M of these probes in a quartz cuvette (1 cm x 1 cm).

**Table S8. Comparison of present work with earlier reports**

| S.No | Sensor | Analyte | Method of detection | LOD (M) | Reference |
| --- | --- | --- | --- | --- | --- |
| 1. | Pyridine-pyrazole based | PA | Turn on-off | 1.22 x 10^-4^ | [[1]](#one) |
| 2. | Ca(II)-MOF | PA | Turn-off | 1.90 x 10^-5^ | [[2]](#two) |
| 3. | ZnSe quantom dots | PA | Turn-off | 1.24 x 10^-5^ | [[3]](#three) |
| 4. | Debsyl based | PA | Turn-on | 7.2 x 10^-6^ | [[4]](#fouralone) |
| 5. | Histidine based | PA | Turn-off | 2.71 x 10-^6^ | [[5]](#fivealone) |
| 6. | Zn (II) complex based | PA | Turn-on-off | 9.09 x 10^-6^ | [[6]](#six) |
| 7. | Pyrene based sensor | PA | Turn-off | 8.14x 10^-7^ | [[7]](#seven) |
| 8. | Pyranone based | PA | Turn-on | 6.92 x 10^-7^ | [[8]](#eight) |
| 9. | Bispyrene based sensor | PA | Turn-off | 1.39 x 10^-7^ | [[9]](#nine) |
| **10.** | **Quinoline based sensor** | **PA** | **Quenching, turn off** | **1.77 x 10^-9^** | **Present work** |

**Table.S9. Selected transitions obtained from TD-DFT calculation of 3e**

| **Compounds** | **HOMO (eV)** | **LUMO (eV)** | **Energy**  **ΔE (eV)** | **Λ max (nm)** | **Oscillator strength (f)** | **Selected major contributions** |
| --- | --- | --- | --- | --- | --- | --- |
| A2N | -5.25  -6.69  -5.25 | -1.26  -0.73  -0.73 | 3.99  5.96  4.52 | 352  301  291 | 0.1516  0.0000  0.0002 | H → L (96%)  H-3 → L+1(68%)  H →L+1 (99.5%) |
| A2N+PA | -5.14  -5.14  -.7.08 | -4.67  -4.13  -4.67 | 1.35  1.58  2.41 | 914  790  703 | 0.0071  0.0201  0.0014 | H → L (90%)  H → L+1 (91%)  H-5 → L(38%) |

**Table.S10.FMO of 3e with energy in eV**

| Orbital | Orbital Number | Energy(ΔE) | Orbital Image |
| --- | --- | --- | --- |
| 101 | L+1 | -0.73 | 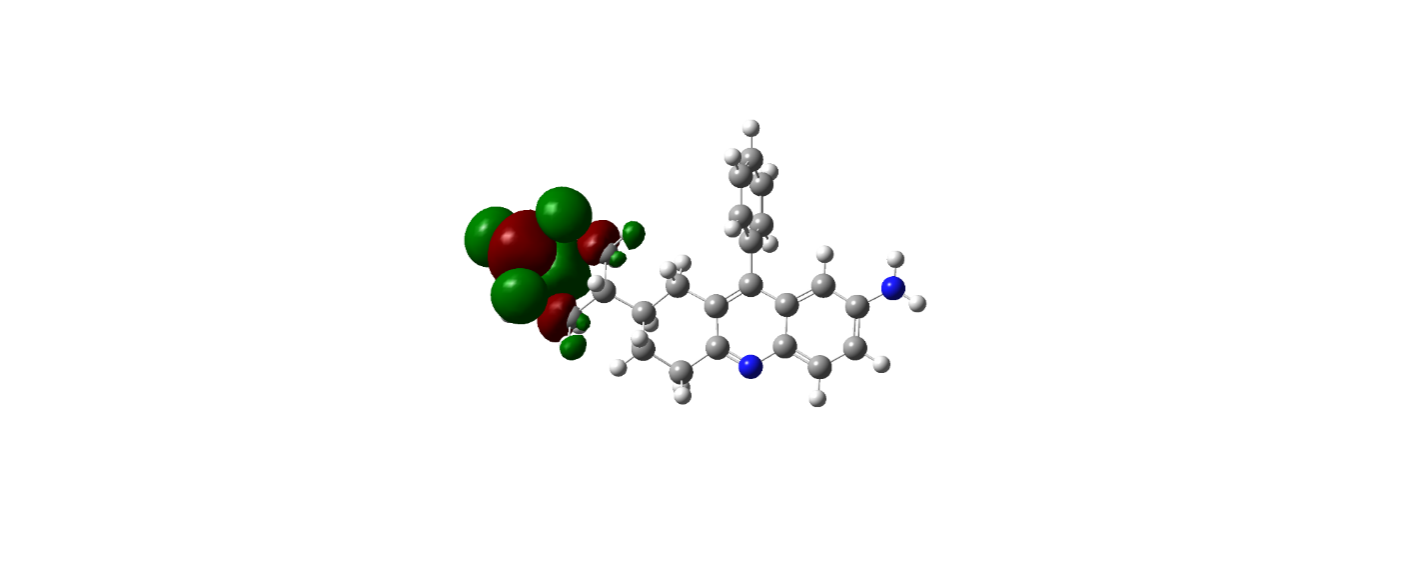 |
| 100 | LUMO | -1.26 | 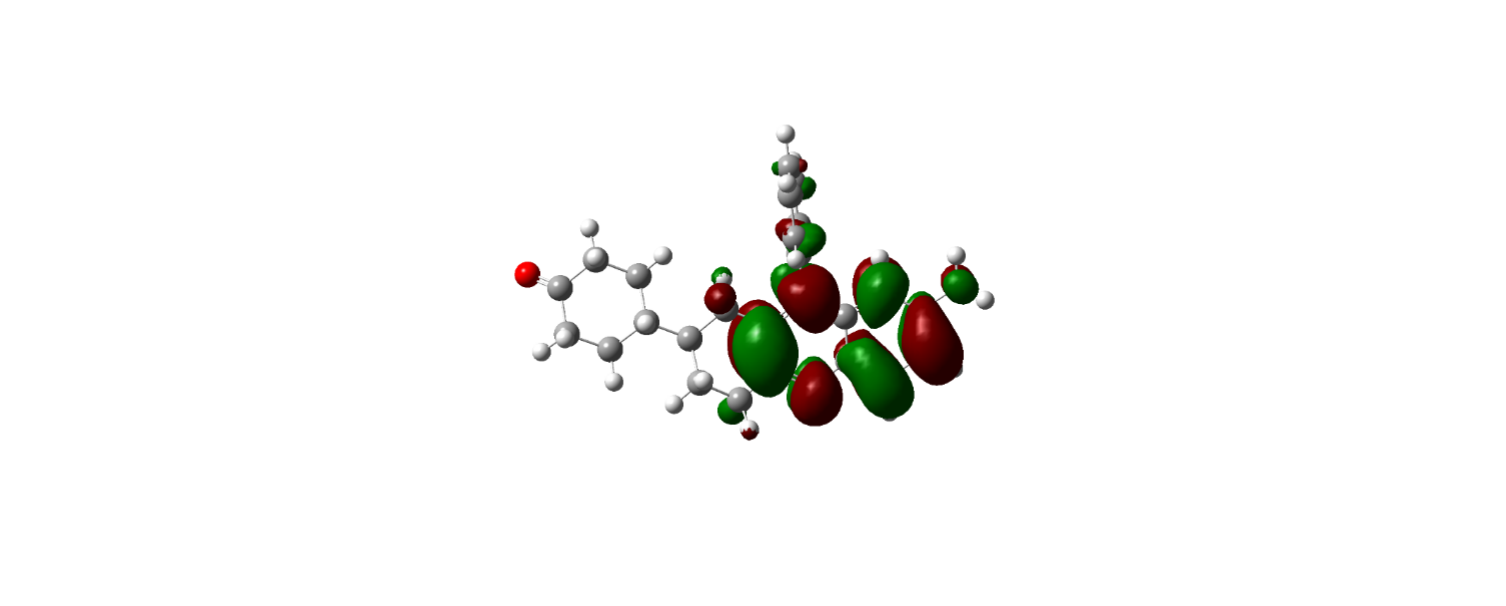 |
| 99 | HOMO | -5.25 | 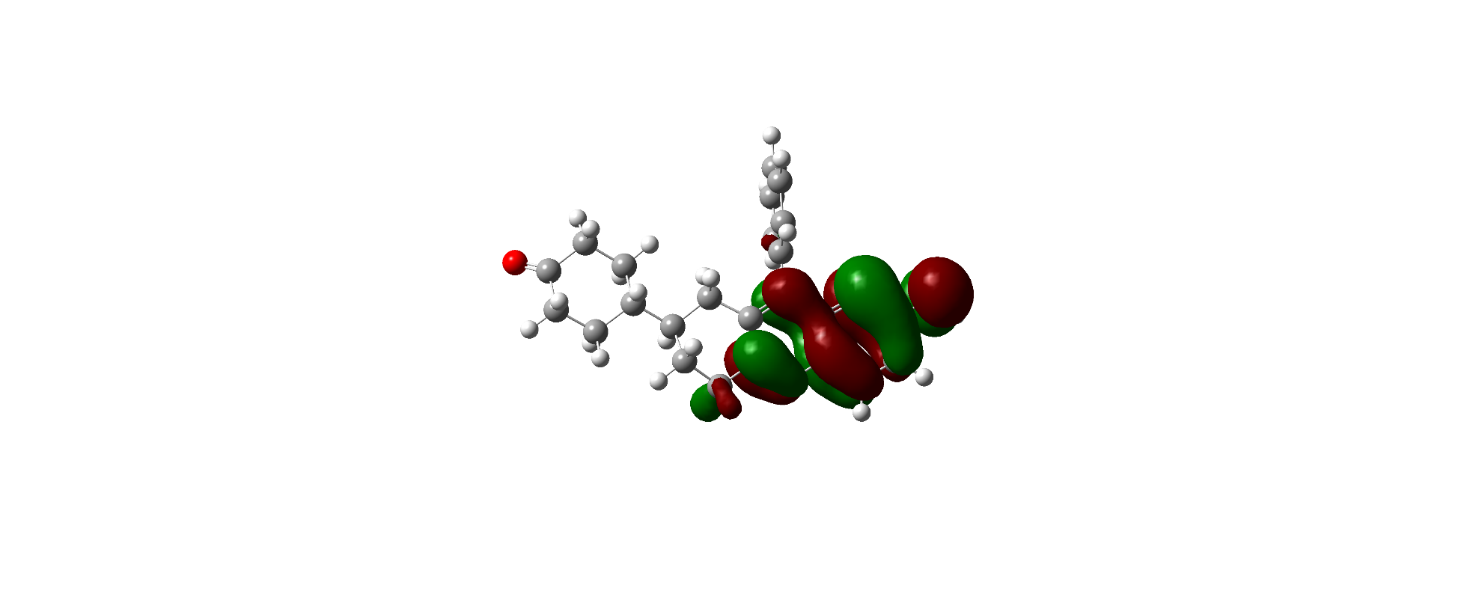 |
| 98 | HOMO-1 | -6.32 | 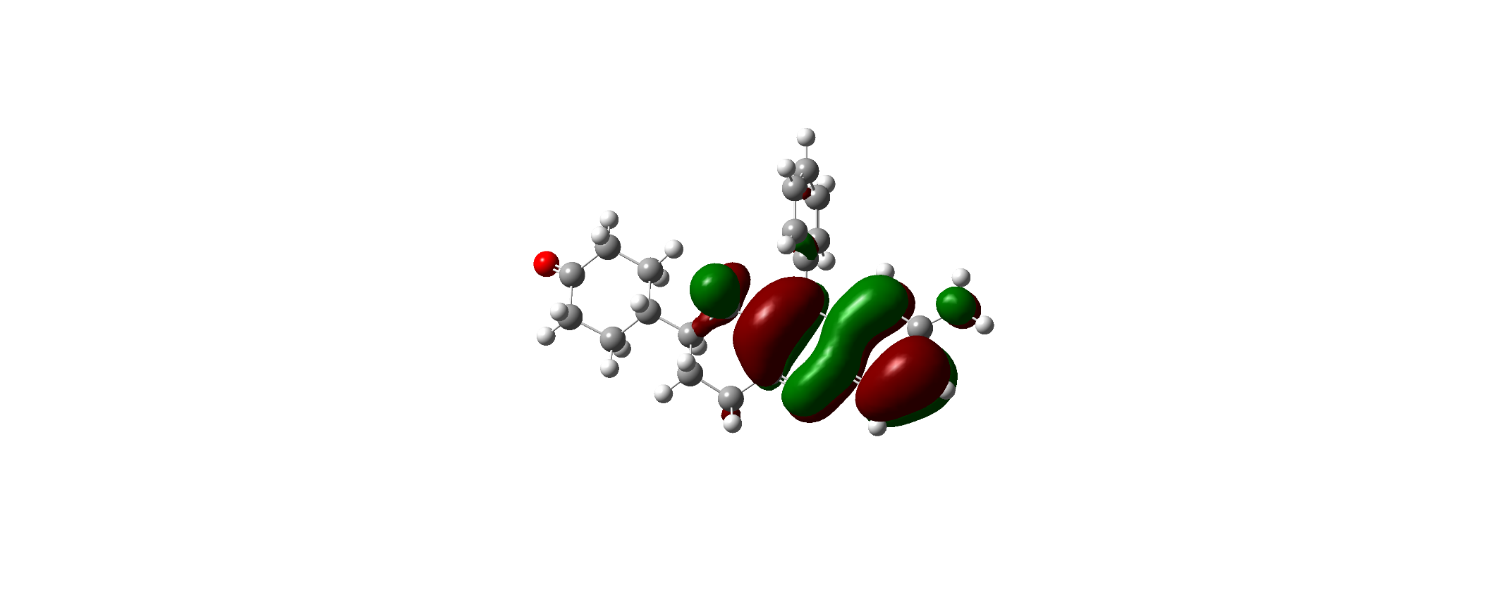 |
| 97 | HOMO-2 | -6.61 | 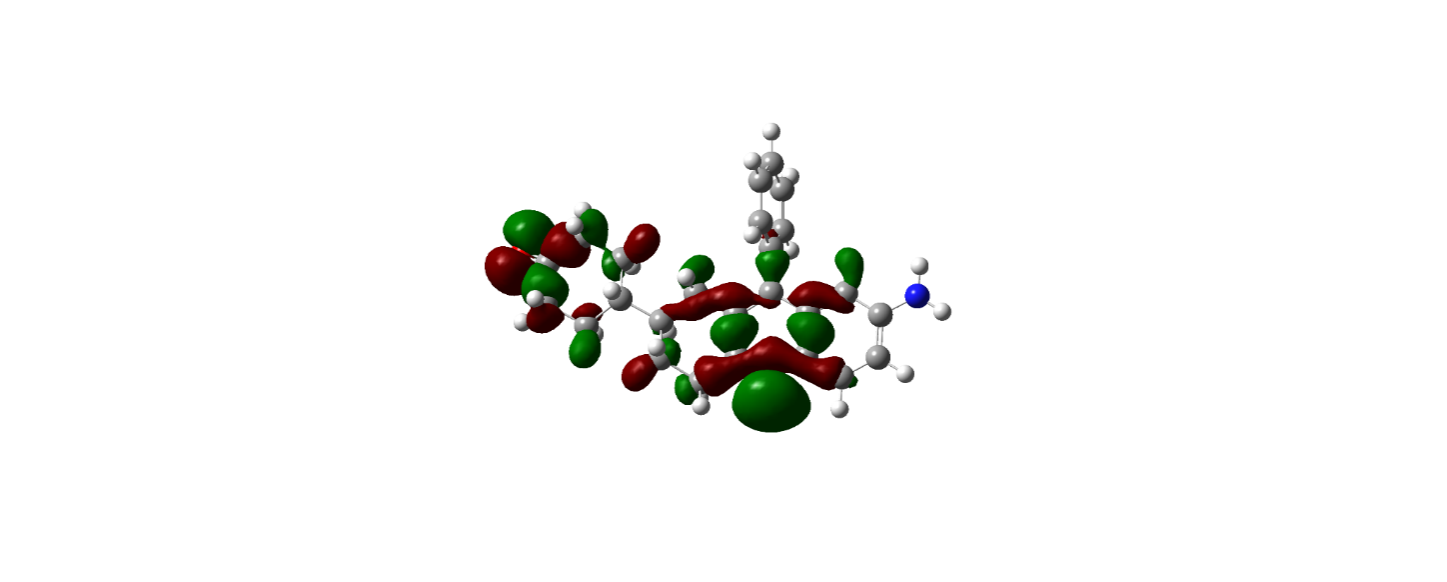 |
| 96 | HOMO-3 | -6.69 | 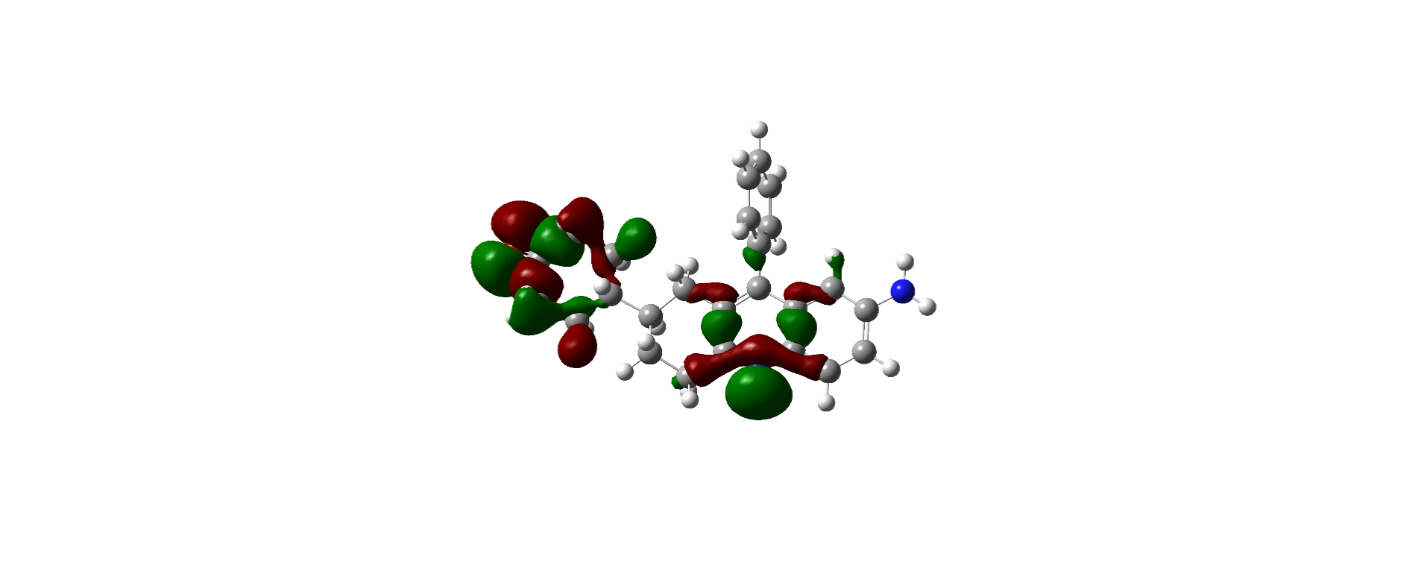 |

**Table.S11. FMO of 3e+PA with energy in eV**

| Orbital | Orbital Number | Energy(ΔE) | Orbital Image |
| --- | --- | --- | --- |
| 160 | LUMO+2 | -3.87 | 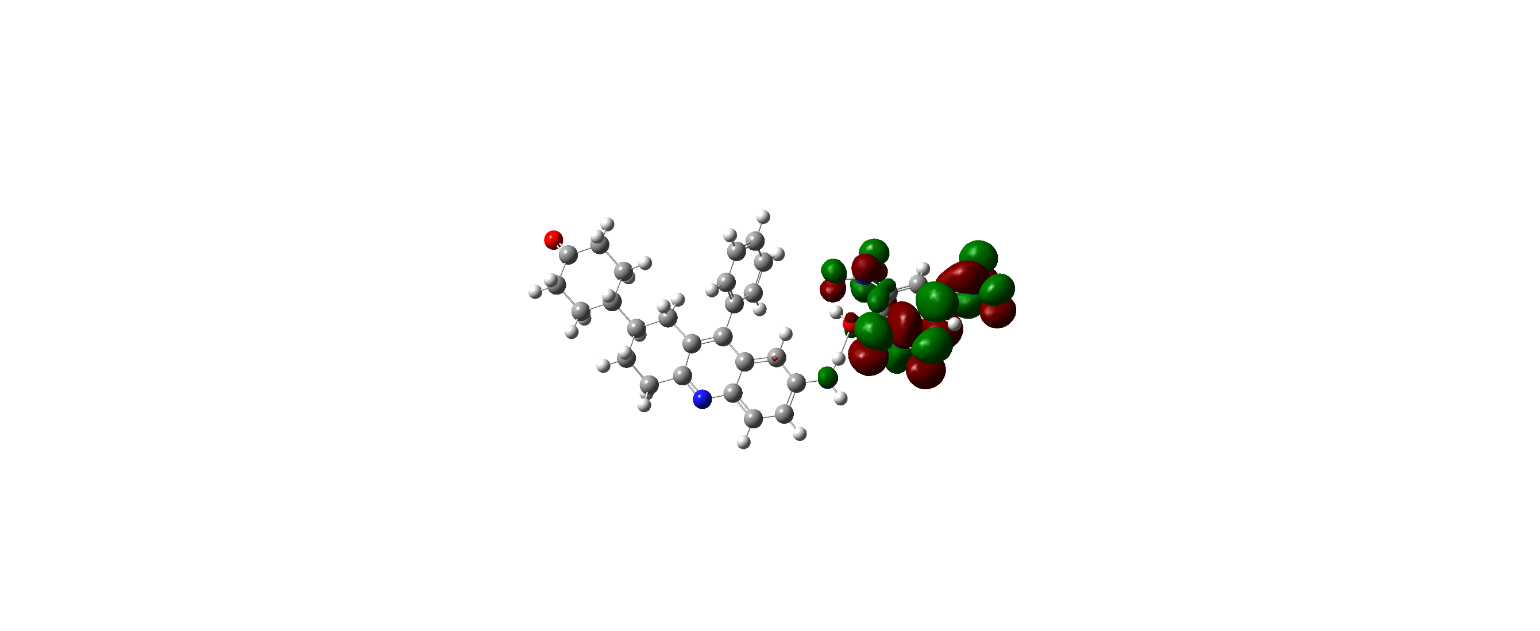 |
| 159 | LUMO+1 | -4.13 | 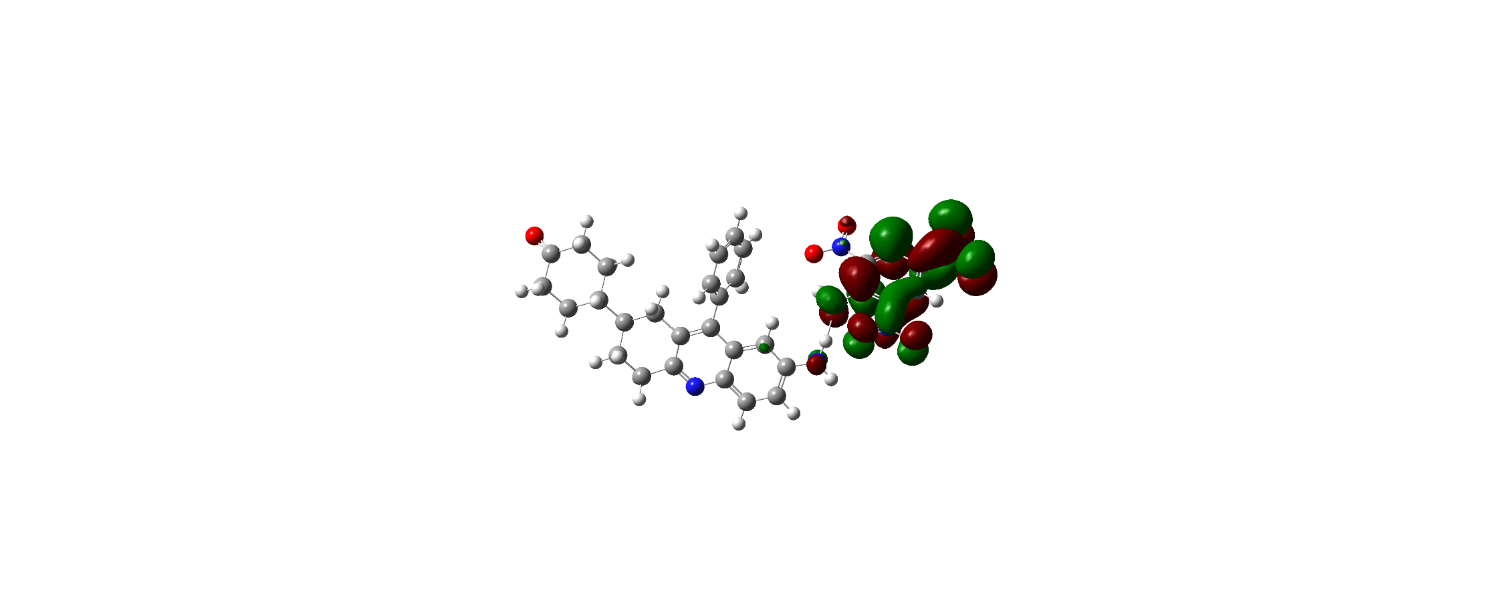 |
| 158 | LUMO | -4.67 | 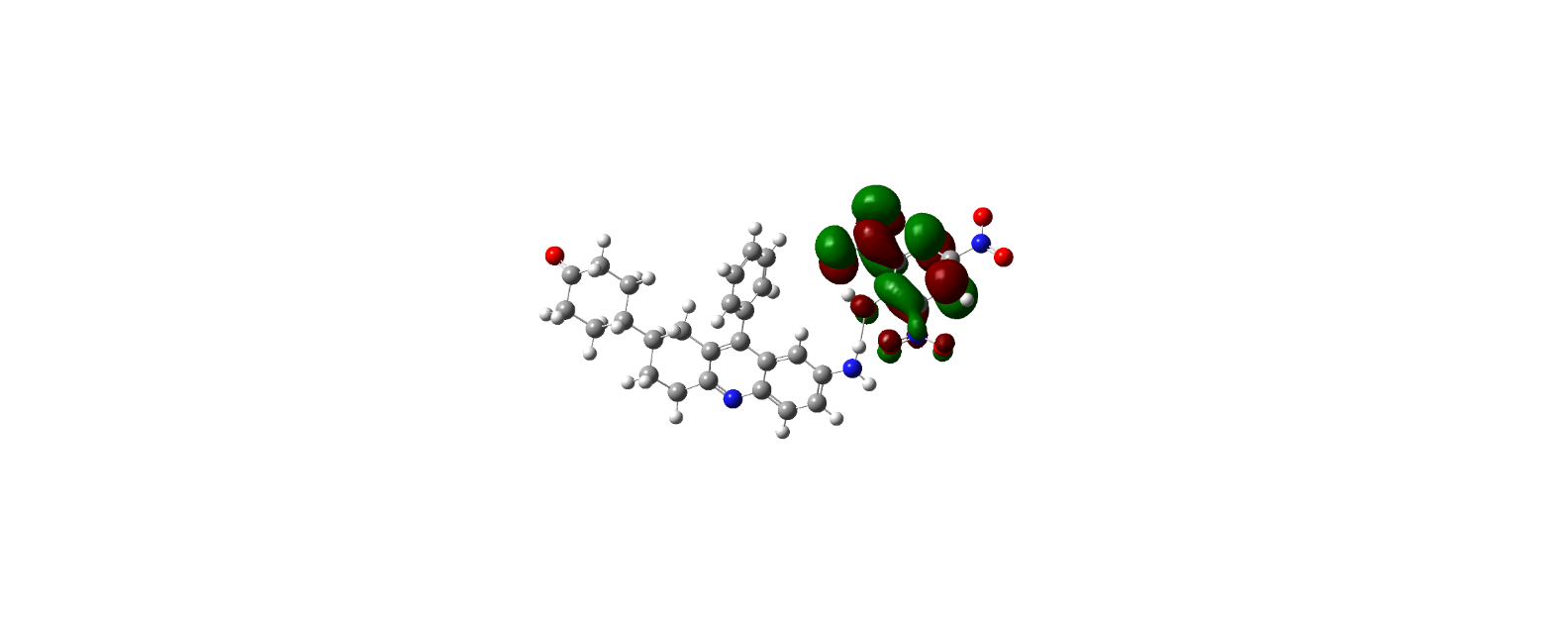 |
| 157 | HOMO | -5.14 | 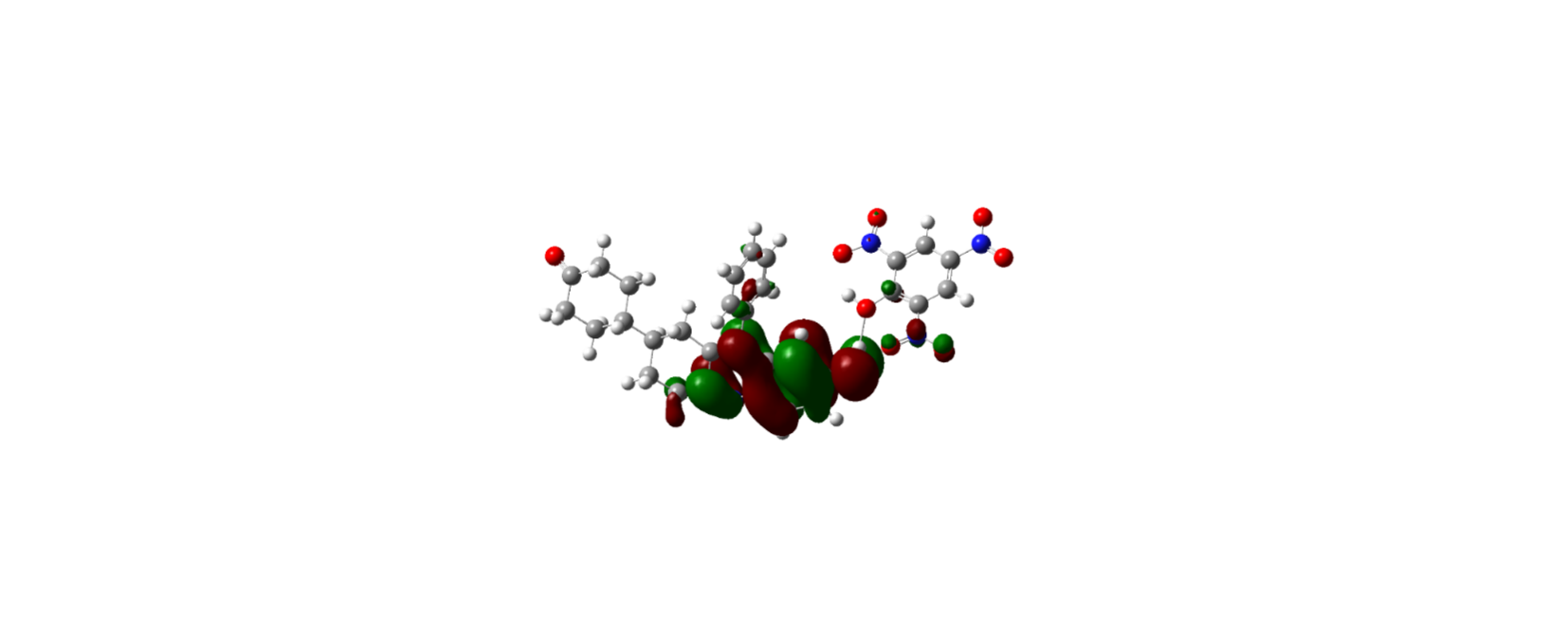 |
| 156 | HOMO-1 | -6.20 | 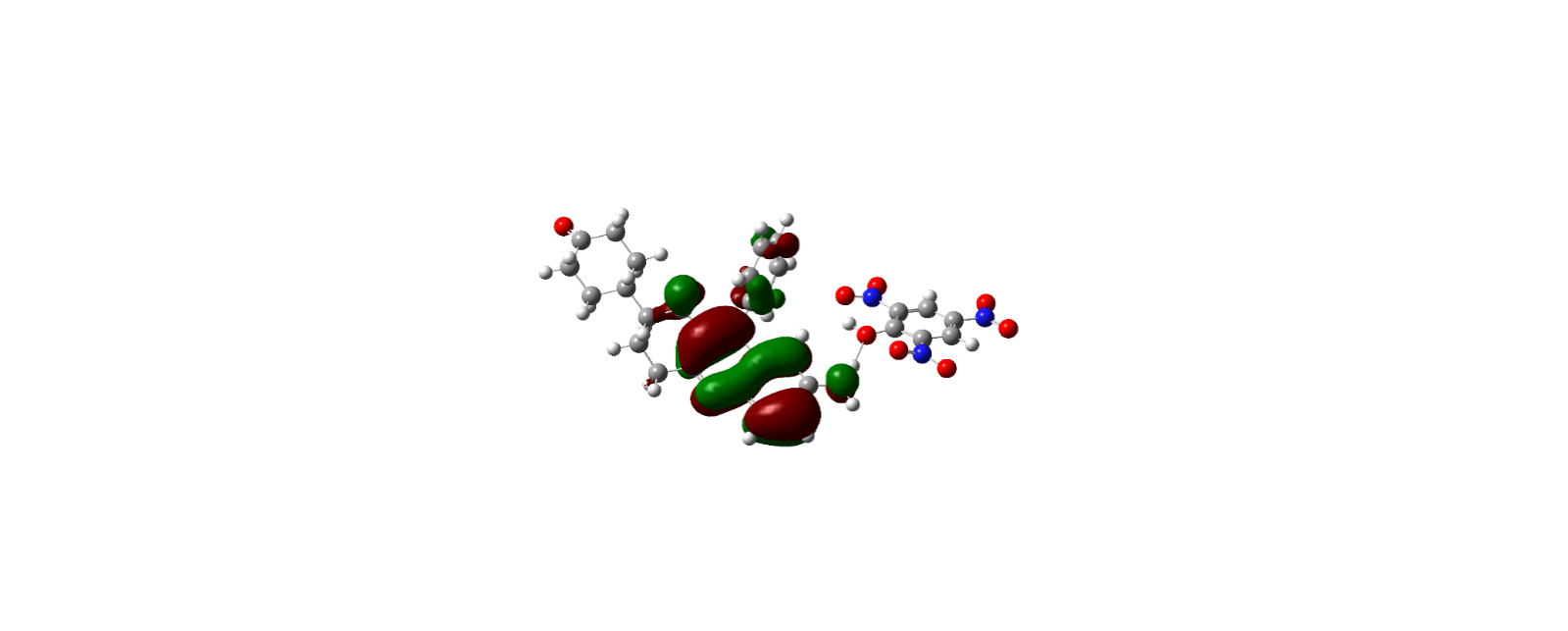 |
| 155 | HOMO-2 | -6.33 | 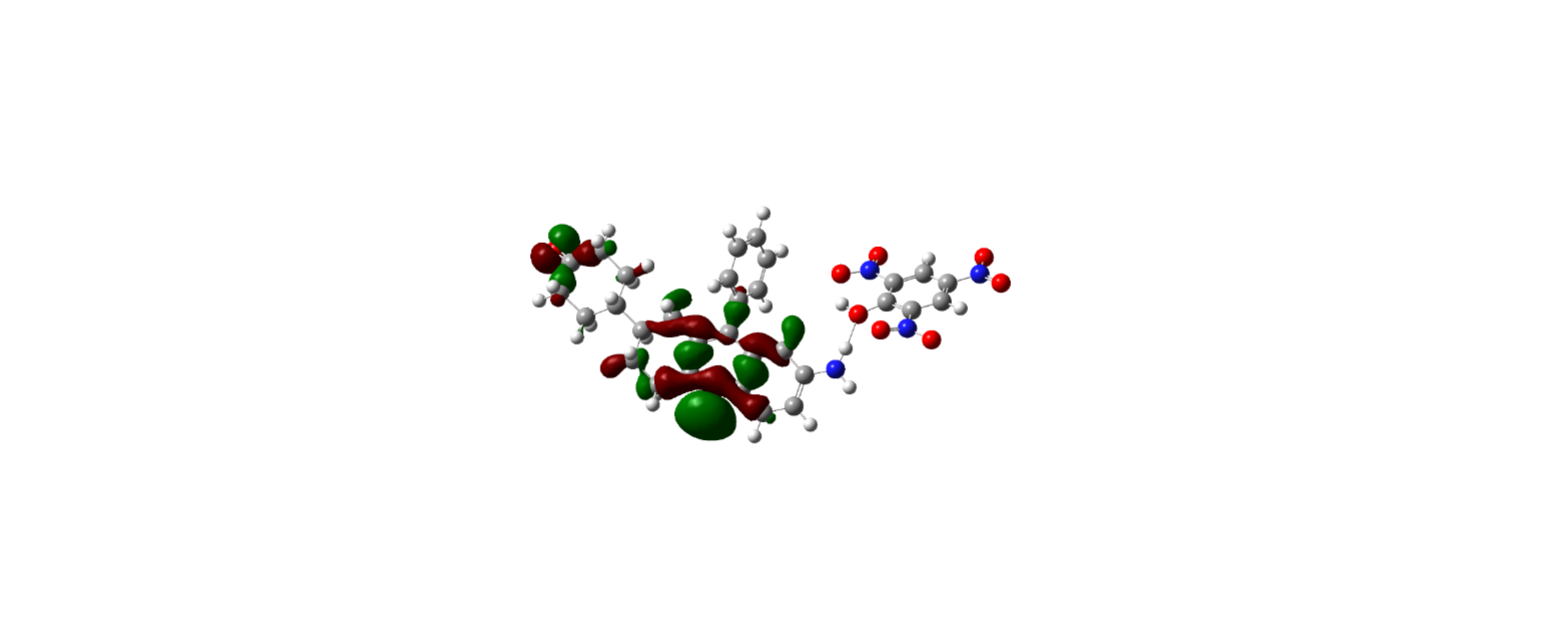 |
| 154 | HOMO-3 | -6.45 | 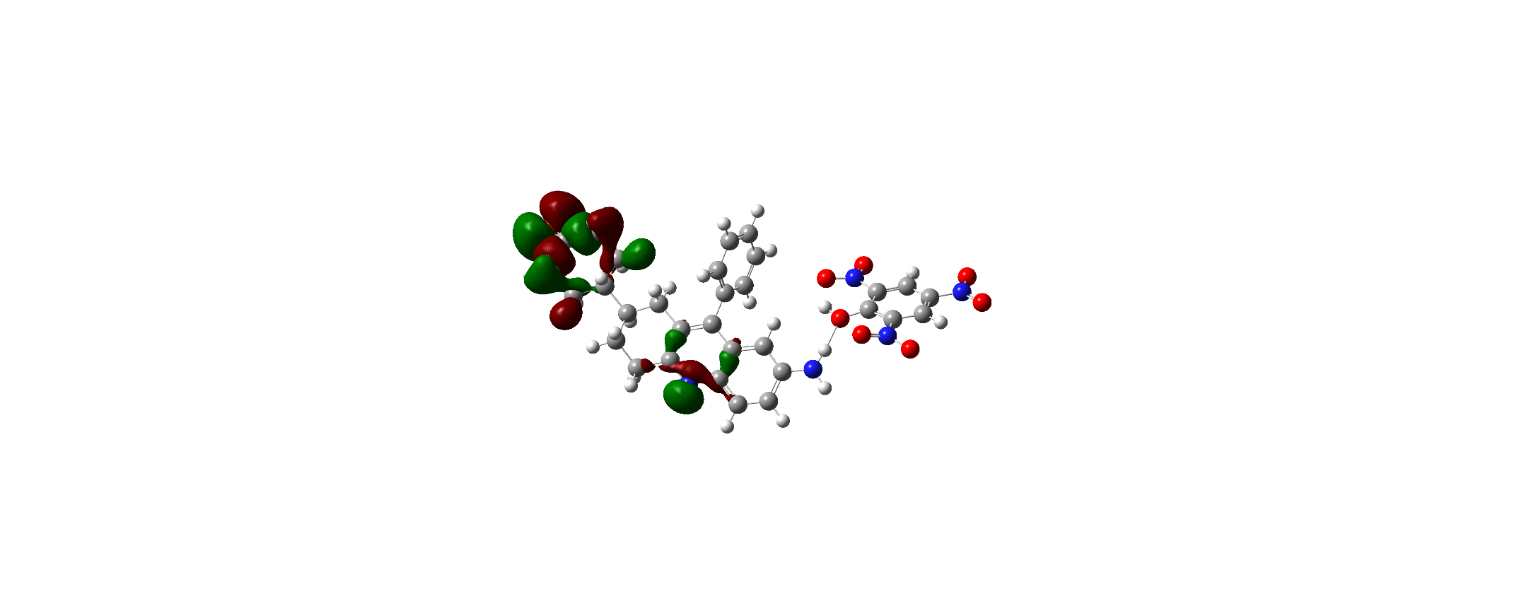 |
| 153 | HOMO-4 | -6.94 | 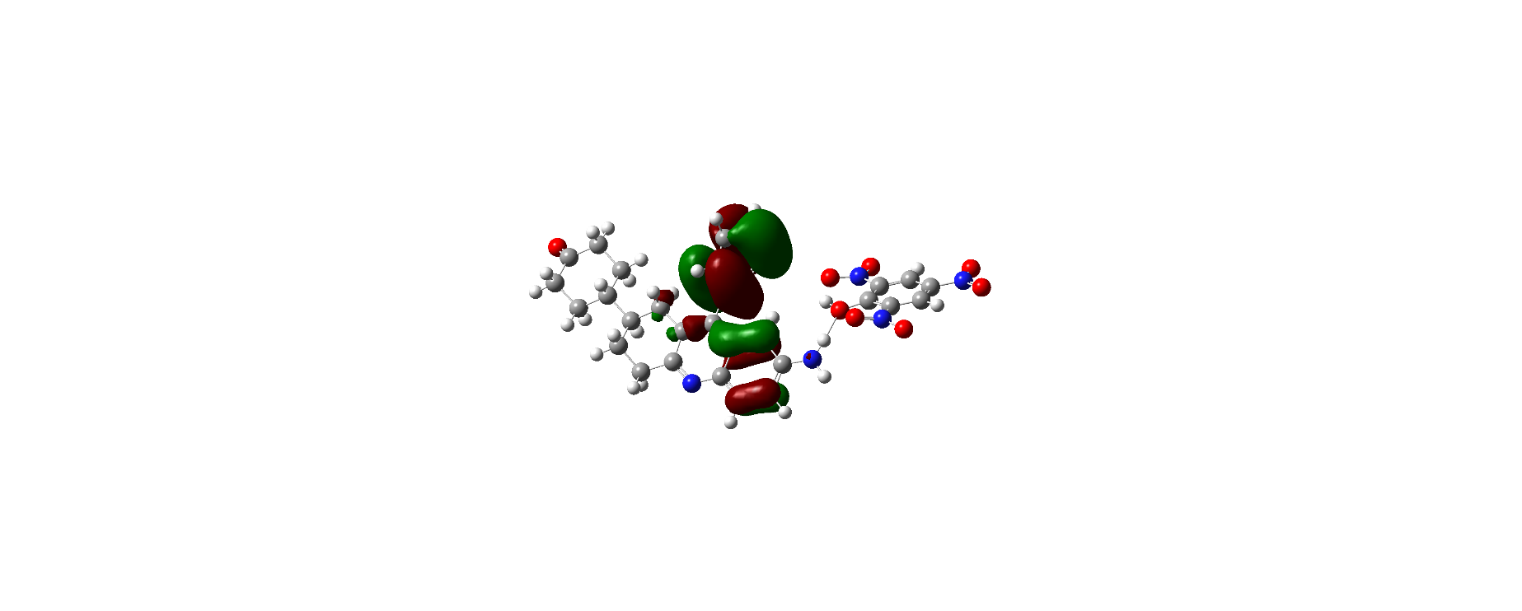 |
| 152 | HOMO-5 | -7.08 | 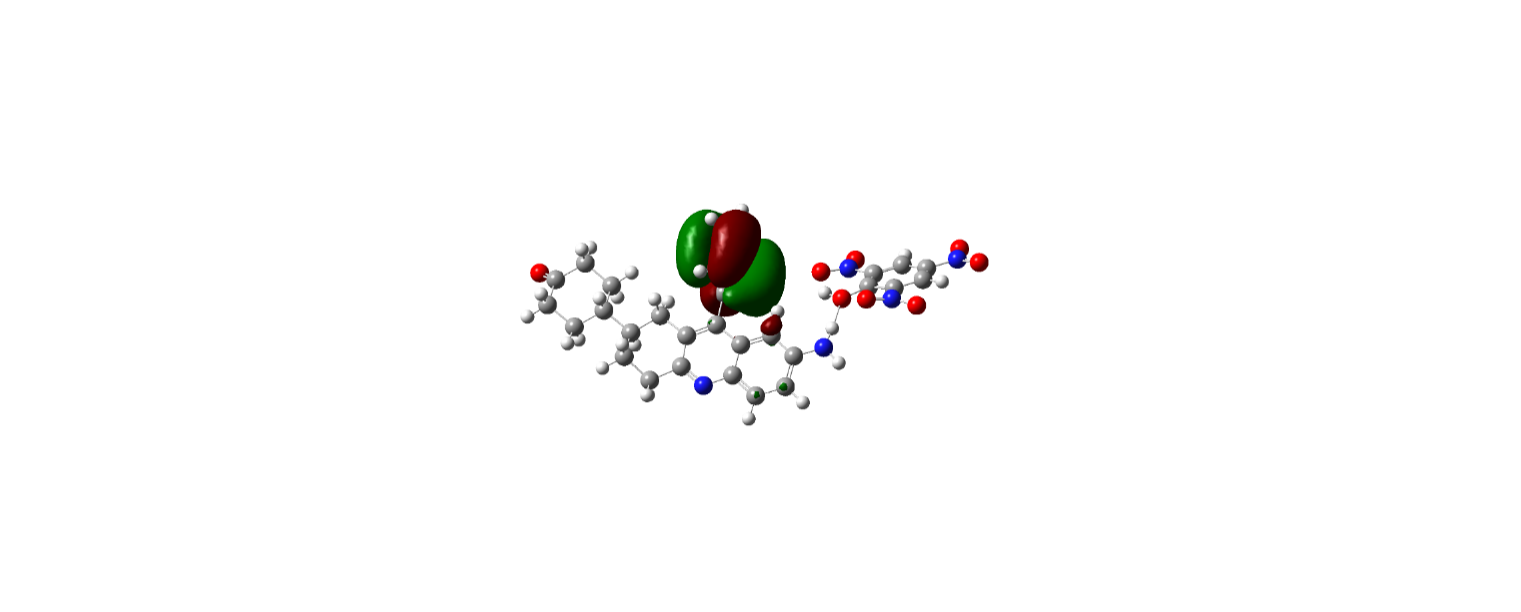 |
| 151 | HOMO-6 | -7.55 | 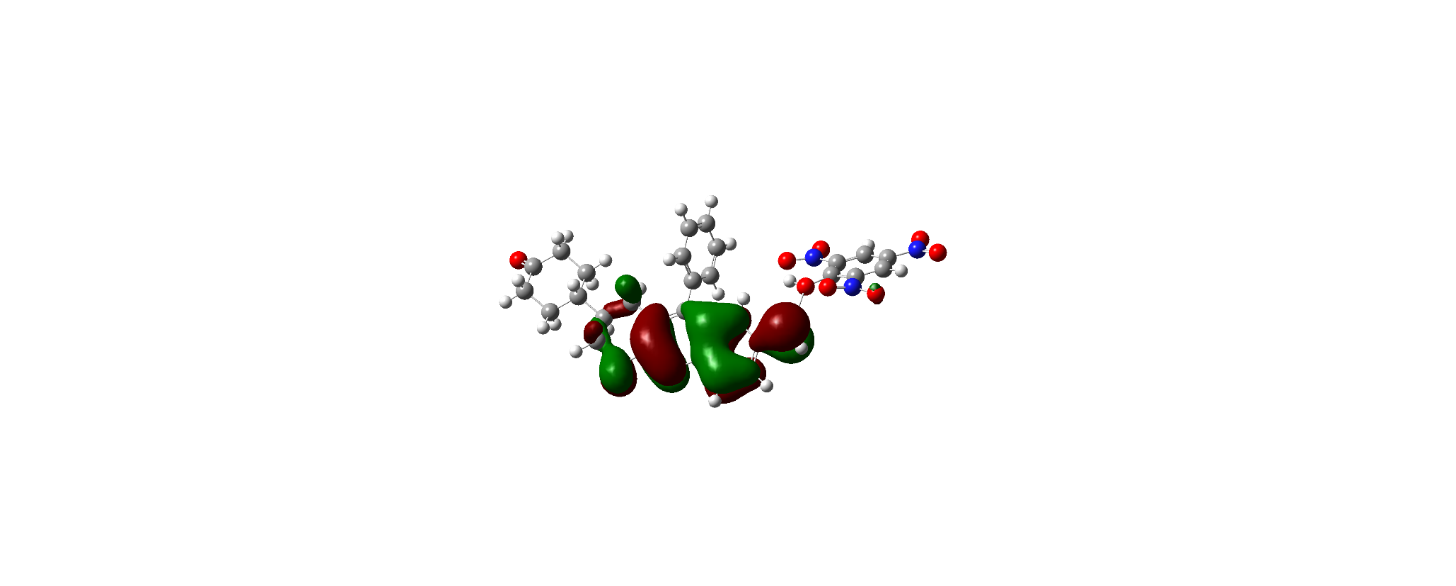 |
| 150 | HOMO-7 | -7.92 | 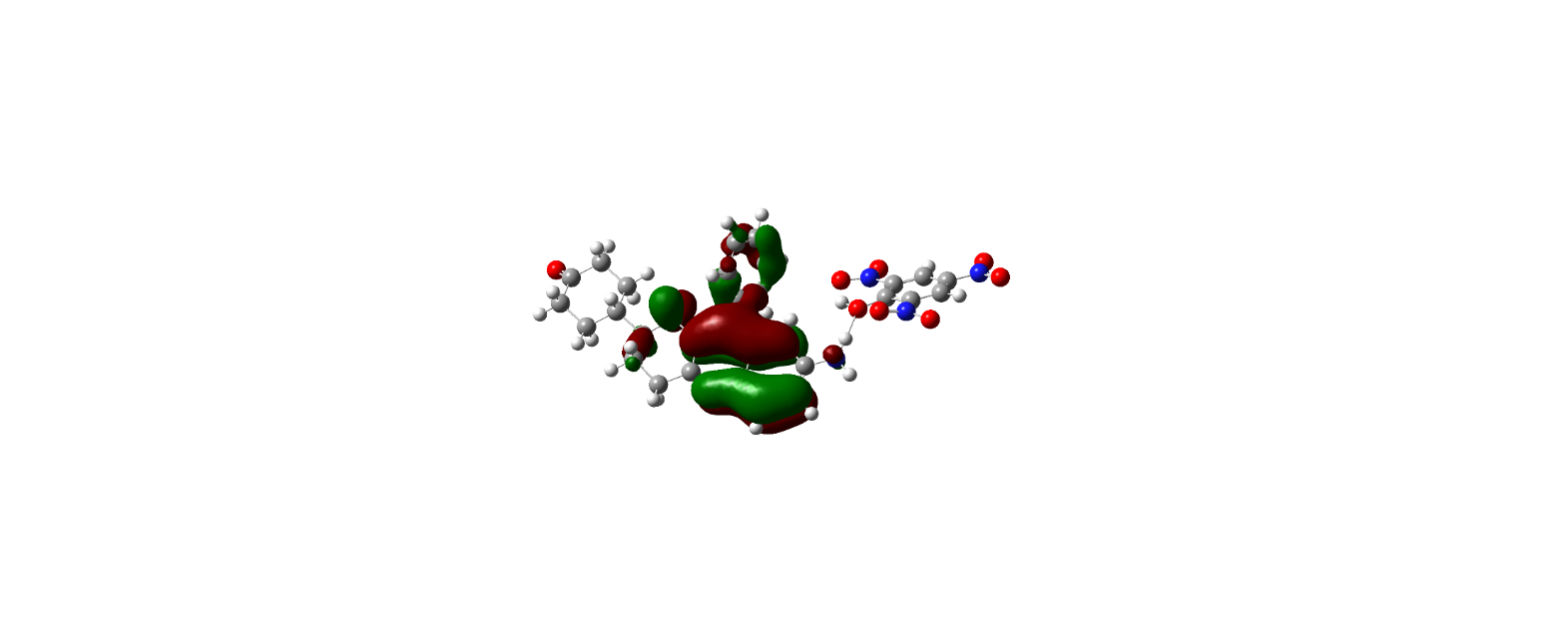 |

**References**

1. Sayan S, Avik D, Arijit G, Avik G, Kaushik B, Krishna Su D, Sohel A, Nakul C. Maiti, Abhijit K D, Benu B D, Raju M. Pyridine-pyrazole based Al (III) ‘turn on’ sensor for MCF7 cancer cell imaging and detection of picric acid. RSC Adv. 2021; 11: 10094-10109 <https://doi.org/10.1039/D1RA00082A>.
2. Yanjiao J, Panpan L, Shangying L, Xian Z, Cuiping L, Ji L, Xuelian X, Liangliang Z. Highly effective detection of picric acid by a Ca(II)-Framework with adjustable crystal morphology and size. J. Solid State Chem. 2022;316 123561. <https://doi.org/10.1016/j.jssc.2022.123561>.
3. Vineet S, Mohan S M. Rapid optical sensor for recognition of explosive 2,4,6-TNP traces in water through fluorescent ZnSe quantum dots. SAA. 2021; 260: 119937. <https://doi.org/10.1016/j.saa.2021.119937>.
4. Pawan K, Dheeraj A, Deepak N, Ajeet S, Amrita G, Amilan J. Dual colorimetric sensor for picric acid and pyrophosphate: Practical application for molecular logic gates, Dyes Pigm. 2019; 166: 443-450. <https://doi.org/10.1016/j.dyepig.2019.03.058>.
5. Ravi P, Shilpa B, Rajender K, Suban K S. Rapid optical sensor for recognition of explosive 2,4,6-TNP traces in water through fluorescent ZnSe quantum dots. Nano-Struct. Nano-Objects. 2019;19:100345. <https://doi.org/10.1016/j.nanoso.2019.100345>.
6. Das S, Das M, Bag A, Laha S, Samanta B C, Choudhury I, Bhattacharya N, Maity T. Selective recognition of Zn (II) by a novel Schiff base chemosensor with the formation of an AIE active Zn (II) complex having picric acid detection ability: Application in live cell imaging study. J. Photochem. Photobiol. A. 2024; 447: 115214 .<https://doi.org/10.1016/j.jphotochem.2023.115214>.
7. Gitanjali J, Navneet K. Fluorimetric quantification of picric acid in aqueous medium via smartphone and invisible ink applications using pyrene-based sensor, Inorg. Chem. Commun. 2022; 140: 109481. <https://doi.org/10.1016/j.inoche.2022.109481>.
8. Goel A, Malhotra R. Efficient detection of Picric acid by pyranone based Schiff base as a chemosensor.J.Mol.Struct. 2022;1249:131619.<https://doi.org/10.1016/j.molstruc.2021.131619>
9. Cui F, Xie Z, Yang R, Zhang Y, Liu Y, Zheng H, Han X. Aggregation-induced emission enhancement (AIEE) active bispyrene -based fluorescent probe: “turn-off” fluorescence for the detection of nitroaromatics.  SAA. 2024;314: 124222. <https://doi.org/10.1016/j.saa.2024.124222>.
